# Genome-Wide Comparative Analyses of Polyadenylation Signals in Eukaryotes Suggest a Possible Origin of the AAUAAA Signal

**DOI:** 10.3390/ijms20040958

**Published:** 2019-02-22

**Authors:** Zhixin Zhao, Xiaohui Wu, Guoli Ji, Chun Liang, Qingshun Quinn Li

**Affiliations:** 1 College of Biopharmaceutical and Food Engineering, Shangluo University, Shangluo 726000, China; zxzhao@slxy.edu.cn; 2 Department of Biology, Miami University, Oxford, OH 45056, USA; liangc@miamioh.edu; 3 Department of Automation, Xiamen University, Xiamen 361005, China; xhuister@xmu.edu.cn (X.W.); glji@xmu.edu.cn (G.J.); 4 Key Laboratory of the Ministry of Education for Coastal and Wetland Ecosystems, and College of the Environment and Ecology, Xiamen University, Xiamen 361102, China; 5 Graduate College of Biomedical Sciences, Western University of Health Sciences, Pomona, CA 91766, USA

**Keywords:** polyadenylation signal, RNA processing, mRNA 3′-end formation, bioinformatic analysis

## Abstract

Pre-mRNA cleavage and polyadenylation is an essential step for almost all mRNA in eukaryotes. The *cis*-elements around the poly(A) sites, however, are very diverse among different organisms. We characterized the poly(A) signals of seven different species, and compared them with that of four well-studied organisms. We found that ciliates do not show any dominant poly(A) signal; a triplet (UAA) and tetramers (UAAA and GUAA) are dominant in diatoms and red alga, respectively; and green alga *Ostreococcus* uses UGUAA as its poly(A) signal. Spikemoss and moss use conserved AAUAAA signals that are similar to other land plants. Our analysis suggests that the first two bases (NN in NNUAAA) are likely degenerated whereas UAAA appears to be the core motif. Combined with other published results, it is suggested that the highly conserved poly(A) signal AAUAAA may be derived from UAA with an intermediate, putative UAAA, following a pathway of UAA→UAAA→AAUAAA.

## 1. Introduction

Messenger RNA (mRNA) 3′-end formation, including cleavage and polyadenylation, is a crucial step during mRNA post-transcriptional processing in eukaryotes. Polyadenylation is essential for eukaryotic gene expression because of its biological functions, including the protection of mature mRNA from unregulated degradation and recognition of mature mRNA by cytoplasm export machinery and translational apparatuses [1,2]. Moreover, the formation of the 3′-end is an integral part for transcription termination of RNA polymerase II coupled with cleavage factors that utilize polyadenylation signals on pre-mRNAs [3]. In recent years, polyadenylation has been increasingly recognized as an important regulator in eukaryotic gene expression [4,5].

Polyadenylation signals are *cis*-elements surrounding the cleavage sites (or poly(A) sites) that are recognized by the polyadenylation protein factor complex and direct both cleavage and polyadenylation reactions. They include four different elements: the far upstream element (FUE), defined here as it is located furthest (>35 nucleotides) upstream from the cleavage site; the near upstream element (NUE), located ~10 to 35 nt upstream of cleavage site, also known as the equivalent of AAUAAA in many species; the cleavage site and its immediate surrounding motifs called cleavage element (CE); and the downstream element commonly found only in animals and located downstream of the cleavage site [6,7,8,9]. Among these different elements, NUE appears to be the strongest signal for both cleavage and polyadenylation reactions, because the cleavage and polyadenylation specificity factor complex has been shown experimentally to bind to this region, especially with the AAUAAA signal [2,7,10].

In animals, the highly conserved polyadenylation signals are AAUAAA and its one- or two-nucleotide variants (e.g., AUUAAA) located in the NUE region. In humans, 82.5% of 18,277 mRNA transcripts possess the canonical hexamer AAUAAA or its 11 single-nucleotide variants [11]. Similar results were also reported in other studies of the human and mouse [12]. Recently, 87% of mRNA transcript isoforms in *Caenorhabditis elegans* were detected with AAUAAA or its one/two nucleotide(s) variants in 3′UTR regions [13]. Although AAUAAA was not shown with high frequency in plants, this hexamer was still ranked as the first on the NUE signal list, and detected in ~10% transcripts in *Arabidopsis* [14] and ~7% in rice [15], respectively. Graber et al. [16] also found that universal and conservative AAUAAA varied widely among six eukaryotic species (yeast, rice, *Arabidopsis*, fruit flies, mice and humans). Utilizing direct RNA sequencing technology, abundant unannotated poly(A) sites were revealed in humans and yeast, and TTTTTTTTT and AAWAAA (W = A/T) were identified as novel motifs in the upstream of poly(A) sites [17].

In the green algae *Volvoxcarteri carteri* and *Chlamydomonas reinhardtii*, UGUAA was reported as the poly(A) signal in the NUE region in alpha 2- and beta 2-tubulin-encoding genes [18]. This pentamer appeared to be the most frequent motif (31.8%) within 40 nt upstream from poly(A) sites in chrysophycean alga *Ochromonas danica* [19]. In *C. reinhardtii*, UGUAA was also found to be the most significant and conserved poly(A) signal in the NUE regions in over 4000 genes [20] and APA (alternative polyadenylation) influenced up to 68% of the genes [21]. Meanwhile, poly(A) signals showed a considerable variation among different green algal species: the UGUAA signal was completely lost in streptophyte alga *Pyramimonas*, and the NUE region was U-rich instead of A-rich in most algal species investigated [22]. Interestingly, wet-lab experiments demonstrated that UGUAA was the specific motif recognized and bound by Cleavage Factor I in human mRNA 3′-end processing [23,24]. However, UGUAA was not a strong poly(A) signal in humans compared to AAUAAA [20]. Moreover, tetramer UAAA was found to be a poly(A) signal in parasitic protozoan *Trichomonas vaginalis* mRNAs, suggesting a possible cooperation between translational stop codons (UAA) and signaling for mRNA 3′-end processing [25,26].

Although the two conserved poly(A) signals UGUAA and AAUAAA have been computationally detected and experimentally verified in some cases, their relationships and evolutionarily developed patterns have not been systematically studied yet. This study is aimed at exploring the poly(A) signals in seven species whose poly(A) signals have not been investigated, and examining the change of poly(A) signals among eukaryotes spanning a wide evolutionary range. This comparative study provides valuable insights into the potential origin of poly(A) signals, and expands our understanding of the molecular, biological and evolutionary mechanisms regulating mRNA polyadenylation processes in eukaryotes.

## 2. Results

### 2.1. Single Nucleotide Profiles Around Poly(A) Sites in Seven Species

**Figure 1:**
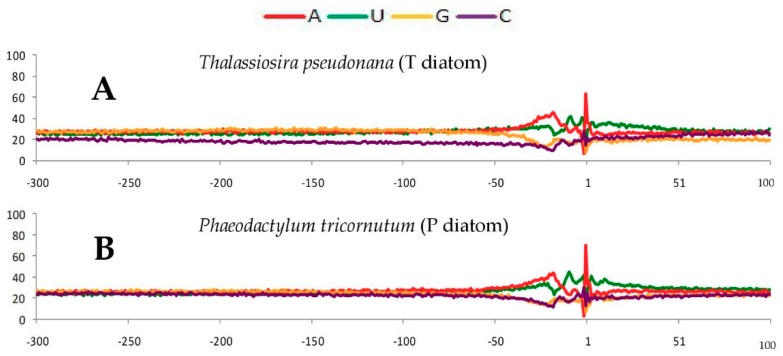

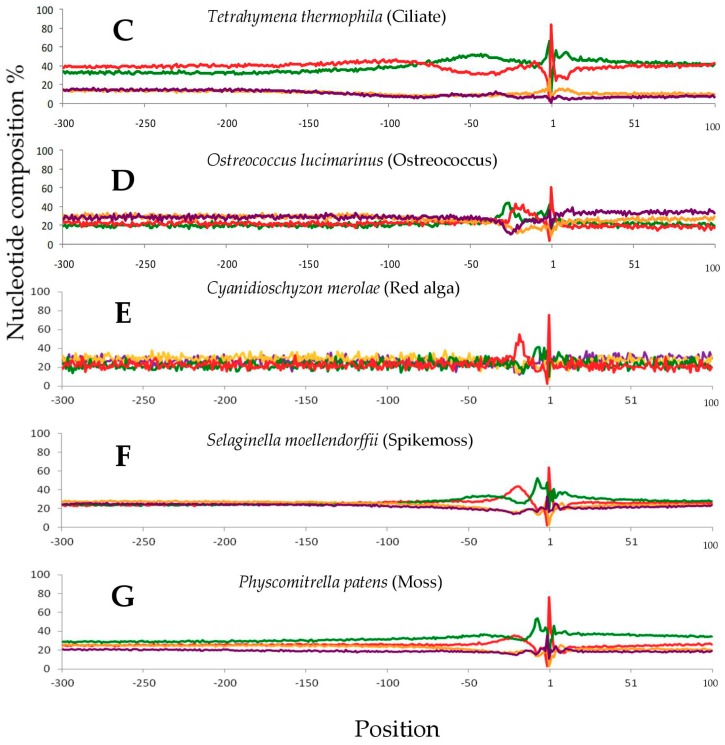
The single nucleotide profiles around poly(A) sites for the seven species. The −1 position is poly(A) site, “−” is designated as the upstream sequences (300 nt), and ‘‘+’’ represents the downstream sequences (100 nt). (**A**) diatom *Thalassiosira pseudonana*, (**B**) diatom *Phaeodactylum tricornutum*, (**C**) ciliate *Tetrahymena thermophila*, (**D**) green alga *Ostreococcus lucimarinus*, (**E**) red alga *Cyanidioschyzon merolae*, (**F**) spikemoss *Selaginella moellendorffii*, (**G**) moss *Physcomitrella patens*.

To study the wide range eukaryotic species for their poly(A) signals, EST (expressed sequence tags) data from relevant species were collected, and SignalSleuth2 was developed to perform exhaustive searches of short sequence motifs in a specified range of nucleotide sequences with variable motif sizes (generally 3−8 nt in length) and rank the detected motifs based on their frequencies of occurrence (see Section 4 for details). We first studied these seven species whose poly(A) signals were not characterized previously: diatoms *Thalassiosira pseudonana* and *Phaeodactylum tricornutum*, ciliate *Tetrahymena thermophila*, green alga *Ostreococcus lucimarinus*, red alga *Cyanidioschyzon merolae*, spikemoss *Selaginella moellendorffii* and moss *Physcomitrella patens*. It has been demonstrated that single nucleotide profiles around poly(A) sites were unique in comparison to other parts of transcription units [14,15,20]. Therefore, 400 nt (i.e., −300 nt to +100 nt, where poly(A) site is defined as −1 position) genomic sequences around poly(A) sites were extracted for poly(A) signal analysis. Firstly, the single nucleotide profiles of all seven species are displayed in Figure 1. The average nucleotide frequencies in the whole 400-nt region as well as in FUE, NUE and CE regions are shown in Table S1. In particular, the positions of NUE were determined based on the changes of nucleotide profiles near poly(A) sites and the distribution regions of conserved signals, while FUE and CE elements are set to be ~100 nt upstream and ~20 nt downstream of the determined NUE elements, respectively (see Section 4 for details). Based on both average nucleotide frequencies and single nucleotide profiles, especially in the FUE and NUE regions, the seven species show interesting differences and similarities:

(1) Two diatoms (*T. pseudonana* and *P. tricornutum*). As shown in Table S1, the two diatoms have similar nucleotide frequencies in the whole 400 nt regions, and there is a common trend of A>U>G>C. In the NUE region for both diatoms (Figure 1A,B), A and U increase almost concordantly at first, then decrease after reaching a peak.

(2) Ciliate (*T. thermophila*). As shown in Figure 1C, ciliates show a unique single nucleotide profile, in which A-richness is evident from −300 to −80 followed by a U-rich region from −80 to −13.

(3) Green alga (*O. lucimarinus*). There are higher G and C contents, especially in the FUE region (see Table S1). In the NUE region (Figure 1D), a U-peak is followed concordantly by an A-peak.

(4) Red alga (*C. merolae*). Like diatom *P. tricornutum* (Figure 1B), the contents of the four nucleotides are similar to each other in the FUE region in red alga (Figure 1E). In particular, there is a striking A-peak and three similar U-, G- and C-troughs in the NUE region (Figure 1E). The roughness of the single nucleotide profile reflects the lack of sequence data available for our poly(A) signal analysis about this species.

(5) Spikemoss (*S. moellendorffii*) displays a unique single nucleotide profile in the FUE region that is different from other species. There seems to be a transition for dominant single nucleotides from G (−200 to −110) to U (−110 to −32) in the FUE region (see Table S1 and Figure 1F). This indicates that spikemoss may sit between green alga *C. reinhardtii* (possessing the highest G content) and moss (possessing the highest U content) in terms of nucleotide variation in FUE. The striking A-peak and relative deeper U-trough are also detected in this NUE region (Figure 1F).

(6) Moss (*P. patens*). There are dominant U contents in the FUE regions; the single nucleotide profile in the NUE regions is similar to that in spikemoss: an A-peak and a relatively obvious U-trough in comparison to its G- and C- troughs (Figure 1G).

### 2.2. Putative Polyadenylation Signals Revealed in the Seven Species

Our analysis shows no clear dominant pentamer (e.g., UGUAA) or hexamer (e.g., AAUAAA) detected in the NUE region in the two diatoms. In contrast, a triplet UAA is extremely dominant (86.51% and 78.21%) with significant Z-Scores (see Section 4 for definition) of 19.12 and 6.94 for *T. pseudonana* and *P. tricornutum*, respectively (Table 1). As shown in Figure S1A,B, the dominancy of triplet UAA is also evident in the positional distribution profiles across the entire NUE region of the two diatom species. No significantly frequent motif of 3-nt (triplet) to 8-nt (octamer) is detected in the NUE region in ciliates (Table 1). This is consistent with the fact that no single dominant signal stands out in its positional distribution profile (Figure S1C), which is obviously different from the other six species described herein. UGUAA is the most prominent pentamer in the NUE region of the green alga *O. lucimarinus* (30.4% frequency with a Z-Score of 30.21, Table 1). This is also evident in the UGUAA positional distribution profile in the NUE region shown in Figure S1D. Although AAUAA is found in the top 50 hexamers, this sequence shows a very low frequency (only 4.21%) with a Z-Score that is not significant enough to be reported by RSAT [27]. Accordingly, it is inferred that AAUAAA could not be a significantly conserved poly(A) signal like UGUAA in green alga *O. lucimarinus*. In red alga, UAAA and GUAA appear to be more frequent in the NUE region (Table 1 and Figure S1E): UAAA is detected in 86.45% transcripts with a significant Z-score of 15.33 while GUAA is found in 43.23% transcripts with a substantial Z-score of 8.78. In spikemoss, the most frequently occurred motif detected in its NUE region was the canonical hexamer AAUAAA (see Figure S1F), although its frequency is still low (7.83%, with a Z-Score of 7.18). In moss, AAUAAA also proved to be the most frequent motif (7.25%, with a significant Z-Score of 15.46) in the NUE region (Table 1), standing out obviously from other hexamers in its positional distribution profile shown in Figure S1G.

In the FUE region, there is no obvious or conserved individual signal reported previously in Arabidopsis, rice or green alga *C. reinhardtii* [14,15,20]. In this study, the top 50 signals (from triplets to octamers) in diatom *T. pseudonana* are found to be AG-rich in the FUE region and then UG-rich in the FUE-NUE junction region (−80 to −35) (see supplementary Table S2 and Figure S2A). Similar to diatom *T. pseudonana*, the top 50 signals detected in the FUE region of diatom *P. tricornutum* are also AG-rich (see Table S3 and Figure S2B). In ciliates, the most frequently occurred motifs in the FUE region are UA-rich (Figure S2C), which are consistent with its single nucleotide profile (see Table S4 and Figure 1C). Green alga *O. lucimarinus* does not show any significantly frequent motifs in the FUE region, but many CG-rich signals are shown in the top 50-motif list with stable distribution across the whole FUE region (see Table S5 and Figure S2D). Red alga does not show any significantly frequent motif in the FUE region (see Table S6 and Figure S2E). In spikemoss and moss, the highest overall frequencies of the top 50 motifs (from triplets to octamers) are detected in the FUE-NUE junction regions (Figure S2F,G; Tables S7 and S8). In spikemoss, AAG-rich and UUC-rich pentamers are common in the FUE region. The predominant pentamer signals are AAGAA (44.17%, 5.15 Z-Score), UUCUU (40.43%, 5.96 Z-Score) and GAAGA (39.65%, 5.69 Z-Score) in −150 to −80 region, where a transitional change from G-rich to U-rich is evident in single nucleotide profile (Figure 1F). In moss, no motif has proven to be significantly frequent in the FUE region, but top motifs are U-rich and the overall frequencies of the top 50-motifs increase obviously in the region between −70 and −35 (see Table S8 and Figure S2G).

### 2.3. Conservative Nucleotide Composition Around Poly(A) Sites in the Seven Species

Through investigating the nucleotide composition in the CE region, we found that BA dinucleotide (−2 to −1, B = U/G/C) is dominant in frequencies and conserved in all seven investigated species (Figure S3). BA is actually an extension of YA (−2 to −1, Y = U/C) [14] but the average frequency of BA (70.45%) is much higher than that of YA (53.48%), and the frequency distributions show that BA is obviously higher than YA in the seven species demonstrated in box-plots using R analysis (http://www.r-project.org/, see Figure S4).

### 2.4. The Frequency Variations of Polyadenylation Signals in NUE

**Figure 2:**
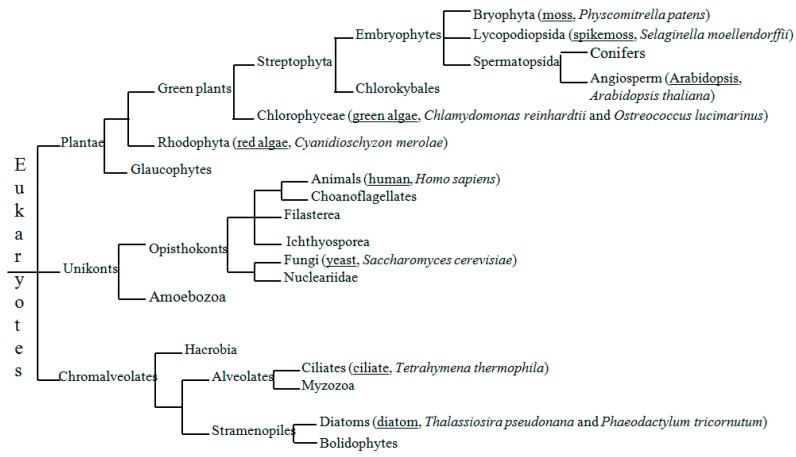
The phylogenetic relations among the 11 species investigated in our study. The common names and scientific names are listed in parentheses close to the relevant clade names in the phylogenetic tree. The common names are all underlined. The phylogenetic tree is constructed according to Tree of Life Web Project (http://tolweb.org/tree/phylogeny.html).

Our study shows that UAA, UAAA, GUAA, UGUAA and AAUAAA are the major motifs detected in the NUE regions of most of the seven species, with variable degrees of frequency and significance. Moreover, the poly(A) signals in model organisms have been frequently examined, including green alga *Chlamydomonas reinhardtii* [20,22], yeast *Saccharomyces cerevisiae* [16,17], *Arabidopsis* (*Arabidopsis thaliana)* [14,28] and humans *Homo sapiens* [12,17]. As shown in Figure 2, after the addition of the four model organisms the investigated species spanned a large evolutionary distance, from Chromalveolates (ciliates and two diatoms) and Unikonts (yeast and human), to Plantae (red alga, two green algae and embryophyte-land plants). Meanwhile, some of the species are closely related and represent small evolutionary distances. Thus, the compared species include two diatoms (*T. pseudonana* and *P. tricornutum*), two green algae (*C. reinhardtii* and *O. lucimarinus*), red alga (*C. merolae*), ciliate (*T. thermophila*), moss (*P. patens*), spikemoss (*S. moellendorffii*), *Arabidopsis* (*A. thaliana*), yeast (*S. cerevisiae*) and humans (*H. sapiens*). This comparative study is set up to understand the poly(A) signal changes between species with large and small evolutionary distances.

**Figure 3:**
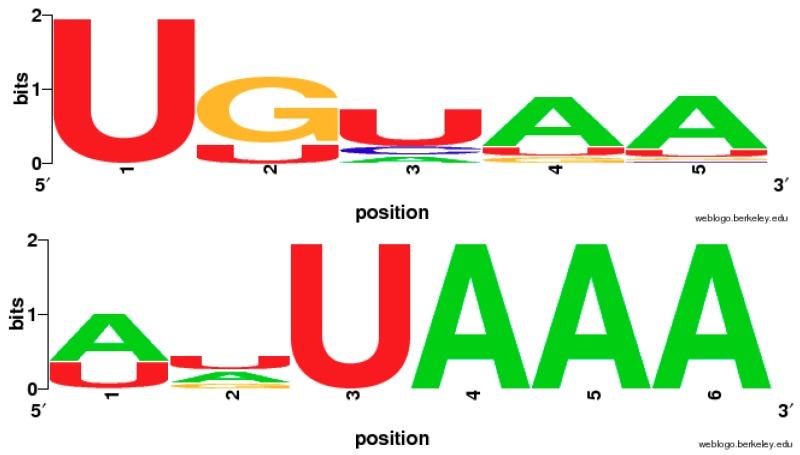
The combined logos of mononucleotide variants from the UGUAA group (top) and two AAUAAA groups (bottom).

In addition to the two canonical poly(A) signals UGUAA and AAUAAA, the mono- or dinucleotide variants of the poly(A) signals were also suggested to affect the polyadenylation process [11,12,13]. In our study, the single nucleotide variants of UGUAA and AAUAAA were extracted and compiled from the top 100 (considering the low frequencies of their single nucleotide variants in the top 50) frequent pentamers and hexamers respectively. As shown in Table 2, which lists the frequencies of UGUAA and AAUAAA and their single nucleotide variants, the combined frequency of mononucleotide variants of UGUAA in green algae is ~22% for *C. reinhardtii* and ~29% for *O. lucimarinus*, both of which belong to the UGUAA group (named after the UGUAA signal). Similarly, the combined frequency of mononucleotide variants of AAUAAA is ~25% for humans (Table 2). In spikemoss, moss and *Arabidopsis*, these combined frequencies are 5%~7%, and the overall frequencies that include both AAUAAA and its single-nucleotide variants are still less than 15%. Figure 3 shows the sequence logos for the consensus sequences of single nucleotide variants based on their frequencies for the UGUAA group and two AAUAAA groups by WebLogo [29]. UAAA appears to be the core part of AAUAAA whereas the first two bases likely degenerate.

### 2.5. The Variation of Canonical Polyadenylation Signals (UGUAA and AAUAAA) in the 11 Species

**Figure 4:**
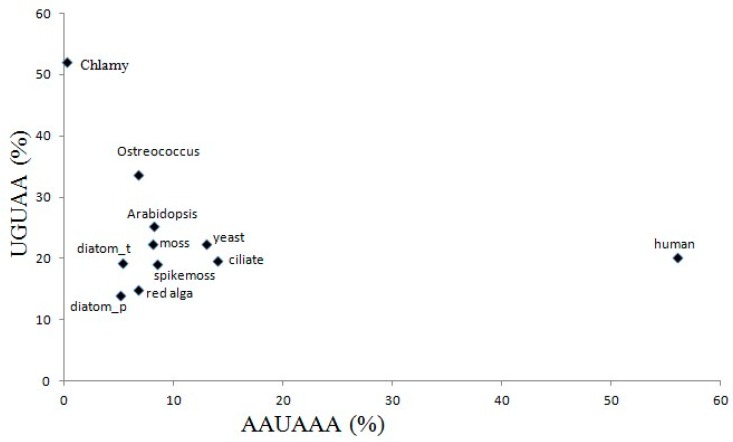
The overall frequencies of UGUAA and AAUAAA in a 2-D coordinate in the 11 species. Abbreviations: chlamy, green alga *C. reinhardtii*; Ostreococcus, green alga *O. lucimarinus*; diatom_t, diatom *T. peudonana*; diatom_p, diatom *P. tricornutum*.

Among the 11 species, only green algae *C. reinhardtii* and *O. lucimarinus* have significantly dominant amounts of the pentamer UGUAA, which is mainly distributed in the NUE regions. Although the other 9 species do not have significantly dominant UGUAA signals, they do show between-species differences in UGUAA frequencies. In the two-dimensional coordinate graph shown in Figure 4, the overall frequencies of UGUAA (from −80 to −15 region) and AAUAAA (from −50 to −15 region) demonstrate that humans and the two green algae are located on the furthest end of the X and Y axes, respectively. Interestingly, humans not only possess the highest AAUAAA (56%), but also have intermediate UGUAA frequencies (20.09%). Green alga *C. reinhardtii* only show the highest UGUAA frequency (50%) with the lowest AAUAAA frequency (0.21%). Green alga *O. lucimaritus*, which also utilizes UGUAA as its major poly(A) signal (40%), shows AAUAAA in a relatively low frequency (6.77%). The remaining eight species can be divided into roughly two groups: (1) diatoms and red alga, which have similar AAUAAA (5–6%) and UGUAA (14–19%) frequencies and (2) land plants (embryophytes), yeast and ciliates, which have similar AAUAAA (8–14%) and UGUAA (19–25%) frequencies.

## 3. Discussion

Polyadenylation proves to be an important post-transcriptional process to mRNA maturation, cytoplasm exportation and protein translation [1,2]. Poly(A) signals near poly(A) sites are critical in defining the location of the cleavage and adenine addition on pre-mRNA. Both UGUAA and AAUAAA poly(A) signals have been computationally detected and experimentally verified in some eukaryotes as conserved *cis*-regulatory motifs that can be recognized and bound by the polyadenylation complex to conduct polyadenylation [5,15,20,22,30]. In this study, we examined and characterized the putative poly(A) signal profiles from 11 species spanning a large evolutionary distance. Seven of these species were previously unstudied in terms of poly(A) signal analysis. Such comparative poly(A) signal analysis will facilitate our understanding of the potential evolutionary patterns of poly(A) signal usage and variation.

### 3.1. The Usage and Distribution of Poly(A) Signals in the NUE Region

The seven species show great differences in frequency and significance in both the conserved poly(A) signals (i.e., UGUAA and AAUAAA) as well as other putative poly(A) signals (i.e., UAA, GUAA and UAAA) in their NUE regions. As shown in Figure S1 and Table 1, ciliates do not have a conserved signal. Surprisingly, no significantly frequent pentamer (UGUAA) or hexamer (AAUAAA) was found in the two diatoms (*T. pseudonana* and *P. tricornutum*) and red alga (*C. merolae*). Instead, as shown in Figure S1, a triplet UAA shows dominant frequency (~80%) and strong significance, especially in diatom *T. pseudonana* (Z-Score = 19.12), in comparison with other triplets. So far there has been no direct experimental evidence supporting triplets as a poly(A) signal, because it is generally believed that three-nucleotide motifs are too short to be bounded by polyadenylation factors. Our results, however, suggest that the triplet UAA (stop codon) might be an ancestral motif that may have acted as a foundation for functional poly(A) signals like AAUAAA and UGUAA; whether UAA could function both in polyadenylation and protein translation simultaneously deserves further research.

In red alga (*C. merolae*), UAAA and GUAA are found having significantly higher frequencies than other tetramers in the NUE region. Interestingly, UAAA was also reported to be a conserved poly(A) signal in protozoan parasite *Trichomonas vaginalis* mRNAs [25,26]. Our research not only supports the hypothesis that UAAA might be the intermediate to AAUAAA’s evolutionary process [25], but further suggests that UAA may be the initial ancestral signal of UAAA. Both previous studies [20,22] and our work demonstrate that green algae *C. reinhartdii* and *O. lucimarinus* mainly use UGUAA as poly(A) signal in the NUE region. Yet in another green alga *Scherffelia dubia*, AAUAAA was found to be loosely distributed in the NUE region [22]. Spikemoss and moss utilize the canonical signal AAUAAA (~8% in frequency) with high significance (Z-Score >8), which is similar to streptophyta, land plants and animals [2,7,12,14,22,28,31]. AAUAAA seems to be a major poly(A) signal widely used in eukaryotes, while UGUAA could also be utilized as the core poly(A) signal in some eukaryotes.

If AAUAAA does not have high frequency in the NUE region, then UGUAA might be used frequently. This was specially exemplified in green alga *C. reinhardtii* (see Figure 4), which utilizes UGUAA (~53%), rather than AAUAAA (<1%) as its major signal, suggesting that AAUAAA might not be a necessary poly(A) signal in some species. Interestingly, human genes not only use AAUAAA as its major poly(A) signal but also have relatively high UGUAA frequency (~20%). Moreover, wet-lab experiments have demonstrated that UGUA is a specific motif recognized by CFIm25 in human mRNA 3′-end processing [23,24]. This may explain the high UGUAA frequency in human pre-mRNAs and indicate that UGUAA is necessary for most eukaryotes.

### 3.2. The Poly(A) Signals in the FUE and CE Regions

FUE is defined as a region spanning about 60–100 nt that contains weak *cis*-element signals. In *Arabidopsis*, U-rich motifs appeared to be abundant in the FUE region [14]. The apparent motifs in the FUE region were G-rich in green alga *C. reinhardtii* [20,22]. In our study, among all scanned motifs (3–8 nt in length) in the seven previously unstudied species, we did not detect any individual signals that show significantly high frequencies in the FUE region. However, our results revealed that the top-ranked motifs in FUE regions have certain nucleotide preferences (e.g., AG-rich signals in diatom *T. pseudonana*) when we examine the top 50 ranked motifs of 3–8 nt in size. Such nucleotide preferences appear to be associated with their single nucleotide profiles.

Interestingly, a conserved BA (−2 to −1; B = U/C/G) dinucleotide was found in the CE region of all species except green alga *C. reinhardtii*. YA (−2 to −1; Y = U/C) dinucleotide was found to be conserved around the cleavage site of six eukaryotic species including *Arabidopsis* [14,16]. In our study, GA (−2 to −1) had a high abundance (15%~20%) yet AA (−2 to −1) was extremely low (<5%) in the seven species. Therefore, BA (−2 to −1) should be more inclusive and accurate than YA (−2 to −1) in describing the conserved dinucleotide in the CE region.

### 3.3. The Relationships Between Single Nucleotide Profiles and Poly(A) Signals

The single nucleotide profiles, which show distinctive patterns among the seven species presented herein (Figure 1), appear to be consistent with the frequencies and significances of poly(A) signals detected in the NUE and FUE.

In Chlorophyta (green algae), *Prototheca wickerhamii* and *Scherffelia dubia* were found to possess high G and C contents in the FUE region; the U-peak was ahead of A-peak in the NUE region, and U-rich signals and UGUAA were found dominantly in the FUE and NUE regions, respectively [22]. Such results are consistent with green alga *O. lucimarinus* in our research. Similar to spikemoss results, the patterns that show the dominant single nucleotide profile changing from G to U in FUE were also found in Streptophyta *Closterium peracerosum* and *Klebsormidium subtile* [22]. Furthermore, both our study and previous works suggest that U-rich signals in FUE and AAUAAA signals in NUE are concordantly detected in many eukaryotic species.

Moreover, single nucleotide profiles around poly(A) sites are an indicator of the nucleotide composition of poly(A) signals. For example, a strong A-peak found in the NUE region in humans suggests that its major poly(A) signals are likely to be A-rich motifs (i.e., AAUAAA). In green alga *O. lucimarinus*, a U-rich peak followed concordantly by an A-rich peak in NUE regions would explain why UGUAA is found as their major poly(A) signal. In land plants, A-peak and a relative deep U-trough in NUE regions also explains why AAUAAA is a top poly(A) signal in those species. However, ciliates have no obvious A-peak and red alga does not possess relatively deep U-troughs, suggesting that UGUAA or AAUAAA might not be major poly(A) signal in ciliates or red alga.

### 3.4. The Comparative Analysis of Poly(A) Signals in 11 Species Suggested an Evolutionary Pathway of Poly(A) Signal Variation

Based on the comparative analysis of the putative poly(A) signals in the 11 species (Table 1 and Table 2) and their evolutionary distances (Figure 2), it is suggested that two possible evolutionary pathways of poly(A) signals may exist: (a) UAA→UAAA→AAUAAA and (b) UAA→GUAA→UGUAA.

In terms of phylogenetic tree shown in Figure 2, the two diatoms and ciliates belong to the clade of simple organisms (species) in comparison with the other eight species. It is found that diatoms have a strong evolutionary relationship with bacteria in gene transfer, which has been a major driving force during their evolution [32]. So it is reasonable to assume that the dominant motif UAA (also a stop codon) detected in the two diatoms might represent the ancestral poly(A) signal, whereas UAAA and GUAA may represent the intermediate one between UAA and AAUAAA or between UAA and UGUAA.

The results of the single nucleotide variants from AAUAAA (Table 2) show that the UAAA motif is much conserved in AAUAAA group (Figure 3), and imply that the evolutionary pathway (a) might be true. Moreover, point mutations in AAUAAA signals in animal virus SV40 terminators strongly show that mutations in the last four positions (UAAA) cause much more reduction in cleavage and polyadenylation efficiency than those that occurred in first two positions (AA) [33]. Recently in *Trichomonas vaginalis*, a parasitic protozoon, UAAA was proven to be a core part of AAUAAA signal for polyadenylation, and its point mutation produced alternation of poly(A) sites [25,26]. In contrast, the mutation in AAUAAA signals in plants and yeast (*S. cerevisiae*) did not show a significant difference in polyadenylation efficiency in comparison with wild-types, and the 3′-half of the mutated motifs (-AAA) had slightly more tolerance than 5′-half (AAU-) in terms of mutation-induced polyadenylation efficiency changes [31]. This suggests that plants and yeast have stronger tolerance to point mutation of poly(A) signals in comparison to animals, which may partially explain the low frequency of AAUAAA in plants and yeast, considering that non-AAUAAA containing signals also could serve as polyadenylation signals with relatively high efficiency. Bioinformatic analysis revealed that the top 6 most significant hexamers in humans were AAUAAA and its variants (NNUAAA); their overall frequencies were up to 81.6% [34]. Moreover, the previous researches [25,26] also supported the idea that UAAA may be the ancestral motif in AAUAAA signal evolution. The signal pathway (a) UAA→UAAA→AAUAAA seems to be supported by both these previous studies and our results. In contrast, further evidence is needed to support the pathway (b) UAA→GUAA→UGUAA because our WebLogo result in Figure 3 suggests that only the first U in UGUAA is conserved, which conflicts with our assumption.

In spikemoss, moss and *Arabidopsis*, the frequency of the canonical AAUAAA is still low (<15%, Table 2) even combining with its single nucleotide variants. This is completely different from the UGUAA group and high-frequency AAUAAA group, in which the frequencies of single-nucleotide variants are 20%−25% and finally the overall combined frequencies (including both the core signal AAUAAA or UGUAA and corresponding mononucleotide variants) could be up to 60% or more. Other researchers also verified that there were high frequencies of AAUAAA (~50%) and its mononucleotide variants (~30%) in humans [11,12]. Therefore, it is suggested that plants and animals might have very different polyadenylation processes (including poly(A) signals and proteins) considering the huge differences in poly(A) signal frequencies.

It is noteworthy that no canonical poly(A) signal (i.e., AAUAAA or UGUAA) is detected in ciliates, diatoms or red alga, especially in ciliates where no conserved signals were found. There are still are RNAs with poly(A) tails detected in cDNA/mRNA sequencing in ciliates [35,36], which implies that polyadenylation is a normal mRNA processing event in this species. It is assumed that not only poly(A) signals but polyadenylation elements (e.g., FUE, NUE and CE) also play crucial roles in polyadenylation [2,37]. Although no significantly conserved poly(A) signal is detected in ciliates, the three elements (i.e., FUE, NUE and CE) are still clearly shown in its single nucleotide profile. Moreover, compared with SV40 (representing mammalian gene), CaMV (representing plants) has much higher tolerance to single nucleotide variation of poly(A) signals in polyadenylation efficiency detection, which means that non-canonical signals (e.g., AUUAAA, AAAAAA) still have higher polyadenylation efficiency in plants than in animals [31].

Clearly, these poly(A) signals do not function on their own, but in combination with an extensively characterized set of mRNA 3′ processing factors. Thus the functional unit that directs mRNA 3′ processing is composed of both RNAs and proteins. Furthermore, it is reported that the number of poly(A) protein factor coding genes distinctly increases in plant evolution from “lower” to “higher” [37]. The model of polyadenylation complex in plants is composed with a constant core part and numerous peripheral subunits, which may be explain the degenerated and diversified poly(A) signals in plants [37]. Such a model is helpful to understand the detected multiple signals (e.g., UAA, UAAA and AAUAAA) in the 11 investigated species. For example, it is possible that the three elements (not a single signal) around poly(A) sites are recognized and combined by the core poly(A) factors (e.g., cleavage and polyadenylation specificity factor) and some peripheral subunits in polyadenylation process in ciliates. However, how the variable poly(A) signals changed along with protein factors during evolution process in eukaryotes remains a mystery.

## 4. Material and Methods

### 4.1. Data Collection and Polyadenylation Site Definition

Taking advantage of sequenced genomes and relevant expressed sequence tags (ESTs) in GenBank dbEST and/or community databases, 11 eukaryotic species, including two diatoms *T. pseudonana* and *P. tricornutum*, ciliate *T. thermophila*, two green algae *C. reinhardtii* and *O. lucimarinus*, red alga *C. merolae*, spikemoss *S. moellendorffii*, moss *P. patens*, *Arabidopsis A. thaliana*, yeast *S. cerevisiae* and humans *H. sapiens* were selected to investigate the potential evolutionary patterns of poly(A) signals. In particular, the poly(A) signals of the seven species: diatoms *T. pseudonana* and *P. tricornutum*, ciliate *T. thermophila*, green alga *O. lucimarinus*, red alga *C. merolae*, spikemoss *S. moellendorffii* and moss *P. patens,* have not been reported previously. Because only EST data were available for these seven species whose poly(A) signals had not being studied previously, only EST-derived poly(A) data were utilized for the other four model organisms (*C. reinhardtii, A. thaliana*, yeast *S. cerevisiae* and human *H. sapiens*) to be comparative. When available, raw EST trace files were used to identify post-transcriptional poly(A) tails, consolidated by identification of cDNA termini to reduce false positives in poly(A) tail identification [38]. Detailed information about the collected EST data, genome sequences and available gene annotation is listed in Table S9. For example, nearly 77 k diatom *T. pseudonana* and 208 k diatom *P. tricornutum* EST sequences were obtained from the Diatom EST Database (http://www.diatomics.biologie.ens.fr/EST/) [39].

To determine the genomic poly(A) sites, all ESTs for the species other than yeast were mapped to their corresponding genomes using GMAP, a genomic mapping and alignment program for mRNA and EST sequences [40]. The poly(A) site data of yeast was directly downloaded from the website (http://harlequin.jax.org/polyA/) [41], which contained 1353 genomic sequences spanning 110 nt upstream and 50 nt downstream of the putative cleavage sites. The EST-to-genome mapping results were then analyzed and filtered for valid genomic hits using a similar protocol described previously [38]. Because poly(A) tails detected in ESTs are post-transcriptional, they should not be mapped into the genome except in the case where internal priming is likely to occur. Internal priming is defined as the case in which at least 6 consecutive adenines (As) are found or 7 As are detected from 10 nt-window in −10/+10 region around poly(A) sites in genomic sequences [12]. Therefore, genomic poly(A) sites were finally determined through the EST-to-genome mapping results, filtering out those that were potential internal priming candidates.

Frequently, multiple ESTs were mapped to the same poly(A) sites during EST-to-genome mapping. To eliminate the redundancy in the data, only one such poly(A) site was used to identify one unique poly(A) site. Finally using these non-redundant unique sites, 400 nt sequences were extracted from the corresponding genomes [i.e., 300 nt upstream and 100 nt downstream of the poly(A) sites, which was defined as −1 position [20]] for further data analysis.

The relative evolutionary positions of all these target species are showed in Figure 2 according to the Tree of Life web project (http://tolweb.org/tree/phylogeny.html).

### 4.2. Poly(A) Signal Elements Definition and Analysis

Based on previous research [2,7,12,14,15,16,20,41], the NUE region in our study was defined based on two criteria: (1) single nucleotide profile: NUE could start around the first crossing site of A and U (around −30) and end around another crossing site of A and U (around −10); (2) the significantly frequent and dominant motifs (if any) should exist in these regions. Once the NUE region was determined, FUE was defined as a range immediately upstream of NUE, in which a dominant single nucleotide profile could be evident. Based on our data, the start position of FUE was defined as the position where dominant G or U should appear. If not, −200 was used as the FUE start position. However, ciliate *T. thermophila* was an exception, because no dominant signal was found. Because we did not specifically examine the signals in CE, −10 and +10 were defined as the start and end positions of CE.

A new version of SignalSleuth [14], SignalSleuth2, was developed to perform exhaustive searches of short sequence motifs in a specified range of nucleotide sequences with variable motif sizes (generally 3−8 nt in length) and rank the detected motifs based on their occurrence frequencies. In addition, SignalSleuth2 has new functions including calculating Position-Specific Scoring Matrix (PSSM) scores and multiple scanning modes. Occasionally, a target motif may appear multiple times within a given region of a sequence. Such multiple occurrences might be overlapped, resulting in an over-representation of a specific motif. SignalSleuth2 provides a distance parameter (*-gap*) to prevent the over-counting of these overlapping motifs. This is what we call gap-scanning mode, which (*-gap = motif length-1*) is used to count non-overlapping signal frequency in a given region of a sequence. For example, ATATAT was counted only once in a sequence …ATATATAT… if *-gap* was set to be 5. For another example, if AATAAA motif was searched, *-gap* = 5 would avoid over counting the overlapping motifs. The motif frequencies reported in this study were obtained using the gap-scanning mode to avoid over counting of overlapping motifs. Meanwhile, SignalSleuth2 also allows users to use overlapping scanning mode (namely, *-gap = 0*) to obtain the frequencies of overlapping signals. Moreover, in the cases where there was more than one occurrence of nonoverlapping motifs in a given region, SignalSleuth2 can only choose the motif that is the closest to the poly(A) site. This is termed as once scanning mode. For each scanning mode, the SignalSleuth2 provides PSSM results simultaneously.

To evaluate the statistical significance of the signals, we used Z-Score to inspect the signals/motifs detected by regulatory sequence analysis tools (RSAT), which is based on a Markov chain model [27]. Considering the short length of triplets and tetramers, an order-1 Markov Model was used and the cutoff value for a valid Z-Score was set to 5; otherwise, an order-3 Markov Model was used and the cutoff value was 3. For the nucleotide composition in the CE region around poly(A) sites, WebLogo3.0 was used to examine the profiles of nucleotide composition [29].

## Figures and Tables

**Table 1:** The conserved poly(A) signals in the near upstream element (NUE) region of the seven species.

Species Name (Common Name)	Conserved Signal	Frequency (%)	Z-Score
*T. pseudonana* (T diatom)	UAA	86.51	19.12 ^1^
*P. tricornutum* (P diatom)	UAA	78.21	6.94 ^1^
*T. thermophila* (Ciliate)	-	-	-
*O. lucimarinus* (Ostreococcus)	UGUAA	30.4	30.21 ^1^
*C. merolae* (Red alga)	UAAA	86.45	15.33 ^1^
	GUAA	43.23	8.78 ^1^
*S. moellendorffii* (Spikemoss)	AAUAAA	7.83	7.18 ^3^
*P. patens* (Moss)	AAUAAA	7.25	15.46 ^3^

The superscript 1 in Z-score coloum means order-1 Markov model, the superscript 3 denotes order-3 Markov model.

**Table 2:** The frequencies of UGUAA and AAUAAA and their single nucleotide variants.

	Species Name (Common Name)	Canonical Signal (Frequency)	Variant (Frequency)	Overall Frequency
**UGUAA Group**	*C. reinhardtii* (Chlamydomonas)	UGUAA (50.38%)	UGCAA (6.42%) UGUUA (3.38%) UUUAA (3.28%) UGUAG (2.92%) UGUAU (2.43%) UGUGA (2.34%) UGAAA (2.33%) UGUAC (2.14%)	72.86%
	*O. lucimarinus* (Ostreococcus)	UGUAA (30.40%)	UUUAA (10.47%) UGAAA (5.75%) UGUAU (5.24%) UGUUA (4.60%) UGUGA (4.09%) UGCAA (4.09%)	59.64%
**AAUAAA Group**	*P. patens* (Moss)	AAUAAA (7.25%)	AGUAAA (2.73%) UAUAAA (2.25%)	11.93%
	*S. moellendorffii* (Spikemoss)	AAUAAA (7.83%)	UAUAAA (3.31%) AUUAAA (2.08%) AGUAAA (1.67%)	14.41%
	*A. thaliana (Arabidopsis)*	AAUAAA (8.59%)	UAUAAA (3.44%) AUUAAA (2.17%)	13.71%
	*H. sapiens* (Human)	AAUAAA (64.92%)	AUUAAA (16.68%) UAUAAA (4.30%) AGUAAA (3.76%)	85.19%

## Supplementary Materials

Supplementary materials can be found at https://www.mdpi.com/1422-0067/20/4/958/s1. Figure S1. The distributions of 50 top-ranked poly(A) signals in the NUE regions of the seven previously unstudied species. Figure S2. The distribution of 50 top-ranked poly(A) signals in the FUE regions in the seven previously unstudied species. Figure S3. The nucleotides composition in CE region in the seven unstudied species. Figure S4. The conserved structures in CE region in the seven species. Table S1. The percentages of four nucleotides in seven previously unstudied species. Table S2. The positional frequency profile of top 50 motifs (3 nt–8 nt) in the FUE region in T diatom (*T. pseudonana*). Table S3. The positional frequency profile of top 50 motifs (3 nt–8 nt) in the FUE region in P diatom (*P. tricornutum*). Table S4. The positional frequency profile of top 50 motifs (3 nt–8 nt) in the FUE region in Ciliates (*T. thermophila*). Table S5. The positional frequency profile of top 50 motifs (3 nt–8 nt) in the FUE region in Ostreococcus (*O. lucimarinus*). Table S6. The positional frequency profile of top 50 motifs (3 nt–8 nt) in the FUE region in red alga (*C. merolae*). Table S7. The positional frequency profile of top 50 motifs (3 nt–8 nt) in the FUE region in spikemoss (*S. moellendorffii*). Table S8. The positional frequency profile of top 50 motifs (3 nt–8 nt) in the FUE region in moss (*P. patens*). Table S9. The data sources of the 11 species for poly(A) signal analysis.

## Abbreviations

B	nucleotides U, C, or G
CE	cleavage element
EST	Expressed sequence tag
FUE	far upstream element
Nt	nucleotide
NUE	near upstream element
Y	nucleotides U or C
