# Supplementary material for: Genome-Wide Comparative Analyses of Polyadenylation Signals in Eukaryotes Suggest a Possible Origin of the AAUAAA Signal
Source: Int J Mol Sci. 2019 Feb 22;20(4):958. doi: 10.3390/ijms20040958 (PMC6413133; doi:10.3390/ijms20040958)
Supplement: Supplementary file 1 [file ijms-20-00958-s001.zip › ijms-444287 suppl final/Appendix Figures and Tables-revised/Figure S1.pptx]

## Slide 1
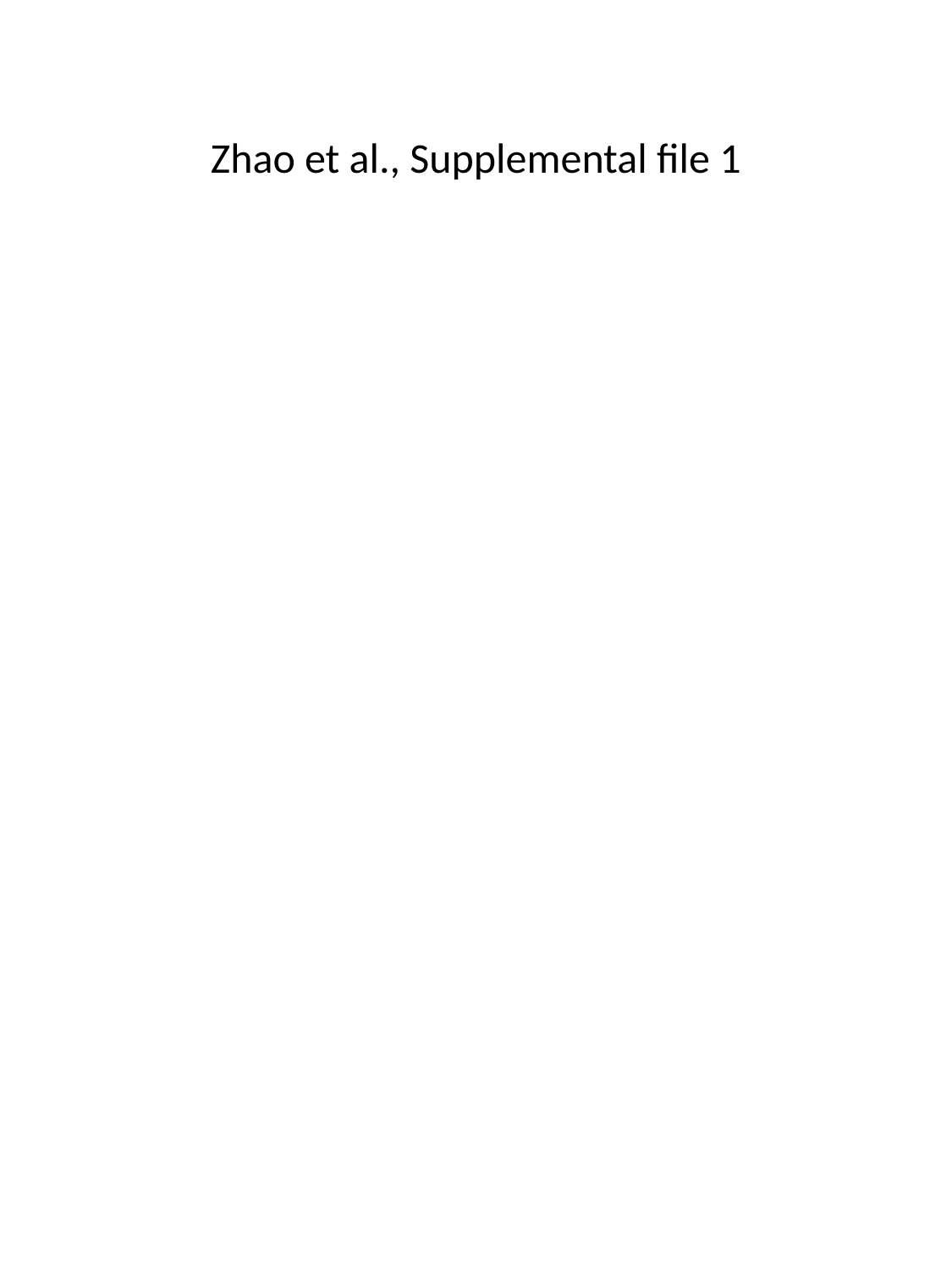

# Zhao et al., Supplemental file 1

## Slide 2
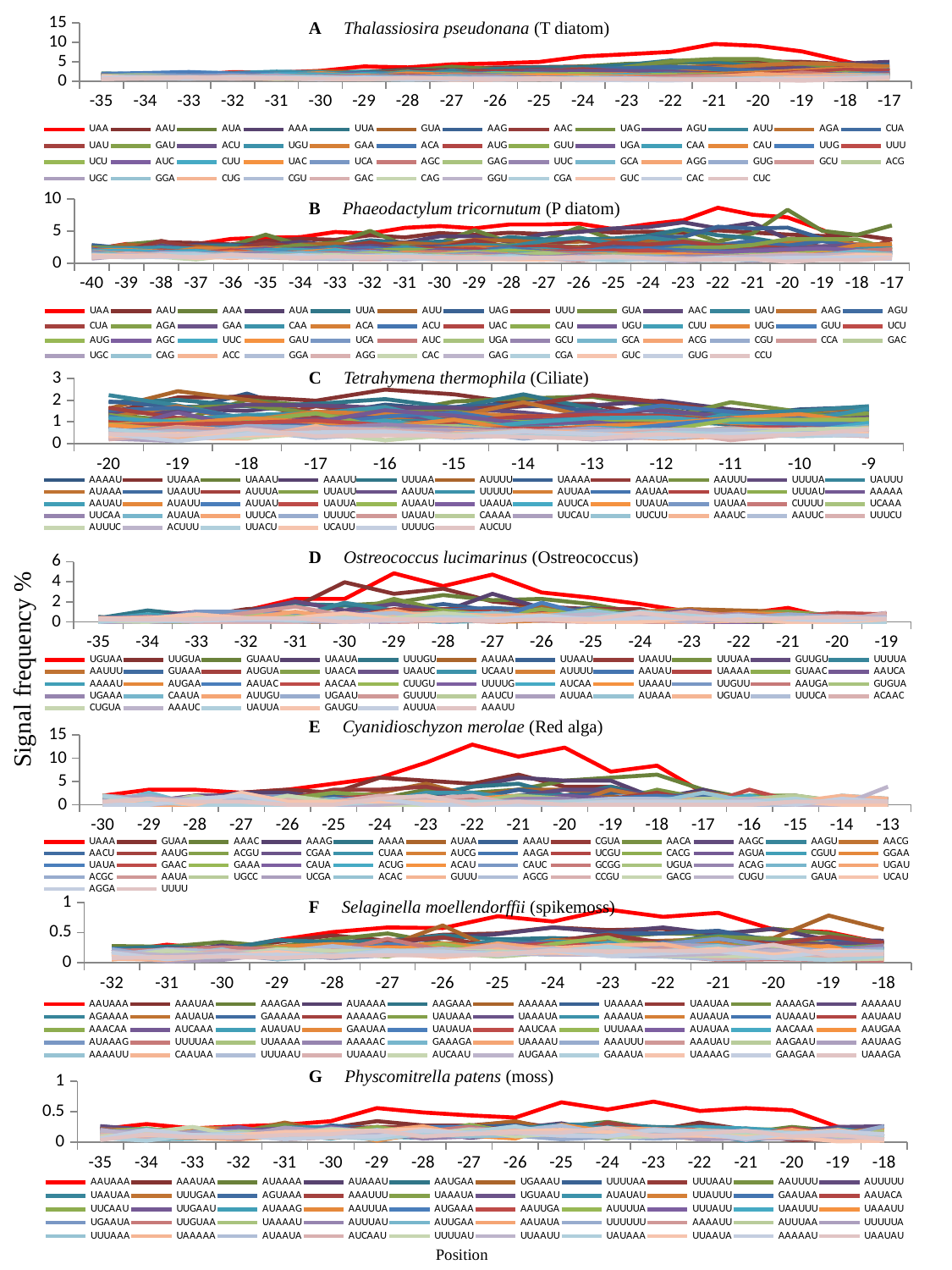

A Thalassiosira pseudonana (T diatom)
### Chart
| Category | UAA | AAU | AUA | AAA | UUA | GUA | AAG | AAC | UAG | AGU | AUU | AGA | CUA | UAU | GAU | ACU | UGU | GAA | ACA | AUG | GUU | UGA | CAA | CAU | UUG | UUU | UCU | AUC | CUU | UAC | UCA | AGC | GAG | UUC | GCA | AGG | GUG | GCU | ACG | UGC | GGA | CUG | CGU | GAC | CAG | GGU | CGA | GUC | CAC | CUC |
|---|---|---|---|---|---|---|---|---|---|---|---|---|---|---|---|---|---|---|---|---|---|---|---|---|---|---|---|---|---|---|---|---|---|---|---|---|---|---|---|---|---|---|---|---|---|---|---|---|---|---|
| -35 | 0.985389058783554 | 1.3931362555215758 | 1.5290519877675839 | 1.1213047910295517 | 1.1213047910295517 | 1.6649677200136013 | 0.8834522595990489 | 1.0533469249065754 | 1.563030920829086 | 1.4610941216445799 | 1.2911994563370597 | 1.6649677200136013 | 0.713557594291539 | 1.1213047910295517 | 1.936799184505606 | 1.0533469249065754 | 1.936799184505606 | 1.5290519877675839 | 1.3251783893985731 | 1.597009853890588 | 1.8688413183826018 | 1.698946653075093 | 1.63098878695209 | 1.1552837240910763 | 1.8688413183826018 | 1.495073054706082 | 1.019367991845056 | 1.1552837240910763 | 1.1552837240910763 | 1.2911994563370597 | 1.3251783893985731 | 0.781515460414543 | 1.427115188583078 | 1.019367991845056 | 1.25722052327557 | 0.985389058783554 | 1.25722052327557 | 0.815494393476045 | 1.019367991845056 | 0.747536527353041 | 1.25722052327557 | 0.5776418620455422 | 1.019367991845056 | 0.985389058783554 | 0.6455997281685422 | 0.9174311926605511 | 0.9174311926605511 | 1.2232415902140548 | 0.9174311926605511 | 0.5096839959225279 |
| -34 | 0.9514101257220418 | 1.597009853890588 | 1.3591573224600761 | 1.0873258579680598 | 1.3931362555215758 | 1.970778117567107 | 1.2911994563370597 | 1.1552837240910763 | 1.427115188583078 | 1.6649677200136013 | 1.597009853890588 | 1.2911994563370597 | 0.781515460414543 | 1.563030920829086 | 1.732925586136597 | 1.0873258579680598 | 1.732925586136597 | 1.6649677200136013 | 1.8688413183826018 | 1.2911994563370597 | 1.5290519877675839 | 1.597009853890588 | 1.3931362555215758 | 1.6649677200136013 | 2.072714916751614 | 1.2911994563370597 | 1.0533469249065754 | 1.427115188583078 | 1.1213047910295517 | 1.3251783893985731 | 1.6649677200136013 | 0.747536527353041 | 1.495073054706082 | 0.985389058783554 | 1.1552837240910763 | 0.6795786612300381 | 1.2232415902140548 | 1.019367991845056 | 0.815494393476045 | 1.019367991845056 | 0.713557594291539 | 0.9174311926605511 | 0.6795786612300381 | 0.9174311926605511 | 0.985389058783554 | 0.713557594291539 | 0.713557594291539 | 0.849473326537547 | 0.849473326537547 | 0.713557594291539 |
| -33 | 1.5290519877675839 | 1.3931362555215758 | 1.732925586136597 | 1.1213047910295517 | 1.3591573224600761 | 1.63098878695209 | 1.3591573224600761 | 1.3931362555215758 | 1.0873258579680598 | 1.732925586136597 | 1.6649677200136013 | 2.072714916751614 | 1.0533469249065754 | 1.563030920829086 | 1.5290519877675839 | 1.2911994563370597 | 2.208630648997664 | 1.4610941216445799 | 1.698946653075093 | 1.563030920829086 | 1.597009853890588 | 1.597009853890588 | 1.8688413183826018 | 1.698946653075093 | 2.3445463812436267 | 1.4610941216445799 | 1.2911994563370597 | 1.1892626571525557 | 0.849473326537547 | 1.3591573224600761 | 1.4610941216445799 | 1.2232415902140548 | 0.9514101257220418 | 1.019367991845056 | 1.2232415902140548 | 0.9514101257220418 | 1.0873258579680598 | 0.9514101257220418 | 0.8834522595990489 | 1.0533469249065754 | 0.713557594291539 | 0.747536527353041 | 0.6795786612300381 | 0.9174311926605511 | 0.849473326537547 | 0.781515460414543 | 0.6795786612300381 | 0.6455997281685422 | 0.713557594291539 | 0.985389058783554 |
| -32 | 2.3105674481821272 | 1.3591573224600761 | 1.3251783893985731 | 1.6649677200136013 | 1.8008834522595978 | 2.14067278287462 | 1.3591573224600761 | 1.495073054706082 | 1.019367991845056 | 1.3251783893985731 | 1.936799184505606 | 1.495073054706082 | 1.0533469249065754 | 1.427115188583078 | 1.834862385321101 | 1.427115188583078 | 2.072714916751614 | 1.495073054706082 | 1.63098878695209 | 1.9028202514440928 | 1.63098878695209 | 1.6649677200136013 | 1.2232415902140548 | 1.63098878695209 | 1.9028202514440928 | 1.3251783893985731 | 1.2232415902140548 | 1.0533469249065754 | 1.2232415902140548 | 1.0533469249065754 | 1.63098878695209 | 0.985389058783554 | 0.9514101257220418 | 0.9514101257220418 | 0.6455997281685422 | 0.815494393476045 | 1.1213047910295517 | 1.2232415902140548 | 0.985389058783554 | 0.985389058783554 | 0.815494393476045 | 1.1213047910295517 | 0.6795786612300381 | 0.6455997281685422 | 0.781515460414543 | 0.985389058783554 | 0.8834522595990489 | 0.8834522595990489 | 0.985389058783554 | 0.6455997281685422 |
| -31 | 2.208630648997664 | 2.1066938498131162 | 1.732925586136597 | 1.4610941216445799 | 2.004757050628611 | 1.970778117567107 | 1.5290519877675839 | 2.072714916751614 | 0.781515460414543 | 1.5290519877675839 | 1.970778117567107 | 1.3931362555215758 | 1.25722052327557 | 1.4610941216445799 | 1.5290519877675839 | 1.5290519877675839 | 2.4464831804281126 | 1.9028202514440928 | 1.63098878695209 | 1.732925586136597 | 1.8008834522595978 | 1.732925586136597 | 1.2911994563370597 | 1.495073054706082 | 1.9028202514440928 | 1.698946653075093 | 1.3591573224600761 | 1.1213047910295517 | 1.7669045191980979 | 1.2911994563370597 | 1.2232415902140548 | 0.781515460414543 | 0.8834522595990489 | 0.9514101257220418 | 1.4610941216445799 | 0.5436629289840299 | 1.0873258579680598 | 0.713557594291539 | 0.5436629289840299 | 1.495073054706082 | 0.6455997281685422 | 0.985389058783554 | 0.8834522595990489 | 0.6455997281685422 | 0.6795786612300381 | 0.849473326537547 | 0.985389058783554 | 0.747536527353041 | 0.815494393476045 | 0.815494393476045 |
| -30 | 2.616377845735644 | 1.8008834522595978 | 2.072714916751614 | 1.63098878695209 | 2.3445463812436267 | 2.54841997961264 | 1.8688413183826018 | 1.698946653075093 | 0.985389058783554 | 1.6649677200136013 | 1.7669045191980979 | 1.1552837240910763 | 1.3591573224600761 | 1.3251783893985731 | 1.8688413183826018 | 1.8008834522595978 | 2.2765885151206247 | 1.25722052327557 | 1.698946653075093 | 1.732925586136597 | 1.732925586136597 | 1.563030920829086 | 1.1892626571525557 | 2.072714916751614 | 1.6649677200136013 | 1.3251783893985731 | 1.4610941216445799 | 1.2232415902140548 | 1.698946653075093 | 1.2232415902140548 | 1.25722052327557 | 0.5436629289840299 | 1.1552837240910763 | 1.2232415902140548 | 0.9514101257220418 | 0.713557594291539 | 1.019367991845056 | 0.9174311926605511 | 0.713557594291539 | 0.713557594291539 | 0.611620795107034 | 0.781515460414543 | 0.441726129799525 | 1.0533469249065754 | 0.9174311926605511 | 0.815494393476045 | 0.985389058783554 | 0.441726129799525 | 0.475705062861026 | 0.611620795107034 |
| -29 | 3.771661569826707 | 2.3445463812436267 | 2.208630648997664 | 1.732925586136597 | 2.3445463812436267 | 2.208630648997664 | 1.698946653075093 | 2.038735983690112 | 1.0533469249065754 | 1.7669045191980979 | 2.4464831804281126 | 1.563030920829086 | 2.038735983690112 | 1.0873258579680598 | 1.3931362555215758 | 1.597009853890588 | 1.427115188583078 | 1.495073054706082 | 2.004757050628611 | 1.6649677200136013 | 1.6649677200136013 | 1.970778117567107 | 1.834862385321101 | 1.1892626571525557 | 1.597009853890588 | 1.2232415902140548 | 1.495073054706082 | 0.781515460414543 | 1.597009853890588 | 1.3931362555215758 | 1.0873258579680598 | 0.9514101257220418 | 1.0873258579680598 | 1.1552837240910763 | 0.611620795107034 | 0.713557594291539 | 0.8834522595990489 | 0.5096839959225279 | 0.40774719673802196 | 0.6795786612300381 | 1.019367991845056 | 0.5776418620455422 | 0.849473326537547 | 0.6795786612300381 | 0.5776418620455422 | 0.5776418620455422 | 0.5776418620455422 | 0.5436629289840299 | 0.8834522595990489 | 0.475705062861026 |
| -28 | 3.533809038396194 | 3.1260618416581742 | 2.6843357118586684 | 2.6503567787971707 | 2.4804621134896063 | 2.14067278287462 | 1.8688413183826018 | 2.412504247366641 | 0.815494393476045 | 1.8008834522595978 | 1.63098878695209 | 1.4610941216445799 | 1.63098878695209 | 2.3445463812436267 | 2.412504247366641 | 1.970778117567107 | 2.242609582059123 | 1.834862385321101 | 1.732925586136597 | 1.597009853890588 | 1.427115188583078 | 1.3251783893985731 | 1.6649677200136013 | 2.004757050628611 | 1.4610941216445799 | 1.4610941216445799 | 1.1213047910295517 | 1.1552837240910763 | 1.0533469249065754 | 1.1213047910295517 | 0.985389058783554 | 0.849473326537547 | 0.8834522595990489 | 1.25722052327557 | 0.9514101257220418 | 0.40774719673802196 | 0.8834522595990489 | 0.5436629289840299 | 0.713557594291539 | 1.0873258579680598 | 0.849473326537547 | 0.6795786612300381 | 0.5436629289840299 | 0.5776418620455422 | 0.5436629289840299 | 0.713557594291539 | 0.3058103975535201 | 0.6795786612300381 | 0.441726129799525 | 0.815494393476045 |
| -27 | 4.315324498810737 | 3.0581039755351678 | 3.703703703703704 | 2.9221882432891597 | 2.242609582059123 | 2.8542303771661572 | 2.3105674481821272 | 2.7183146449201505 | 0.849473326537547 | 1.8008834522595978 | 2.7183146449201505 | 1.2232415902140548 | 1.698946653075093 | 2.072714916751614 | 1.597009853890588 | 2.038735983690112 | 2.1066938498131162 | 1.3591573224600761 | 1.3931362555215758 | 1.698946653075093 | 1.9028202514440928 | 1.597009853890588 | 1.4610941216445799 | 1.63098878695209 | 1.2232415902140548 | 1.4610941216445799 | 1.563030920829086 | 1.732925586136597 | 1.1892626571525557 | 1.563030920829086 | 1.3251783893985731 | 0.611620795107034 | 0.6795786612300381 | 0.985389058783554 | 1.0873258579680598 | 0.475705062861026 | 0.5776418620455422 | 0.781515460414543 | 0.6455997281685422 | 0.611620795107034 | 0.271831464492015 | 0.781515460414543 | 0.781515460414543 | 0.33978933061501904 | 0.475705062861026 | 0.5096839959225279 | 0.611620795107034 | 0.33978933061501904 | 0.441726129799525 | 0.6795786612300381 |
| -26 | 4.5531770302412475 | 3.4998301053346927 | 3.1260618416581742 | 2.4464831804281126 | 3.1260618416581742 | 2.4804621134896063 | 2.4804621134896063 | 2.514441046551138 | 1.1552837240910763 | 2.004757050628611 | 1.936799184505606 | 1.3591573224600761 | 2.4804621134896063 | 2.4804621134896063 | 1.6649677200136013 | 2.072714916751614 | 1.732925586136597 | 1.427115188583078 | 1.834862385321101 | 1.495073054706082 | 1.698946653075093 | 1.2232415902140548 | 2.072714916751614 | 1.563030920829086 | 1.698946653075093 | 1.25722052327557 | 1.8688413183826018 | 1.597009853890588 | 1.563030920829086 | 1.3931362555215758 | 1.25722052327557 | 0.5776418620455422 | 0.475705062861026 | 1.0873258579680598 | 0.611620795107034 | 0.5096839959225279 | 0.815494393476045 | 0.781515460414543 | 0.5436629289840299 | 0.815494393476045 | 0.271831464492015 | 0.849473326537547 | 0.849473326537547 | 0.271831464492015 | 0.5096839959225279 | 0.611620795107034 | 0.33978933061501904 | 0.40774719673802196 | 0.6455997281685422 | 0.441726129799525 |
| -25 | 4.926945293917771 | 3.601766904519198 | 3.227998640842707 | 3.160040774719674 | 3.4998301053346927 | 2.8202514441046547 | 2.2765885151206247 | 2.9561671763506627 | 1.495073054706082 | 2.6503567787971707 | 2.54841997961264 | 1.3931362555215758 | 2.7183146449201505 | 2.412504247366641 | 1.3591573224600761 | 2.3105674481821272 | 2.004757050628611 | 1.2232415902140548 | 1.63098878695209 | 1.6649677200136013 | 1.4610941216445799 | 1.970778117567107 | 1.7669045191980979 | 1.2911994563370597 | 0.747536527353041 | 1.427115188583078 | 1.7669045191980979 | 1.3931362555215758 | 1.3931362555215758 | 1.495073054706082 | 0.9514101257220418 | 0.747536527353041 | 0.40774719673802196 | 0.9174311926605511 | 0.713557594291539 | 0.475705062861026 | 0.747536527353041 | 0.815494393476045 | 0.5436629289840299 | 0.611620795107034 | 0.5776418620455422 | 0.6795786612300381 | 0.5436629289840299 | 0.40774719673802196 | 0.6795786612300381 | 0.5436629289840299 | 0.40774719673802196 | 0.781515460414543 | 0.5096839959225279 | 0.441726129799525 |
| -24 | 6.35406048250085 | 3.771661569826707 | 3.7376826367652027 | 3.6357458375806977 | 3.5677879714577236 | 3.1260618416581742 | 2.8202514441046547 | 2.9221882432891597 | 1.698946653075093 | 2.208630648997664 | 3.0581039755351678 | 1.63098878695209 | 3.0581039755351678 | 3.0581039755351678 | 1.970778117567107 | 2.6503567787971707 | 1.563030920829086 | 1.563030920829086 | 1.563030920829086 | 1.1892626571525557 | 1.563030920829086 | 1.563030920829086 | 1.25722052327557 | 1.7669045191980979 | 1.1213047910295517 | 1.1552837240910763 | 1.8008834522595978 | 0.985389058783554 | 1.2232415902140548 | 1.2911994563370597 | 0.9514101257220418 | 0.713557594291539 | 0.6455997281685422 | 0.849473326537547 | 0.611620795107034 | 0.5776418620455422 | 0.815494393476045 | 0.747536527353041 | 0.9514101257220418 | 0.37376826367652105 | 0.271831464492015 | 0.37376826367652105 | 0.781515460414543 | 0.20387359836901098 | 0.475705062861026 | 0.475705062861026 | 0.3058103975535201 | 0.441726129799525 | 0.33978933061501904 | 0.611620795107034 |
| -23 | 6.897723411484892 | 3.975535168195718 | 4.451240231056747 | 3.5677879714577236 | 4.145429833503228 | 3.0581039755351678 | 2.6843357118586684 | 2.9561671763506627 | 3.6357458375806977 | 2.75229357798166 | 3.295956506965681 | 1.597009853890588 | 3.295956506965681 | 2.4804621134896063 | 1.597009853890588 | 2.582398912674142 | 1.63098878695209 | 1.1892626571525557 | 1.563030920829086 | 1.8008834522595978 | 1.2232415902140548 | 1.3591573224600761 | 1.1552837240910763 | 1.732925586136597 | 0.9514101257220418 | 1.3591573224600761 | 1.4610941216445799 | 1.25722052327557 | 1.597009853890588 | 1.2232415902140548 | 0.9174311926605511 | 0.9174311926605511 | 0.5436629289840299 | 0.6455997281685422 | 0.441726129799525 | 0.781515460414543 | 0.6795786612300381 | 0.6455997281685422 | 0.713557594291539 | 0.40774719673802196 | 0.40774719673802196 | 0.5436629289840299 | 0.815494393476045 | 0.271831464492015 | 0.5096839959225279 | 0.5776418620455422 | 0.5436629289840299 | 0.237852531430513 | 0.237852531430513 | 0.5436629289840299 |
| -22 | 7.509344206591908 | 4.960924226979211 | 4.621134896364194 | 3.4658511722731777 | 5.300713557594284 | 3.295956506965681 | 3.1260618416581742 | 1.970778117567107 | 5.130818892286777 | 3.0241250424736705 | 2.75229357798166 | 2.17465171593612 | 3.703703703703704 | 2.14067278287462 | 1.597009853890588 | 2.514441046551138 | 1.936799184505606 | 1.6649677200136013 | 1.7669045191980979 | 1.1892626571525557 | 1.597009853890588 | 1.427115188583078 | 1.1892626571525557 | 1.3251783893985731 | 0.713557594291539 | 1.698946653075093 | 1.6649677200136013 | 1.1213047910295517 | 0.985389058783554 | 1.1213047910295517 | 0.713557594291539 | 1.2911994563370597 | 0.713557594291539 | 0.5096839959225279 | 0.37376826367652105 | 0.713557594291539 | 0.713557594291539 | 0.815494393476045 | 0.40774719673802196 | 0.5776418620455422 | 0.33978933061501904 | 0.40774719673802196 | 0.475705062861026 | 0.40774719673802196 | 0.5776418620455422 | 0.475705062861026 | 0.37376826367652105 | 0.37376826367652105 | 0.169894665307509 | 0.37376826367652105 |
| -21 | 9.548080190282018 | 4.655113829425671 | 4.858987427794767 | 3.771661569826707 | 4.757050628610234 | 3.975535168195718 | 3.0581039755351678 | 2.8542303771661572 | 5.674481821270759 | 3.5677879714577236 | 2.786272511043153 | 3.329935440027183 | 3.227998640842707 | 2.038735983690112 | 1.63098878695209 | 1.732925586136597 | 1.427115188583078 | 1.834862385321101 | 1.3251783893985731 | 1.563030920829086 | 1.597009853890588 | 0.9514101257220418 | 1.1552837240910763 | 1.1552837240910763 | 0.747536527353041 | 1.019367991845056 | 1.0533469249065754 | 1.0873258579680598 | 1.1213047910295517 | 0.849473326537547 | 0.781515460414543 | 1.3591573224600761 | 1.0533469249065754 | 0.713557594291539 | 1.019367991845056 | 1.1213047910295517 | 0.3058103975535201 | 1.1213047910295517 | 0.611620795107034 | 0.33978933061501904 | 0.441726129799525 | 0.5096839959225279 | 0.3058103975535201 | 0.441726129799525 | 0.40774719673802196 | 0.40774719673802196 | 0.441726129799525 | 0.33978933061501904 | 0.441726129799525 | 0.3058103975535201 |
| -20 | 9.072375127421 | 5.0628610261637785 | 4.315324498810737 | 4.077471967380224 | 3.703703703703704 | 3.26197757390418 | 4.145429833503228 | 2.75229357798166 | 5.674481821270759 | 2.9561671763506627 | 1.7669045191980979 | 4.145429833503228 | 2.54841997961264 | 1.563030920829086 | 1.6649677200136013 | 1.8688413183826018 | 1.3251783893985731 | 2.208630648997664 | 1.732925586136597 | 1.834862385321101 | 1.1892626571525557 | 1.3931362555215758 | 1.4610941216445799 | 0.8834522595990489 | 0.9514101257220418 | 1.427115188583078 | 0.849473326537547 | 1.5290519877675839 | 1.1213047910295517 | 0.9174311926605511 | 1.1552837240910763 | 1.25722052327557 | 1.0533469249065754 | 0.5096839959225279 | 0.6795786612300381 | 1.834862385321101 | 0.747536527353041 | 0.611620795107034 | 0.6795786612300381 | 0.3058103975535201 | 0.6795786612300381 | 0.441726129799525 | 0.40774719673802196 | 0.611620795107034 | 0.5776418620455422 | 0.611620795107034 | 0.6795786612300381 | 0.5436629289840299 | 0.6455997281685422 | 0.3058103975535201 |
| -19 | 7.64525993883792 | 4.960924226979211 | 3.601766904519198 | 4.349303431872253 | 2.8202514441046547 | 2.3445463812436267 | 3.8056405028881977 | 4.145429833503228 | 4.213387699626232 | 3.4318722392116556 | 1.834862385321101 | 4.587155963302735 | 2.038735983690112 | 1.7669045191980979 | 2.4804621134896063 | 1.5290519877675839 | 1.2232415902140548 | 2.786272511043153 | 2.004757050628611 | 2.3445463812436267 | 1.3931362555215758 | 1.698946653075093 | 1.4610941216445799 | 1.3591573224600761 | 1.3591573224600761 | 0.611620795107034 | 0.9174311926605511 | 1.563030920829086 | 1.1892626571525557 | 0.9514101257220418 | 1.2232415902140548 | 2.208630648997664 | 0.985389058783554 | 0.6795786612300381 | 0.713557594291539 | 1.4610941216445799 | 0.815494393476045 | 0.6795786612300381 | 1.0873258579680598 | 0.713557594291539 | 1.0533469249065754 | 0.475705062861026 | 0.5436629289840299 | 1.0533469249065754 | 0.611620795107034 | 0.611620795107034 | 0.5096839959225279 | 0.815494393476045 | 0.3058103975535201 | 0.101936799184506 |
| -18 | 5.0628610261637785 | 4.383282364933741 | 3.4658511722731777 | 4.519198097179749 | 1.698946653075093 | 2.072714916751614 | 3.6697247706422407 | 3.533809038396194 | 2.9901461094121577 | 2.3105674481821272 | 2.75229357798166 | 4.315324498810737 | 1.2232415902140548 | 1.6649677200136013 | 2.2765885151206247 | 2.17465171593612 | 1.732925586136597 | 3.26197757390418 | 2.242609582059123 | 2.9221882432891597 | 2.17465171593612 | 1.8688413183826018 | 1.8008834522595978 | 1.2911994563370597 | 1.2232415902140548 | 1.0873258579680598 | 0.815494393476045 | 1.63098878695209 | 1.0533469249065754 | 1.2232415902140548 | 1.4610941216445799 | 1.3591573224600761 | 1.427115188583078 | 1.0533469249065754 | 1.563030920829086 | 1.698946653075093 | 0.9514101257220418 | 0.8834522595990489 | 1.427115188583078 | 0.9174311926605511 | 1.3591573224600761 | 0.815494393476045 | 0.475705062861026 | 1.2232415902140548 | 0.6795786612300381 | 0.441726129799525 | 0.815494393476045 | 0.6455997281685422 | 0.6455997281685422 | 0.441726129799525 |
| -17 | 2.7183146449201505 | 3.7376826367652027 | 3.0581039755351678 | 4.960924226979211 | 2.038735983690112 | 1.7669045191980979 | 4.213387699626232 | 3.295956506965681 | 1.970778117567107 | 2.3445463812436267 | 2.14067278287462 | 3.7376826367652027 | 1.3251783893985731 | 2.242609582059123 | 3.0581039755351678 | 1.563030920829086 | 1.563030920829086 | 2.9901461094121577 | 2.8542303771661572 | 2.6843357118586684 | 1.63098878695209 | 2.1066938498131162 | 1.8688413183826018 | 1.732925586136597 | 1.970778117567107 | 1.834862385321101 | 1.495073054706082 | 1.732925586136597 | 1.1213047910295517 | 1.5290519877675839 | 1.495073054706082 | 1.3251783893985731 | 1.3251783893985731 | 1.4610941216445799 | 1.25722052327557 | 1.3591573224600761 | 0.9174311926605511 | 1.019367991845056 | 1.495073054706082 | 1.3591573224600761 | 1.2911994563370597 | 0.8834522595990489 | 1.019367991845056 | 0.985389058783554 | 0.985389058783554 | 0.713557594291539 | 0.985389058783554 | 0.6455997281685422 | 0.985389058783554 | 0.9514101257220418 |
### Chart
| Category | UAA | AAU | AAA | AUA | UUA | AUU | UAG | UUU | GUA | AAC | UAU | AAG | AGU | CUA | AGA | GAA | CAA | ACA | ACU | UAC | CAU | UGU | CUU | UUG | GUU | UCU | AUG | AGC | UUC | GAU | UCA | AUC | UGA | GCU | GCA | ACG | CGU | CCA | GAC | UGC | CAG | ACC | GGA | AGG | CAC | GAG | CGA | GUC | GUG | CCU |
|---|---|---|---|---|---|---|---|---|---|---|---|---|---|---|---|---|---|---|---|---|---|---|---|---|---|---|---|---|---|---|---|---|---|---|---|---|---|---|---|---|---|---|---|---|---|---|---|---|---|---|
| -40 | 2.13068181818182 | 2.6988636363636327 | 2.102272727272727 | 2.3295454545454537 | 2.102272727272727 | 2.244318181818182 | 2.8124999999999716 | 2.414772727272727 | 2.4715909090909087 | 1.3920454545454561 | 1.6477272727272718 | 2.3295454545454537 | 2.102272727272727 | 1.789772727272727 | 2.1875000000000244 | 1.875 | 2.1875000000000244 | 2.0738636363636327 | 1.1931818181818181 | 2.215909090909091 | 1.8181818181818181 | 1.5625 | 2.13068181818182 | 1.960227272727272 | 2.0738636363636327 | 1.53409090909091 | 1.7613636363636358 | 1.6761363636363742 | 1.875 | 1.84659090909091 | 1.7613636363636358 | 1.505681818181818 | 1.5625 | 1.5909090909090808 | 1.420454545454545 | 1.2784090909090808 | 1.363636363636363 | 1.0227272727272718 | 1.079545454545454 | 0.710227272727273 | 1.306818181818182 | 1.0227272727272718 | 0.9943181818181769 | 0.795454545454545 | 1.107954545454545 | 1.079545454545454 | 1.2784090909090808 | 1.1931818181818181 | 0.9375 | 0.909090909090909 |
| -39 | 2.982954545454545 | 2.357954545454545 | 2.926136363636364 | 2.556818181818139 | 2.5 | 2.840909090909091 | 2.301136363636364 | 2.0170454545454537 | 2.357954545454545 | 1.5625 | 1.960227272727272 | 1.789772727272727 | 2.215909090909091 | 1.789772727272727 | 2.045454545454545 | 2.357954545454545 | 2.3295454545454537 | 2.045454545454545 | 1.704545454545455 | 1.6477272727272718 | 1.931818181818182 | 1.9886363636363742 | 1.3920454545454561 | 2.045454545454545 | 1.5909090909090808 | 1.53409090909091 | 1.306818181818182 | 1.789772727272727 | 1.931818181818182 | 1.477272727272727 | 1.505681818181818 | 1.53409090909091 | 1.3920454545454561 | 1.1647727272727373 | 1.363636363636363 | 1.051136363636364 | 0.909090909090909 | 1.1931818181818181 | 1.136363636363636 | 1.1931818181818181 | 1.1931818181818181 | 1.079545454545454 | 0.9943181818181769 | 1.505681818181818 | 0.909090909090909 | 1.306818181818182 | 1.107954545454545 | 1.136363636363636 | 0.9375 | 0.9943181818181769 |
| -38 | 2.9545454545454537 | 3.267045454545454 | 3.4090909090909087 | 2.272727272727301 | 2.0170454545454537 | 2.6988636363636327 | 2.4431818181818454 | 3.465909090909091 | 2.102272727272727 | 2.75568181818182 | 1.8181818181818181 | 2.1590909090909087 | 1.84659090909091 | 1.875 | 2.244318181818182 | 2.0170454545454537 | 2.414772727272727 | 1.505681818181818 | 1.619318181818182 | 1.9886363636363742 | 1.704545454545455 | 1.6761363636363742 | 1.7329545454545459 | 1.8181818181818181 | 1.7329545454545459 | 1.8181818181818181 | 1.3352272727272718 | 1.25 | 1.7329545454545459 | 1.420454545454545 | 1.619318181818182 | 1.420454545454545 | 1.4488636363636358 | 1.704545454545455 | 1.477272727272727 | 1.2215909090909078 | 1.0227272727272718 | 1.306818181818182 | 1.051136363636364 | 1.1931818181818181 | 1.0227272727272718 | 0.909090909090909 | 1.505681818181818 | 1.25 | 0.9659090909090972 | 1.051136363636364 | 1.1931818181818181 | 1.1931818181818181 | 0.9375 | 1.107954545454545 |
| -37 | 2.9545454545454537 | 3.153409090909091 | 2.357954545454545 | 2.8693181818181777 | 2.585227272727301 | 2.585227272727301 | 1.8181818181818181 | 2.357954545454545 | 2.1875000000000244 | 2.0738636363636327 | 1.5909090909090808 | 1.9886363636363742 | 2.0170454545454537 | 1.7613636363636358 | 1.960227272727272 | 2.215909090909091 | 2.272727272727301 | 2.301136363636364 | 1.931818181818182 | 1.704545454545455 | 1.789772727272727 | 1.6761363636363742 | 1.84659090909091 | 1.7329545454545459 | 1.5625 | 1.53409090909091 | 1.25 | 1.53409090909091 | 1.875 | 1.306818181818182 | 1.53409090909091 | 1.2784090909090808 | 1.2215909090909078 | 1.306818181818182 | 1.306818181818182 | 1.1931818181818181 | 1.2215909090909078 | 1.051136363636364 | 1.420454545454545 | 1.2784090909090808 | 1.25 | 1.1647727272727373 | 1.477272727272727 | 0.9943181818181769 | 0.5681818181818231 | 1.3920454545454561 | 1.3352272727272718 | 0.795454545454545 | 1.0227272727272718 | 0.909090909090909 |
| -36 | 3.77840909090909 | 2.926136363636364 | 2.7272727272727506 | 2.840909090909091 | 2.7840909090909105 | 2.244318181818182 | 2.3863636363636327 | 2.1875000000000244 | 2.357954545454545 | 2.102272727272727 | 1.9034090909090808 | 1.931818181818182 | 1.9886363636363742 | 1.931818181818182 | 1.960227272727272 | 2.244318181818182 | 2.272727272727301 | 2.244318181818182 | 1.4488636363636358 | 1.5625 | 1.505681818181818 | 1.931818181818182 | 1.8181818181818181 | 1.931818181818182 | 1.477272727272727 | 2.0170454545454537 | 1.5625 | 1.53409090909091 | 1.3920454545454561 | 1.704545454545455 | 1.505681818181818 | 1.3920454545454561 | 1.363636363636363 | 1.3920454545454561 | 1.25 | 1.306818181818182 | 0.9375 | 1.477272727272727 | 1.2215909090909078 | 1.25 | 1.363636363636363 | 0.795454545454545 | 1.0227272727272718 | 1.0227272727272718 | 1.2215909090909078 | 0.9943181818181769 | 0.9659090909090972 | 0.9659090909090972 | 1.079545454545454 | 1.3352272727272718 |
| -35 | 3.977272727272727 | 3.465909090909091 | 4.431818181818239 | 2.840909090909091 | 2.272727272727301 | 2.4431818181818454 | 1.9886363636363742 | 3.0965909090909087 | 2.244318181818182 | 2.102272727272727 | 2.272727272727301 | 2.357954545454545 | 2.357954545454545 | 2.1590909090909087 | 1.9034090909090808 | 1.7329545454545459 | 2.244318181818182 | 2.357954545454545 | 1.8181818181818181 | 1.6761363636363742 | 2.0738636363636327 | 2.272727272727301 | 2.0170454545454537 | 1.6477272727272718 | 1.9034090909090808 | 1.3920454545454561 | 1.505681818181818 | 1.5625 | 1.84659090909091 | 1.505681818181818 | 1.53409090909091 | 1.25 | 1.1931818181818181 | 1.5909090909090808 | 1.2784090909090808 | 0.909090909090909 | 1.2784090909090808 | 0.9375 | 1.136363636363636 | 1.079545454545454 | 0.9943181818181769 | 1.0227272727272718 | 1.2784090909090808 | 0.852272727272727 | 1.1647727272727373 | 0.9375 | 0.9943181818181769 | 0.909090909090909 | 0.852272727272727 | 1.107954545454545 |
| -34 | 4.062499999999996 | 3.806818181818139 | 2.840909090909091 | 3.3238636363636327 | 2.897727272727301 | 2.8693181818181777 | 1.477272727272727 | 2.4715909090909087 | 2.8124999999999716 | 2.244318181818182 | 1.9034090909090808 | 1.9886363636363742 | 1.84659090909091 | 1.9886363636363742 | 2.215909090909091 | 2.244318181818182 | 2.0170454545454537 | 1.7329545454545459 | 1.960227272727272 | 1.875 | 1.8181818181818181 | 1.53409090909091 | 2.045454545454545 | 1.704545454545455 | 1.53409090909091 | 1.6477272727272718 | 1.363636363636363 | 1.3920454545454561 | 1.3920454545454561 | 1.363636363636363 | 1.53409090909091 | 1.5909090909090808 | 1.2784090909090808 | 1.1647727272727373 | 1.619318181818182 | 1.136363636363636 | 1.051136363636364 | 1.2784090909090808 | 0.9375 | 1.1647727272727373 | 1.051136363636364 | 1.1647727272727373 | 0.7670454545454652 | 0.738636363636363 | 1.1647727272727373 | 0.738636363636363 | 0.852272727272727 | 0.8238636363636421 | 1.25 | 0.8238636363636421 |
| -33 | 4.857954545454524 | 3.6363636363636327 | 3.210227272727301 | 2.6988636363636327 | 2.414772727272727 | 3.0113636363636327 | 1.9034090909090808 | 2.357954545454545 | 2.215909090909091 | 2.5 | 2.556818181818139 | 1.704545454545455 | 1.7613636363636358 | 1.960227272727272 | 1.84659090909091 | 2.1590909090909087 | 2.1875000000000244 | 1.9034090909090808 | 2.0738636363636327 | 1.875 | 1.7613636363636358 | 2.1590909090909087 | 1.6761363636363742 | 2.102272727272727 | 1.505681818181818 | 1.2784090909090808 | 1.8181818181818181 | 1.079545454545454 | 2.045454545454545 | 1.25 | 1.53409090909091 | 1.5625 | 1.136363636363636 | 1.107954545454545 | 1.3352272727272718 | 1.2215909090909078 | 1.1931818181818181 | 1.0227272727272718 | 1.1647727272727373 | 1.363636363636363 | 1.051136363636364 | 1.079545454545454 | 0.909090909090909 | 0.6534090909090972 | 1.25 | 0.6250000000000051 | 0.7670454545454652 | 0.9375 | 0.852272727272727 | 1.0227272727272718 |
| -32 | 4.630681818181816 | 4.346590909090906 | 5.028409090909091 | 3.551136363636364 | 3.522727272727301 | 2.8124999999999716 | 1.420454545454545 | 3.238636363636364 | 2.1590909090909087 | 2.6988636363636327 | 1.789772727272727 | 1.960227272727272 | 2.102272727272727 | 2.13068181818182 | 1.2215909090909078 | 1.9886363636363742 | 1.931818181818182 | 2.1875000000000244 | 2.3863636363636327 | 1.4488636363636358 | 1.960227272727272 | 2.0170454545454537 | 1.6761363636363742 | 1.3920454545454561 | 1.9886363636363742 | 1.931818181818182 | 1.5909090909090808 | 1.363636363636363 | 1.3920454545454561 | 1.1647727272727373 | 1.8181818181818181 | 1.25 | 1.5909090909090808 | 1.0227272727272718 | 1.25 | 1.1931818181818181 | 1.1931818181818181 | 1.1647727272727373 | 0.710227272727273 | 1.2784090909090808 | 0.852272727272727 | 1.0227272727272718 | 0.9943181818181769 | 0.5965909090909028 | 1.051136363636364 | 0.795454545454545 | 1.136363636363636 | 0.9659090909090972 | 0.8238636363636421 | 0.8238636363636421 |
| -31 | 5.511363636363691 | 4.005681818181817 | 2.8124999999999716 | 3.210227272727301 | 3.039772727272727 | 3.153409090909091 | 1.619318181818182 | 2.301136363636364 | 2.7272727272727506 | 2.4431818181818454 | 2.102272727272727 | 2.4431818181818454 | 1.363636363636363 | 2.4431818181818454 | 1.6477272727272718 | 1.931818181818182 | 2.4715909090909087 | 1.8181818181818181 | 1.9034090909090808 | 1.789772727272727 | 2.0738636363636327 | 1.420454545454545 | 1.704545454545455 | 1.6477272727272718 | 1.505681818181818 | 1.363636363636363 | 1.363636363636363 | 1.2784090909090808 | 1.505681818181818 | 1.6477272727272718 | 1.420454545454545 | 1.420454545454545 | 1.363636363636363 | 1.306818181818182 | 1.107954545454545 | 1.2784090909090808 | 1.079545454545454 | 0.9375 | 0.710227272727273 | 0.8806818181818241 | 0.9943181818181769 | 0.7670454545454652 | 0.7670454545454652 | 0.6534090909090972 | 0.795454545454545 | 0.5681818181818231 | 1.2784090909090808 | 0.9375 | 0.795454545454545 | 1.0227272727272718 |
| -30 | 5.795454545454545 | 4.687499999999995 | 3.1818181818181777 | 4.1761363636363615 | 3.38068181818182 | 2.8693181818181777 | 1.4488636363636358 | 2.3863636363636327 | 2.13068181818182 | 2.6420454545454537 | 2.3863636363636327 | 2.244318181818182 | 1.7329545454545459 | 2.244318181818182 | 1.84659090909091 | 1.7613636363636358 | 1.8181818181818181 | 1.7329545454545459 | 2.0170454545454537 | 1.9034090909090808 | 2.045454545454545 | 2.102272727272727 | 1.704545454545455 | 1.7329545454545459 | 1.1931818181818181 | 1.704545454545455 | 1.477272727272727 | 1.4488636363636358 | 1.25 | 1.5909090909090808 | 1.505681818181818 | 1.6477272727272718 | 1.3352272727272718 | 1.107954545454545 | 1.136363636363636 | 0.9375 | 1.051136363636364 | 0.9943181818181769 | 0.9659090909090972 | 0.9943181818181769 | 0.738636363636363 | 1.136363636363636 | 0.8238636363636421 | 0.681818181818182 | 0.8238636363636421 | 0.8238636363636421 | 1.0227272727272718 | 0.4829545454545481 | 0.5965909090909028 | 0.852272727272727 |
| -29 | 5.4261363636363615 | 4.403409090909151 | 5.142045454545435 | 4.1761363636363615 | 2.926136363636364 | 3.6363636363636327 | 1.3920454545454561 | 3.551136363636364 | 2.7840909090909105 | 2.357954545454545 | 2.8693181818181777 | 2.5 | 1.8181818181818181 | 2.1590909090909087 | 1.420454545454545 | 1.789772727272727 | 1.875 | 2.3295454545454537 | 1.960227272727272 | 2.244318181818182 | 1.619318181818182 | 1.8181818181818181 | 1.7329545454545459 | 1.3920454545454561 | 1.6477272727272718 | 1.5625 | 1.6477272727272718 | 1.3352272727272718 | 1.420454545454545 | 1.704545454545455 | 1.3352272727272718 | 1.25 | 1.704545454545455 | 1.53409090909091 | 1.2784090909090808 | 1.1931818181818181 | 1.079545454545454 | 1.1931818181818181 | 0.8806818181818241 | 0.8238636363636421 | 0.852272727272727 | 0.852272727272727 | 0.909090909090909 | 0.681818181818182 | 1.0227272727272718 | 0.6534090909090972 | 0.8238636363636421 | 0.5965909090909028 | 0.5965909090909028 | 0.8238636363636421 |
| -28 | 6.022727272727264 | 4.744318181818183 | 3.295454545454545 | 3.3238636363636327 | 4.062499999999996 | 3.522727272727301 | 1.477272727272727 | 2.528409090909091 | 2.7840909090909105 | 2.244318181818182 | 2.585227272727301 | 2.357954545454545 | 2.102272727272727 | 2.3863636363636327 | 1.3352272727272718 | 2.13068181818182 | 2.102272727272727 | 2.1590909090909087 | 2.3863636363636327 | 1.5625 | 2.0738636363636327 | 2.0170454545454537 | 2.045454545454545 | 1.25 | 1.25 | 1.5625 | 1.931818181818182 | 0.9375 | 1.4488636363636358 | 1.3352272727272718 | 1.2215909090909078 | 1.1647727272727373 | 1.1931818181818181 | 1.25 | 0.738636363636363 | 0.9943181818181769 | 0.852272727272727 | 0.909090909090909 | 0.6534090909090972 | 0.7670454545454652 | 1.1931818181818181 | 0.909090909090909 | 0.5965909090909028 | 0.8238636363636421 | 0.681818181818182 | 0.710227272727273 | 0.9659090909090972 | 0.5965909090909028 | 0.795454545454545 | 0.681818181818182 |
| -27 | 6.022727272727264 | 4.545454545454545 | 3.5795454545454537 | 4.573863636363691 | 3.7215909090909105 | 3.267045454545454 | 1.9034090909090808 | 2.6420454545454537 | 2.6988636363636327 | 2.556818181818139 | 3.465909090909091 | 2.3863636363636327 | 2.244318181818182 | 2.6988636363636327 | 1.4488636363636358 | 1.7613636363636358 | 2.1875000000000244 | 1.931818181818182 | 1.789772727272727 | 1.8181818181818181 | 1.505681818181818 | 1.7329545454545459 | 1.5625 | 1.306818181818182 | 1.477272727272727 | 1.619318181818182 | 1.6761363636363742 | 1.2215909090909078 | 1.5625 | 1.363636363636363 | 1.477272727272727 | 1.619318181818182 | 1.5625 | 0.909090909090909 | 0.8806818181818241 | 0.795454545454545 | 0.7670454545454652 | 0.6534090909090972 | 0.5965909090909028 | 0.710227272727273 | 0.6250000000000051 | 0.710227272727273 | 0.539772727272727 | 0.852272727272727 | 0.7670454545454652 | 0.5681818181818231 | 0.8238636363636421 | 0.539772727272727 | 0.8238636363636421 | 0.8806818181818241 |
| -26 | 6.164772727272727 | 5.1136363636363615 | 5.568181818181755 | 4.857954545454524 | 3.6931818181818454 | 3.3522727272727177 | 1.8181818181818181 | 3.75 | 2.528409090909091 | 2.4715909090909087 | 3.977272727272727 | 2.4715909090909087 | 2.244318181818182 | 2.3295454545454537 | 1.53409090909091 | 1.5625 | 1.7613636363636358 | 1.8181818181818181 | 1.931818181818182 | 1.7329545454545459 | 1.789772727272727 | 1.9886363636363742 | 1.6761363636363742 | 1.363636363636363 | 1.505681818181818 | 1.931818181818182 | 1.3352272727272718 | 1.0227272727272718 | 1.53409090909091 | 1.420454545454545 | 1.3920454545454561 | 1.3352272727272718 | 1.107954545454545 | 1.2215909090909078 | 0.795454545454545 | 0.9659090909090972 | 1.0227272727272718 | 0.42613636363636404 | 0.738636363636363 | 0.710227272727273 | 0.681818181818182 | 1.0227272727272718 | 0.9659090909090972 | 0.681818181818182 | 0.710227272727273 | 0.6534090909090972 | 0.6534090909090972 | 0.795454545454545 | 0.6250000000000051 | 0.8238636363636421 |
| -25 | 5.312499999999996 | 4.772727272727272 | 3.6363636363636327 | 5.4261363636363615 | 3.835227272727275 | 3.522727272727301 | 2.670454545454545 | 2.414772727272727 | 2.840909090909091 | 2.670454545454545 | 3.0965909090909087 | 2.045454545454545 | 2.3295454545454537 | 3.210227272727301 | 1.505681818181818 | 1.6761363636363742 | 1.6761363636363742 | 1.84659090909091 | 2.13068181818182 | 1.9886363636363742 | 1.477272727272727 | 1.477272727272727 | 1.5909090909090808 | 1.5625 | 1.25 | 1.6761363636363742 | 1.477272727272727 | 1.1931818181818181 | 1.477272727272727 | 1.3352272727272718 | 1.136363636363636 | 1.1931818181818181 | 1.2215909090909078 | 0.710227272727273 | 0.9375 | 0.8238636363636421 | 1.0227272727272718 | 0.738636363636363 | 0.6250000000000051 | 0.6534090909090972 | 0.795454545454545 | 0.6534090909090972 | 0.6534090909090972 | 0.4829545454545481 | 0.454545454545454 | 0.6250000000000051 | 0.42613636363636404 | 0.909090909090909 | 0.909090909090909 | 1.0227272727272718 |
| -24 | 6.079545454545454 | 4.914772727272728 | 3.3522727272727177 | 5.62499999999996 | 4.289772727272752 | 3.465909090909091 | 4.090909090909091 | 2.8124999999999716 | 2.9545454545454537 | 2.301136363636364 | 4.0340909090909065 | 1.84659090909091 | 2.5 | 2.897727272727301 | 1.875 | 1.4488636363636358 | 1.789772727272727 | 1.931818181818182 | 2.272727272727301 | 1.789772727272727 | 1.5909090909090808 | 1.875 | 1.619318181818182 | 1.051136363636364 | 1.704545454545455 | 2.0170454545454537 | 1.1931818181818181 | 1.1931818181818181 | 0.852272727272727 | 1.3352272727272718 | 1.107954545454545 | 0.738636363636363 | 1.1647727272727373 | 0.9943181818181769 | 0.909090909090909 | 0.8806818181818241 | 1.051136363636364 | 0.710227272727273 | 0.511363636363636 | 0.6534090909090972 | 0.738636363636363 | 0.681818181818182 | 0.5681818181818231 | 0.5681818181818231 | 0.738636363636363 | 0.6250000000000051 | 0.5965909090909028 | 0.511363636363636 | 0.454545454545454 | 0.795454545454545 |
| -23 | 6.676136363636361 | 4.772727272727272 | 5.227272727272728 | 6.363636363636361 | 5.312499999999996 | 2.982954545454545 | 4.090909090909091 | 2.982954545454545 | 3.6931818181818454 | 2.0170454545454537 | 3.3238636363636327 | 2.4715909090909087 | 2.6988636363636327 | 3.3522727272727177 | 2.414772727272727 | 1.6477272727272718 | 1.704545454545455 | 1.9886363636363742 | 1.9886363636363742 | 1.6761363636363742 | 1.619318181818182 | 1.477272727272727 | 1.5909090909090808 | 1.079545454545454 | 1.306818181818182 | 1.363636363636363 | 1.420454545454545 | 1.505681818181818 | 0.9943181818181769 | 1.363636363636363 | 0.8806818181818241 | 1.107954545454545 | 0.9943181818181769 | 0.9943181818181769 | 0.454545454545454 | 0.5965909090909028 | 0.795454545454545 | 0.6250000000000051 | 0.6534090909090972 | 0.28409090909091206 | 0.7670454545454652 | 0.7670454545454652 | 0.5965909090909028 | 0.681818181818182 | 0.5681818181818231 | 0.539772727272727 | 0.4829545454545481 | 0.539772727272727 | 0.511363636363636 | 0.539772727272727 |
| -22 | 8.636363636363635 | 5.1136363636363615 | 3.38068181818182 | 5.4261363636363615 | 4.346590909090906 | 3.039772727272727 | 5.681818181818183 | 1.9034090909090808 | 2.982954545454545 | 2.272727272727301 | 2.7840909090909105 | 2.244318181818182 | 2.7840909090909105 | 2.75568181818182 | 2.4431818181818454 | 1.8181818181818181 | 1.25 | 1.53409090909091 | 1.84659090909091 | 1.3920454545454561 | 1.5625 | 1.306818181818182 | 1.0227272727272718 | 1.136363636363636 | 1.0227272727272718 | 1.2215909090909078 | 1.0227272727272718 | 1.5909090909090808 | 0.909090909090909 | 1.1931818181818181 | 0.9943181818181769 | 1.1931818181818181 | 1.079545454545454 | 1.0227272727272718 | 0.795454545454545 | 0.8806818181818241 | 0.795454545454545 | 0.6250000000000051 | 0.6534090909090972 | 0.681818181818182 | 0.5965909090909028 | 0.5681818181818231 | 0.454545454545454 | 0.8806818181818241 | 0.539772727272727 | 0.795454545454545 | 0.511363636363636 | 0.5681818181818231 | 0.852272727272727 | 0.5681818181818231 |
| -21 | 7.556818181818183 | 4.772727272727272 | 4.630681818181816 | 6.25 | 3.8920454545454164 | 2.670454545454545 | 5.397727272727264 | 1.6761363636363742 | 3.0113636363636327 | 2.215909090909091 | 2.13068181818182 | 2.8693181818181777 | 3.4943181818181777 | 2.6420454545454537 | 2.840909090909091 | 2.102272727272727 | 1.5625 | 1.53409090909091 | 1.5909090909090808 | 0.8238636363636421 | 1.051136363636364 | 1.363636363636363 | 1.079545454545454 | 0.9375 | 1.107954545454545 | 1.3920454545454561 | 1.306818181818182 | 1.9034090909090808 | 0.6534090909090972 | 0.9375 | 1.079545454545454 | 0.6534090909090972 | 1.306818181818182 | 1.079545454545454 | 0.9375 | 0.9375 | 0.681818181818182 | 0.5681818181818231 | 0.6534090909090972 | 0.3693181818181861 | 0.7670454545454652 | 0.795454545454545 | 0.6250000000000051 | 1.107954545454545 | 0.6534090909090972 | 0.9659090909090972 | 0.511363636363636 | 0.738636363636363 | 0.681818181818182 | 0.6534090909090972 |
| -20 | 7.130681818181817 | 4.460227272727272 | 8.323863636363637 | 3.75 | 3.0113636363636327 | 2.7272727272727506 | 5.539772727272752 | 1.9034090909090808 | 3.153409090909091 | 2.4431818181818454 | 1.84659090909091 | 3.465909090909091 | 2.7272727272727506 | 2.301136363636364 | 3.75 | 2.244318181818182 | 1.6477272727272718 | 1.53409090909091 | 1.2215909090909078 | 1.2784090909090808 | 0.9659090909090972 | 0.9659090909090972 | 1.306818181818182 | 1.079545454545454 | 1.3920454545454561 | 0.511363636363636 | 1.3920454545454561 | 2.13068181818182 | 0.9375 | 0.9943181818181769 | 1.079545454545454 | 1.0227272727272718 | 1.0227272727272718 | 0.8238636363636421 | 1.079545454545454 | 0.9375 | 0.6250000000000051 | 0.9375 | 0.909090909090909 | 0.7670454545454652 | 0.681818181818182 | 0.6534090909090972 | 1.136363636363636 | 1.3920454545454561 | 0.8238636363636421 | 1.136363636363636 | 0.710227272727273 | 0.710227272727273 | 0.852272727272727 | 0.198863636363636 |
| -19 | 5.170454545454545 | 4.232954545454545 | 5.028409090909091 | 2.4715909090909087 | 2.357954545454545 | 2.585227272727301 | 3.835227272727275 | 1.5909090909090808 | 1.9886363636363742 | 3.7215909090909105 | 1.704545454545455 | 3.551136363636364 | 3.4374999999999987 | 0.8238636363636421 | 3.75 | 2.6420454545454537 | 1.6477272727272718 | 1.7613636363636358 | 1.4488636363636358 | 1.3920454545454561 | 1.306818181818182 | 1.136363636363636 | 1.0227272727272718 | 1.0227272727272718 | 1.306818181818182 | 1.0227272727272718 | 1.789772727272727 | 2.272727272727301 | 1.4488636363636358 | 1.53409090909091 | 1.107954545454545 | 1.25 | 1.136363636363636 | 1.3920454545454561 | 1.2784090909090808 | 1.136363636363636 | 0.5965909090909028 | 0.852272727272727 | 1.136363636363636 | 0.8806818181818241 | 0.9943181818181769 | 0.795454545454545 | 0.9375 | 1.25 | 0.5681818181818231 | 1.25 | 0.6534090909090972 | 0.8238636363636421 | 0.8238636363636421 | 0.511363636363636 |
| -18 | 2.6988636363636327 | 4.431818181818239 | 4.403409090909151 | 2.301136363636364 | 1.5625 | 2.897727272727301 | 1.931818181818182 | 1.53409090909091 | 1.960227272727272 | 2.897727272727301 | 1.619318181818182 | 2.897727272727301 | 2.556818181818139 | 1.306818181818182 | 3.7215909090909105 | 2.840909090909091 | 2.1590909090909087 | 2.8124999999999716 | 1.7329545454545459 | 1.5625 | 1.2784090909090808 | 1.051136363636364 | 1.619318181818182 | 1.84659090909091 | 1.8181818181818181 | 1.107954545454545 | 1.789772727272727 | 2.0170454545454537 | 1.1931818181818181 | 1.53409090909091 | 1.477272727272727 | 1.9034090909090808 | 1.1931818181818181 | 1.1647727272727373 | 1.84659090909091 | 1.079545454545454 | 0.710227272727273 | 0.738636363636363 | 1.3352272727272718 | 1.107954545454545 | 0.738636363636363 | 1.306818181818182 | 0.909090909090909 | 1.3920454545454561 | 1.136363636363636 | 0.8806818181818241 | 0.909090909090909 | 1.25 | 0.9375 | 0.454545454545454 |
| -17 | 2.045454545454545 | 3.6647727272727506 | 5.880681818181817 | 2.13068181818182 | 1.25 | 2.982954545454545 | 1.505681818181818 | 3.77840909090909 | 1.3920454545454561 | 2.670454545454545 | 1.619318181818182 | 2.556818181818139 | 1.8181818181818181 | 1.1647727272727373 | 2.102272727272727 | 2.556818181818139 | 2.670454545454545 | 2.9545454545454537 | 1.7329545454545459 | 1.960227272727272 | 2.0170454545454537 | 1.5909090909090808 | 1.53409090909091 | 2.1590909090909087 | 1.7613636363636358 | 1.420454545454545 | 1.619318181818182 | 1.619318181818182 | 1.960227272727272 | 1.5909090909090808 | 1.789772727272727 | 2.13068181818182 | 1.5909090909090808 | 1.5909090909090808 | 1.420454545454545 | 0.9943181818181769 | 0.738636363636363 | 1.107954545454545 | 1.363636363636363 | 1.363636363636363 | 0.909090909090909 | 1.25 | 1.2784090909090808 | 0.909090909090909 | 1.2784090909090808 | 1.2215909090909078 | 0.9375 | 1.1931818181818181 | 0.852272727272727 | 0.795454545454545 |B Phaeodactylum tricornutum (P diatom)
### Chart
| Category | AAAAU | UUAAA | UAAAU | AAAUU | UUUAA | AUUUU | UAAAA | AAAUA | AAUUU | UUUUA | UAUUU | AUAAA | UAAUU | AUUUA | UUAUU | AAUUA | UUUUU | AUUAA | AAUAA | UUAAU | UUUAU | AAAAA | AAUAU | AUAUU | AUUAU | UAUUA | AUAAU | UAAUA | AUUCA | UUAUA | UAUAA | CUUUU | UCAAA | UUCAA | AUAUA | UUUCA | UUUUC | UAUAU | CAAAA | UUCAU | UUCUU | AAAUC | AAUUC | UUUCU | AUUUC | ACUUU | UUACU | UCAUU | UUUUG | AUCUU |
|---|---|---|---|---|---|---|---|---|---|---|---|---|---|---|---|---|---|---|---|---|---|---|---|---|---|---|---|---|---|---|---|---|---|---|---|---|---|---|---|---|---|---|---|---|---|---|---|---|---|---|
| -20 | 1.465738365701722 | 1.4290949065591676 | 1.355807988274093 | 1.282521069989007 | 1.9054598754122392 | 1.612312202271894 | 1.9421033345547902 | 1.0260168559912133 | 1.465738365701722 | 1.465738365701722 | 2.235251007695127 | 1.2092341517039198 | 1.539025283986808 | 1.648955661414438 | 1.3191645291315501 | 0.8427995602784992 | 0.8427995602784992 | 1.06266031513375 | 1.2458776108464638 | 0.9893733968486589 | 0.8427995602784992 | 0.6229388054232341 | 1.1725906925613778 | 0.8794430194210341 | 0.769512641993404 | 0.8061561011359418 | 0.8794430194210341 | 0.696225723708318 | 0.4763649688530641 | 0.8427995602784992 | 0.5496518871381512 | 0.513008427995603 | 0.36643459142543205 | 0.32979113228288803 | 0.4763649688530641 | 0.32979113228288803 | 0.586295346280689 | 0.32979113228288803 | 0.36643459142543205 | 0.21986075485525802 | 0.403078050567974 | 0.32979113228288803 | 0.21986075485525802 | 0.18321729571271705 | 0.513008427995603 | 0.586295346280689 | 0.6595822645657761 | 0.32979113228288803 | 0.43972150971051704 | 0.43972150971051704 |
| -19 | 1.465738365701722 | 2.125320630267501 | 1.0260168559912133 | 1.612312202271894 | 2.015390252839868 | 2.4184683034078156 | 1.6855991205569811 | 1.1725906925613778 | 1.648955661414438 | 1.4290949065591676 | 1.758886038842067 | 1.7222425796995322 | 1.648955661414438 | 1.1725906925613778 | 0.9527299377061241 | 0.9527299377061241 | 1.0993037742762921 | 1.06266031513375 | 0.732869182850861 | 1.0993037742762921 | 1.0993037742762921 | 1.4290949065591676 | 0.9893733968486589 | 0.9160864785635812 | 0.696225723708318 | 0.8794430194210341 | 0.6229388054232341 | 0.513008427995603 | 0.4763649688530641 | 0.6229388054232341 | 0.732869182850861 | 0.586295346280689 | 0.43972150971051704 | 0.5496518871381512 | 0.403078050567974 | 0.769512641993404 | 0.43972150971051704 | 0.732869182850861 | 0.43972150971051704 | 0.10993037742763002 | 0.586295346280689 | 0.2565042139978051 | 0.4763649688530641 | 0.4763649688530641 | 0.43972150971051704 | 0.513008427995603 | 0.403078050567974 | 0.43972150971051704 | 0.10993037742763002 | 0.36643459142543205 |
| -18 | 2.3085379259802132 | 2.16196408941004 | 2.015390252839868 | 1.5023818248442733 | 1.7222425796995322 | 2.015390252839868 | 1.7222425796995322 | 1.0260168559912133 | 1.758886038842067 | 1.7955294979845946 | 1.1359472334188352 | 0.9893733968486589 | 1.1725906925613778 | 1.2092341517039198 | 1.2458776108464638 | 1.06266031513375 | 1.3191645291315501 | 1.06266031513375 | 0.6595822645657761 | 1.06266031513375 | 1.0260168559912133 | 0.732869182850861 | 0.8061561011359418 | 1.1725906925613778 | 0.769512641993404 | 0.9527299377061241 | 0.6595822645657761 | 0.732869182850861 | 0.513008427995603 | 0.586295346280689 | 0.5496518871381512 | 0.43972150971051704 | 0.586295346280689 | 0.6595822645657761 | 0.513008427995603 | 0.586295346280689 | 0.8061561011359418 | 0.513008427995603 | 0.403078050567974 | 0.732869182850861 | 0.403078050567974 | 0.21986075485525802 | 0.586295346280689 | 0.29314767314034607 | 0.2565042139978051 | 0.513008427995603 | 0.5496518871381512 | 0.36643459142543205 | 0.43972150971051704 | 0.6595822645657761 |
| -17 | 1.282521069989007 | 1.978746793697325 | 1.7222425796995322 | 1.8688164162696959 | 1.832172957127153 | 1.392451447416636 | 1.4290949065591676 | 1.7222425796995322 | 1.392451447416636 | 1.758886038842067 | 1.392451447416636 | 0.9160864785635812 | 1.1359472334188352 | 1.1725906925613778 | 1.392451447416636 | 1.0993037742762921 | 1.392451447416636 | 1.465738365701722 | 0.8427995602784992 | 0.9893733968486589 | 1.392451447416636 | 0.9893733968486589 | 0.769512641993404 | 0.9893733968486589 | 0.6595822645657761 | 1.06266031513375 | 0.586295346280689 | 0.696225723708318 | 0.8061561011359418 | 0.696225723708318 | 0.769512641993404 | 0.732869182850861 | 0.696225723708318 | 0.696225723708318 | 0.586295346280689 | 0.586295346280689 | 0.732869182850861 | 0.513008427995603 | 0.5496518871381512 | 0.4763649688530641 | 0.43972150971051704 | 0.8061561011359418 | 0.2565042139978051 | 0.696225723708318 | 0.4763649688530641 | 0.403078050567974 | 0.32979113228288803 | 0.43972150971051704 | 0.513008427995603 | 0.32979113228288803 |
| -16 | 1.7955294979845946 | 2.491755221692928 | 1.355807988274093 | 1.539025283986808 | 2.0520337119824212 | 1.6855991205569811 | 1.612312202271894 | 1.355807988274093 | 1.539025283986808 | 1.648955661414438 | 1.2092341517039198 | 1.2092341517039198 | 0.9527299377061241 | 1.3191645291315501 | 0.9893733968486589 | 1.355807988274093 | 1.6855991205569811 | 1.355807988274093 | 1.2458776108464638 | 0.9160864785635812 | 0.732869182850861 | 1.539025283986808 | 1.06266031513375 | 0.769512641993404 | 0.8061561011359418 | 0.8427995602784992 | 0.732869182850861 | 0.696225723708318 | 0.43972150971051704 | 0.732869182850861 | 0.586295346280689 | 0.769512641993404 | 0.8061561011359418 | 0.8794430194210341 | 0.586295346280689 | 0.6229388054232341 | 0.6595822645657761 | 0.513008427995603 | 0.5496518871381512 | 0.6595822645657761 | 0.43972150971051704 | 0.14657383657017303 | 0.43972150971051704 | 0.6229388054232341 | 0.14657383657017303 | 0.403078050567974 | 0.403078050567974 | 0.4763649688530641 | 0.5496518871381512 | 0.36643459142543205 |
| -15 | 1.539025283986808 | 2.2718944668376704 | 1.9421033345547902 | 1.392451447416636 | 1.648955661414438 | 1.2092341517039198 | 1.6855991205569811 | 1.539025283986808 | 1.612312202271894 | 1.648955661414438 | 0.9527299377061241 | 1.2458776108464638 | 0.8061561011359418 | 1.355807988274093 | 0.8794430194210341 | 1.06266031513375 | 0.9527299377061241 | 1.392451447416636 | 1.4290949065591676 | 0.9893733968486589 | 1.0993037742762921 | 1.282521069989007 | 0.6229388054232341 | 1.0993037742762921 | 0.6595822645657761 | 0.696225723708318 | 0.32979113228288803 | 0.696225723708318 | 0.6229388054232341 | 0.6595822645657761 | 0.696225723708318 | 0.586295346280689 | 0.8061561011359418 | 0.696225723708318 | 0.513008427995603 | 0.6229388054232341 | 0.586295346280689 | 0.403078050567974 | 0.36643459142543205 | 0.5496518871381512 | 0.36643459142543205 | 0.4763649688530641 | 0.2565042139978051 | 0.43972150971051704 | 0.403078050567974 | 0.36643459142543205 | 0.36643459142543205 | 0.29314767314034607 | 0.36643459142543205 | 0.36643459142543205 |
| -14 | 2.015390252839868 | 1.832172957127153 | 2.088677171124955 | 1.7222425796995322 | 2.2718944668376704 | 1.2458776108464638 | 1.7955294979845946 | 1.758886038842067 | 0.8427995602784992 | 1.4290949065591676 | 1.0993037742762921 | 1.9054598754122392 | 1.06266031513375 | 1.2092341517039198 | 1.1725906925613778 | 1.0993037742762921 | 0.8061561011359418 | 1.282521069989007 | 0.9527299377061241 | 1.2458776108464638 | 0.9893733968486589 | 0.9160864785635812 | 0.9527299377061241 | 0.586295346280689 | 0.5496518871381512 | 0.696225723708318 | 0.513008427995603 | 0.513008427995603 | 0.403078050567974 | 0.403078050567974 | 0.586295346280689 | 0.5496518871381512 | 0.5496518871381512 | 0.586295346280689 | 0.32979113228288803 | 0.513008427995603 | 0.21986075485525802 | 0.403078050567974 | 0.43972150971051704 | 0.5496518871381512 | 0.43972150971051704 | 0.5496518871381512 | 0.36643459142543205 | 0.6229388054232341 | 0.5496518871381512 | 0.4763649688530641 | 0.32979113228288803 | 0.5496518871381512 | 0.5496518871381512 | 0.29314767314034607 |
| -13 | 2.198607548552611 | 1.832172957127153 | 2.16196408941004 | 1.758886038842067 | 1.612312202271894 | 0.8794430194210341 | 1.5023818248442733 | 2.235251007695127 | 1.2092341517039198 | 1.0993037742762921 | 0.8794430194210341 | 1.2092341517039198 | 1.2092341517039198 | 1.06266031513375 | 1.0993037742762921 | 1.465738365701722 | 0.9893733968486589 | 1.1725906925613778 | 1.282521069989007 | 1.3191645291315501 | 1.0993037742762921 | 0.9893733968486589 | 1.1725906925613778 | 0.8061561011359418 | 0.513008427995603 | 0.696225723708318 | 0.586295346280689 | 0.586295346280689 | 0.732869182850861 | 0.4763649688530641 | 0.43972150971051704 | 0.403078050567974 | 0.5496518871381512 | 0.32979113228288803 | 0.43972150971051704 | 0.36643459142543205 | 0.513008427995603 | 0.18321729571271705 | 0.5496518871381512 | 0.696225723708318 | 0.5496518871381512 | 0.5496518871381512 | 0.5496518871381512 | 0.36643459142543205 | 0.21986075485525802 | 0.18321729571271705 | 0.29314767314034607 | 0.43972150971051704 | 0.43972150971051704 | 0.21986075485525802 |
| -12 | 1.832172957127153 | 1.1359472334188352 | 1.648955661414438 | 1.978746793697325 | 1.06266031513375 | 1.1359472334188352 | 1.1359472334188352 | 1.9054598754122392 | 1.1359472334188352 | 1.1725906925613778 | 1.1725906925613778 | 0.9160864785635812 | 1.355807988274093 | 0.9893733968486589 | 1.1725906925613778 | 1.539025283986808 | 0.732869182850861 | 1.1359472334188352 | 1.758886038842067 | 1.282521069989007 | 1.0993037742762921 | 0.696225723708318 | 1.2092341517039198 | 0.9160864785635812 | 0.8427995602784992 | 0.32979113228288803 | 0.586295346280689 | 0.6229388054232341 | 0.6229388054232341 | 0.5496518871381512 | 0.36643459142543205 | 0.403078050567974 | 0.403078050567974 | 0.43972150971051704 | 0.513008427995603 | 0.21986075485525802 | 0.2565042139978051 | 0.5496518871381512 | 0.403078050567974 | 0.513008427995603 | 0.43972150971051704 | 0.6229388054232341 | 0.6229388054232341 | 0.586295346280689 | 0.32979113228288803 | 0.29314767314034607 | 0.513008427995603 | 0.36643459142543205 | 0.2565042139978051 | 0.43972150971051704 |
| -11 | 1.2458776108464638 | 1.282521069989007 | 1.0260168559912133 | 1.5756687431293428 | 0.8794430194210341 | 1.4290949065591676 | 1.355807988274093 | 1.4290949065591676 | 1.9054598754122392 | 0.9527299377061241 | 1.392451447416636 | 1.3191645291315501 | 1.1725906925613778 | 1.0260168559912133 | 1.282521069989007 | 1.2092341517039198 | 1.392451447416636 | 0.9527299377061241 | 1.465738365701722 | 1.0260168559912133 | 1.0260168559912133 | 1.1359472334188352 | 0.9893733968486589 | 1.1725906925613778 | 1.1359472334188352 | 0.732869182850861 | 1.0993037742762921 | 0.586295346280689 | 0.513008427995603 | 0.513008427995603 | 0.586295346280689 | 0.732869182850861 | 0.4763649688530641 | 0.43972150971051704 | 0.696225723708318 | 0.32979113228288803 | 0.36643459142543205 | 0.5496518871381512 | 0.6229388054232341 | 0.29314767314034607 | 0.43972150971051704 | 0.6595822645657761 | 0.6229388054232341 | 0.14657383657017303 | 0.403078050567974 | 0.403078050567974 | 0.43972150971051704 | 0.29314767314034607 | 0.43972150971051704 | 0.29314767314034607 |
| -10 | 1.5756687431293428 | 0.9160864785635812 | 1.355807988274093 | 1.282521069989007 | 0.769512641993404 | 1.5023818248442733 | 1.2092341517039198 | 0.9893733968486589 | 1.465738365701722 | 1.1725906925613778 | 1.5023818248442733 | 1.2092341517039198 | 1.1725906925613778 | 1.2092341517039198 | 1.2092341517039198 | 1.1359472334188352 | 1.0260168559912133 | 0.8061561011359418 | 1.2092341517039198 | 1.0993037742762921 | 1.06266031513375 | 0.696225723708318 | 0.9160864785635812 | 1.355807988274093 | 0.8794430194210341 | 0.732869182850861 | 1.0993037742762921 | 0.696225723708318 | 0.513008427995603 | 0.43972150971051704 | 0.513008427995603 | 0.403078050567974 | 0.403078050567974 | 0.403078050567974 | 0.513008427995603 | 0.43972150971051704 | 0.4763649688530641 | 0.6595822645657761 | 0.586295346280689 | 0.403078050567974 | 0.36643459142543205 | 0.6229388054232341 | 0.5496518871381512 | 0.43972150971051704 | 0.6595822645657761 | 0.43972150971051704 | 0.32979113228288803 | 0.43972150971051704 | 0.43972150971051704 | 0.403078050567974 |
| -9 | 1.6855991205569811 | 0.732869182850861 | 1.392451447416636 | 1.3191645291315501 | 0.8794430194210341 | 1.612312202271894 | 0.8427995602784992 | 1.282521069989007 | 1.06266031513375 | 1.3191645291315501 | 1.7222425796995322 | 0.9160864785635812 | 1.539025283986808 | 0.9893733968486589 | 1.392451447416636 | 1.1359472334188352 | 1.612312202271894 | 0.8427995602784992 | 0.513008427995603 | 0.8427995602784992 | 1.1359472334188352 | 0.769512641993404 | 1.0260168559912133 | 0.9527299377061241 | 0.9160864785635812 | 0.5496518871381512 | 1.0993037742762921 | 0.586295346280689 | 0.9527299377061241 | 0.5496518871381512 | 0.36643459142543205 | 0.513008427995603 | 0.36643459142543205 | 0.403078050567974 | 0.8061561011359418 | 0.732869182850861 | 0.403078050567974 | 0.513008427995603 | 0.513008427995603 | 0.513008427995603 | 0.696225723708318 | 0.32979113228288803 | 0.36643459142543205 | 0.32979113228288803 | 0.696225723708318 | 0.43972150971051704 | 0.403078050567974 | 0.4763649688530641 | 0.36643459142543205 | 0.6595822645657761 |C Tetrahymena thermophila (Ciliate)
Signal frequency %
### Chart
| Category | UGUAA | UUGUA | GUAAU | UAAUA | UUUGU | AAUAA | UUAAU | UAAUU | UUUAA | GUUGU | UUUUA | AAUUU | GUAAA | AUGUA | UAACA | UAAUC | UCAAU | AUUUU | AAUAU | UAAAA | GUAAC | AAUCA | AAAAU | AUGAA | AAUAC | AACAA | CUUGU | UUUUG | AUCAA | UAAAU | UUGUU | AAUGA | GUGUA | UGAAA | CAAUA | AUUGU | UGAAU | GUUUU | AAUCU | AUUAA | AUAAA | UGUAU | UUUCA | ACAAC | CUGUA | AAAUC | UAUUA | GAUGU | AUUUA | AAAUU |
|---|---|---|---|---|---|---|---|---|---|---|---|---|---|---|---|---|---|---|---|---|---|---|---|---|---|---|---|---|---|---|---|---|---|---|---|---|---|---|---|---|---|---|---|---|---|---|---|---|---|---|
| -35 | 0.12771392081737104 | 0.12771392081737104 | 0.12771392081737104 | 0.0 | 0.383141762452107 | 0.5108556832694832 | 0.25542784163473803 | 0.12771392081737104 | 0.12771392081737104 | 0.12771392081737104 | 0.5108556832694832 | 0.25542784163473803 | 0.383141762452107 | 0.383141762452107 | 0.12771392081737104 | 0.12771392081737104 | 0.25542784163473803 | 0.383141762452107 | 0.12771392081737104 | 0.383141762452107 | 0.12771392081737104 | 0.12771392081737104 | 0.25542784163473803 | 0.0 | 0.12771392081737104 | 0.12771392081737104 | 0.12771392081737104 | 0.383141762452107 | 0.383141762452107 | 0.0 | 0.0 | 0.0 | 0.25542784163473803 | 0.383141762452107 | 0.0 | 0.12771392081737104 | 0.25542784163473803 | 0.25542784163473803 | 0.0 | 0.0 | 0.12771392081737104 | 0.12771392081737104 | 0.12771392081737104 | 0.12771392081737104 | 0.12771392081737104 | 0.383141762452107 | 0.0 | 0.383141762452107 | 0.25542784163473803 | 0.25542784163473803 |
| -34 | 0.25542784163473803 | 0.25542784163473803 | 0.12771392081737104 | 0.25542784163473803 | 1.1494252873563218 | 0.0 | 0.0 | 0.25542784163473803 | 0.5108556832694832 | 0.5108556832694832 | 0.0 | 0.5108556832694832 | 0.0 | 0.25542784163473803 | 0.0 | 0.12771392081737104 | 0.25542784163473803 | 0.25542784163473803 | 0.0 | 0.383141762452107 | 0.0 | 0.0 | 0.7662835249042149 | 0.12771392081737104 | 0.0 | 0.25542784163473803 | 0.12771392081737104 | 0.5108556832694832 | 0.12771392081737104 | 0.0 | 0.12771392081737104 | 0.0 | 0.25542784163473803 | 0.25542784163473803 | 0.383141762452107 | 0.12771392081737104 | 0.12771392081737104 | 0.5108556832694832 | 0.0 | 0.0 | 0.12771392081737104 | 0.25542784163473803 | 0.25542784163473803 | 0.383141762452107 | 0.0 | 0.383141762452107 | 0.383141762452107 | 0.383141762452107 | 0.12771392081737104 | 0.25542784163473803 |
| -33 | 0.638569604086846 | 0.5108556832694832 | 0.12771392081737104 | 0.12771392081737104 | 0.638569604086846 | 0.383141762452107 | 0.383141762452107 | 0.12771392081737104 | 0.12771392081737104 | 1.021711366538953 | 0.893997445721584 | 0.638569604086846 | 0.12771392081737104 | 0.12771392081737104 | 0.0 | 0.0 | 0.12771392081737104 | 0.638569604086846 | 0.0 | 0.12771392081737104 | 0.0 | 0.12771392081737104 | 0.383141762452107 | 0.0 | 0.12771392081737104 | 0.0 | 0.383141762452107 | 0.383141762452107 | 0.12771392081737104 | 0.12771392081737104 | 1.021711366538953 | 0.12771392081737104 | 0.25542784163473803 | 0.383141762452107 | 0.12771392081737104 | 0.5108556832694832 | 0.25542784163473803 | 0.25542784163473803 | 0.12771392081737104 | 0.12771392081737104 | 0.0 | 0.12771392081737104 | 0.25542784163473803 | 0.0 | 0.5108556832694832 | 0.25542784163473803 | 0.12771392081737104 | 0.7662835249042149 | 0.12771392081737104 | 0.25542784163473803 |
| -32 | 1.1494252873563218 | 1.2771392081736788 | 0.7662835249042149 | 0.25542784163473803 | 1.021711366538953 | 0.12771392081737104 | 0.12771392081737104 | 0.12771392081737104 | 0.638569604086846 | 1.021711366538953 | 0.25542784163473803 | 0.25542784163473803 | 0.0 | 0.7662835249042149 | 0.0 | 0.12771392081737104 | 0.5108556832694832 | 1.021711366538953 | 0.12771392081737104 | 0.383141762452107 | 0.12771392081737104 | 0.12771392081737104 | 0.25542784163473803 | 0.0 | 0.0 | 0.0 | 0.638569604086846 | 1.021711366538953 | 0.12771392081737104 | 0.383141762452107 | 1.021711366538953 | 0.0 | 0.7662835249042149 | 0.0 | 0.0 | 0.383141762452107 | 0.0 | 0.893997445721584 | 0.25542784163473803 | 0.12771392081737104 | 0.25542784163473803 | 0.12771392081737104 | 0.12771392081737104 | 0.0 | 0.25542784163473803 | 0.12771392081737104 | 0.383141762452107 | 0.383141762452107 | 0.25542784163473803 | 0.383141762452107 |
| -31 | 2.298850574712643 | 1.53256704980843 | 0.638569604086846 | 0.12771392081737104 | 1.7879948914431536 | 0.12771392081737104 | 0.383141762452107 | 0.7662835249042149 | 0.5108556832694832 | 2.0434227330779065 | 0.5108556832694832 | 0.7662835249042149 | 0.383141762452107 | 0.638569604086846 | 0.12771392081737104 | 0.12771392081737104 | 0.25542784163473803 | 0.893997445721584 | 0.12771392081737104 | 0.0 | 0.12771392081737104 | 0.25542784163473803 | 0.25542784163473803 | 0.0 | 0.12771392081737104 | 0.0 | 0.638569604086846 | 0.7662835249042149 | 0.25542784163473803 | 0.25542784163473803 | 0.638569604086846 | 0.25542784163473803 | 0.5108556832694832 | 0.383141762452107 | 0.0 | 1.021711366538953 | 0.12771392081737104 | 1.53256704980843 | 0.25542784163473803 | 0.383141762452107 | 0.0 | 0.383141762452107 | 0.25542784163473803 | 0.0 | 0.383141762452107 | 0.12771392081737104 | 0.12771392081737104 | 0.383141762452107 | 0.25542784163473803 | 0.383141762452107 |
| -30 | 2.298850574712643 | 3.9591315453384452 | 1.6602809706258164 | 0.5108556832694832 | 1.6602809706258164 | 0.12771392081737104 | 1.021711366538953 | 0.638569604086846 | 0.893997445721584 | 1.1494252873563218 | 1.915708812260536 | 0.12771392081737104 | 0.7662835249042149 | 0.5108556832694832 | 0.383141762452107 | 0.25542784163473803 | 0.5108556832694832 | 0.5108556832694832 | 0.12771392081737104 | 0.25542784163473803 | 0.383141762452107 | 0.12771392081737104 | 0.25542784163473803 | 0.0 | 0.0 | 0.12771392081737104 | 0.7662835249042149 | 0.893997445721584 | 0.383141762452107 | 0.25542784163473803 | 0.638569604086846 | 0.5108556832694832 | 0.7662835249042149 | 0.25542784163473803 | 0.25542784163473803 | 0.383141762452107 | 0.0 | 0.638569604086846 | 0.12771392081737104 | 0.25542784163473803 | 0.12771392081737104 | 0.638569604086846 | 0.638569604086846 | 0.25542784163473803 | 0.7662835249042149 | 0.0 | 0.383141762452107 | 0.893997445721584 | 0.383141762452107 | 0.0 |
| -29 | 4.85312899106003 | 2.8097062579821404 | 1.915708812260536 | 0.638569604086846 | 1.1494252873563218 | 0.383141762452107 | 1.1494252873563218 | 1.021711366538953 | 2.298850574712643 | 1.7879948914431536 | 1.1494252873563218 | 0.5108556832694832 | 0.25542784163473803 | 1.2771392081736788 | 0.12771392081737104 | 1.021711366538953 | 0.5108556832694832 | 0.893997445721584 | 0.12771392081737104 | 0.12771392081737104 | 0.383141762452107 | 0.0 | 0.5108556832694832 | 0.7662835249042149 | 0.5108556832694832 | 0.12771392081737104 | 0.638569604086846 | 0.7662835249042149 | 0.25542784163473803 | 0.638569604086846 | 0.7662835249042149 | 0.0 | 0.5108556832694832 | 0.383141762452107 | 0.893997445721584 | 1.1494252873563218 | 0.25542784163473803 | 0.5108556832694832 | 0.25542784163473803 | 0.893997445721584 | 0.12771392081737104 | 0.7662835249042149 | 0.893997445721584 | 0.0 | 0.383141762452107 | 0.12771392081737104 | 0.12771392081737104 | 0.25542784163473803 | 0.12771392081737104 | 0.25542784163473803 |
| -28 | 3.5759897828863605 | 3.320561941251601 | 2.681992337164751 | 1.021711366538953 | 1.2771392081736788 | 1.1494252873563218 | 1.7879948914431536 | 1.2771392081736788 | 1.1494252873563218 | 1.021711366538953 | 1.021711366538953 | 0.25542784163473803 | 1.1494252873563218 | 1.021711366538953 | 0.383141762452107 | 0.5108556832694832 | 1.021711366538953 | 0.7662835249042149 | 0.25542784163473803 | 0.12771392081737104 | 1.1494252873563218 | 0.5108556832694832 | 0.0 | 0.0 | 0.638569604086846 | 0.12771392081737104 | 0.893997445721584 | 0.383141762452107 | 0.0 | 0.383141762452107 | 0.25542784163473803 | 0.12771392081737104 | 0.893997445721584 | 0.893997445721584 | 0.7662835249042149 | 0.25542784163473803 | 0.7662835249042149 | 0.25542784163473803 | 0.25542784163473803 | 0.0 | 0.12771392081737104 | 0.7662835249042149 | 0.12771392081737104 | 0.25542784163473803 | 0.893997445721584 | 0.383141762452107 | 0.5108556832694832 | 0.12771392081737104 | 0.383141762452107 | 0.25542784163473803 |
| -27 | 4.725415070242644 | 2.0434227330779065 | 2.171136653895275 | 2.8097062579821404 | 0.638569604086846 | 1.1494252873563218 | 1.1494252873563218 | 1.2771392081736788 | 0.7662835249042149 | 0.5108556832694832 | 0.893997445721584 | 0.5108556832694832 | 1.4048531289910702 | 1.021711366538953 | 0.7662835249042149 | 0.638569604086846 | 0.638569604086846 | 0.0 | 0.893997445721584 | 0.7662835249042149 | 0.638569604086846 | 0.5108556832694832 | 0.12771392081737104 | 0.383141762452107 | 0.25542784163473803 | 0.25542784163473803 | 0.638569604086846 | 0.383141762452107 | 0.383141762452107 | 0.5108556832694832 | 0.638569604086846 | 0.5108556832694832 | 0.7662835249042149 | 0.25542784163473803 | 0.893997445721584 | 0.25542784163473803 | 0.383141762452107 | 0.383141762452107 | 0.383141762452107 | 0.383141762452107 | 0.893997445721584 | 0.638569604086846 | 0.383141762452107 | 0.0 | 0.383141762452107 | 0.25542784163473803 | 0.383141762452107 | 0.383141762452107 | 0.25542784163473803 | 0.12771392081737104 |
| -26 | 2.937420178799489 | 1.53256704980843 | 2.298850574712643 | 1.4048531289910702 | 0.5108556832694832 | 1.53256704980843 | 0.7662835249042149 | 0.638569604086846 | 1.2771392081736788 | 0.5108556832694832 | 0.5108556832694832 | 0.893997445721584 | 1.1494252873563218 | 1.021711366538953 | 0.638569604086846 | 1.2771392081736788 | 0.5108556832694832 | 0.12771392081737104 | 1.1494252873563218 | 1.021711366538953 | 1.2771392081736788 | 0.5108556832694832 | 0.5108556832694832 | 0.5108556832694832 | 1.915708812260536 | 0.7662835249042149 | 0.25542784163473803 | 0.25542784163473803 | 0.7662835249042149 | 0.7662835249042149 | 0.12771392081737104 | 0.383141762452107 | 0.383141762452107 | 0.638569604086846 | 0.5108556832694832 | 0.383141762452107 | 0.383141762452107 | 0.12771392081737104 | 0.638569604086846 | 0.383141762452107 | 0.5108556832694832 | 0.638569604086846 | 0.383141762452107 | 0.383141762452107 | 0.383141762452107 | 0.5108556832694832 | 0.383141762452107 | 0.25542784163473803 | 0.638569604086846 | 0.25542784163473803 |
| -25 | 2.426564495530013 | 1.2771392081736788 | 1.7879948914431536 | 1.2771392081736788 | 0.25542784163473803 | 1.021711366538953 | 1.4048531289910702 | 1.2771392081736788 | 0.5108556832694832 | 0.383141762452107 | 0.25542784163473803 | 0.383141762452107 | 0.7662835249042149 | 0.5108556832694832 | 1.2771392081736788 | 0.638569604086846 | 1.021711366538953 | 0.383141762452107 | 0.7662835249042149 | 0.25542784163473803 | 0.638569604086846 | 0.7662835249042149 | 0.5108556832694832 | 0.5108556832694832 | 0.638569604086846 | 0.5108556832694832 | 0.383141762452107 | 0.25542784163473803 | 0.383141762452107 | 0.7662835249042149 | 0.12771392081737104 | 0.383141762452107 | 0.25542784163473803 | 0.383141762452107 | 0.25542784163473803 | 0.12771392081737104 | 0.638569604086846 | 0.0 | 0.7662835249042149 | 0.7662835249042149 | 1.1494252873563218 | 0.383141762452107 | 0.25542784163473803 | 0.638569604086846 | 0.12771392081737104 | 0.7662835249042149 | 0.383141762452107 | 0.0 | 0.25542784163473803 | 0.25542784163473803 |
| -24 | 1.7879948914431536 | 0.7662835249042149 | 0.893997445721584 | 0.638569604086846 | 0.638569604086846 | 0.638569604086846 | 0.7662835249042149 | 1.2771392081736788 | 0.383141762452107 | 0.0 | 0.25542784163473803 | 1.021711366538953 | 0.7662835249042149 | 0.0 | 0.7662835249042149 | 0.893997445721584 | 0.5108556832694832 | 0.25542784163473803 | 0.893997445721584 | 0.7662835249042149 | 0.5108556832694832 | 0.638569604086846 | 0.12771392081737104 | 0.638569604086846 | 0.5108556832694832 | 0.893997445721584 | 0.12771392081737104 | 0.12771392081737104 | 1.021711366538953 | 0.383141762452107 | 0.25542784163473803 | 0.638569604086846 | 0.0 | 0.25542784163473803 | 0.638569604086846 | 0.25542784163473803 | 0.383141762452107 | 0.0 | 0.383141762452107 | 0.25542784163473803 | 0.5108556832694832 | 0.12771392081737104 | 0.383141762452107 | 0.5108556832694832 | 0.5108556832694832 | 0.5108556832694832 | 1.021711366538953 | 0.0 | 0.383141762452107 | 0.893997445721584 |
| -23 | 1.021711366538953 | 0.7662835249042149 | 1.1494252873563218 | 1.021711366538953 | 0.383141762452107 | 1.2771392081736788 | 0.383141762452107 | 0.12771392081737104 | 0.5108556832694832 | 0.0 | 0.5108556832694832 | 1.2771392081736788 | 0.383141762452107 | 0.12771392081737104 | 0.638569604086846 | 0.638569604086846 | 0.638569604086846 | 0.638569604086846 | 0.638569604086846 | 0.638569604086846 | 0.638569604086846 | 1.1494252873563218 | 0.5108556832694832 | 0.5108556832694832 | 0.25542784163473803 | 0.25542784163473803 | 0.5108556832694832 | 0.0 | 0.638569604086846 | 0.5108556832694832 | 0.12771392081737104 | 0.7662835249042149 | 0.12771392081737104 | 0.5108556832694832 | 0.5108556832694832 | 0.5108556832694832 | 0.12771392081737104 | 0.0 | 0.638569604086846 | 0.383141762452107 | 0.5108556832694832 | 0.0 | 0.12771392081737104 | 1.021711366538953 | 0.12771392081737104 | 0.0 | 0.638569604086846 | 0.383141762452107 | 0.383141762452107 | 0.383141762452107 |
| -22 | 0.7662835249042149 | 1.021711366538953 | 0.383141762452107 | 1.021711366538953 | 0.12771392081737104 | 1.1494252873563218 | 0.638569604086846 | 0.638569604086846 | 0.383141762452107 | 0.0 | 0.383141762452107 | 0.5108556832694832 | 0.5108556832694832 | 0.383141762452107 | 0.7662835249042149 | 0.12771392081737104 | 0.5108556832694832 | 0.5108556832694832 | 0.638569604086846 | 0.638569604086846 | 0.5108556832694832 | 0.383141762452107 | 0.5108556832694832 | 0.893997445721584 | 0.25542784163473803 | 0.7662835249042149 | 0.25542784163473803 | 0.0 | 0.638569604086846 | 0.12771392081737104 | 0.12771392081737104 | 0.7662835249042149 | 0.12771392081737104 | 0.383141762452107 | 0.383141762452107 | 0.12771392081737104 | 0.383141762452107 | 0.25542784163473803 | 0.383141762452107 | 0.7662835249042149 | 0.25542784163473803 | 0.12771392081737104 | 0.383141762452107 | 0.383141762452107 | 0.25542784163473803 | 0.383141762452107 | 0.25542784163473803 | 0.383141762452107 | 0.638569604086846 | 0.12771392081737104 |
| -21 | 1.4048531289910702 | 0.25542784163473803 | 0.638569604086846 | 0.7662835249042149 | 0.0 | 1.021711366538953 | 0.638569604086846 | 0.638569604086846 | 0.5108556832694832 | 0.0 | 0.25542784163473803 | 0.383141762452107 | 0.25542784163473803 | 0.25542784163473803 | 1.021711366538953 | 0.25542784163473803 | 0.5108556832694832 | 0.0 | 0.5108556832694832 | 0.5108556832694832 | 0.12771392081737104 | 0.25542784163473803 | 0.5108556832694832 | 0.7662835249042149 | 0.383141762452107 | 0.5108556832694832 | 0.0 | 0.12771392081737104 | 0.383141762452107 | 0.12771392081737104 | 0.12771392081737104 | 0.5108556832694832 | 0.0 | 0.12771392081737104 | 0.0 | 0.12771392081737104 | 0.7662835249042149 | 0.0 | 0.12771392081737104 | 0.383141762452107 | 0.5108556832694832 | 0.25542784163473803 | 0.383141762452107 | 0.383141762452107 | 0.12771392081737104 | 0.12771392081737104 | 0.12771392081737104 | 0.0 | 0.25542784163473803 | 0.383141762452107 |
| -20 | 0.25542784163473803 | 0.0 | 0.638569604086846 | 0.638569604086846 | 0.25542784163473803 | 0.383141762452107 | 0.12771392081737104 | 0.383141762452107 | 0.25542784163473803 | 0.0 | 0.12771392081737104 | 0.383141762452107 | 0.5108556832694832 | 0.12771392081737104 | 0.638569604086846 | 0.638569604086846 | 0.0 | 0.12771392081737104 | 0.5108556832694832 | 0.383141762452107 | 0.25542784163473803 | 0.25542784163473803 | 0.893997445721584 | 0.5108556832694832 | 0.25542784163473803 | 0.893997445721584 | 0.0 | 0.12771392081737104 | 0.12771392081737104 | 0.5108556832694832 | 0.12771392081737104 | 0.638569604086846 | 0.12771392081737104 | 0.383141762452107 | 0.12771392081737104 | 0.0 | 0.5108556832694832 | 0.0 | 0.383141762452107 | 0.12771392081737104 | 0.12771392081737104 | 0.12771392081737104 | 0.25542784163473803 | 0.12771392081737104 | 0.12771392081737104 | 0.5108556832694832 | 0.0 | 0.0 | 0.12771392081737104 | 0.5108556832694832 |
| -19 | 0.25542784163473803 | 0.12771392081737104 | 0.383141762452107 | 0.25542784163473803 | 0.12771392081737104 | 0.5108556832694832 | 0.25542784163473803 | 0.5108556832694832 | 0.12771392081737104 | 0.0 | 0.25542784163473803 | 0.7662835249042149 | 0.12771392081737104 | 0.0 | 0.638569604086846 | 0.638569604086846 | 0.12771392081737104 | 0.12771392081737104 | 0.383141762452107 | 0.5108556832694832 | 0.0 | 0.893997445721584 | 0.25542784163473803 | 0.7662835249042149 | 0.383141762452107 | 0.7662835249042149 | 0.0 | 0.0 | 0.25542784163473803 | 0.383141762452107 | 0.0 | 0.383141762452107 | 0.0 | 0.12771392081737104 | 0.0 | 0.0 | 0.383141762452107 | 0.12771392081737104 | 0.5108556832694832 | 0.25542784163473803 | 0.12771392081737104 | 0.0 | 0.12771392081737104 | 0.893997445721584 | 0.0 | 0.5108556832694832 | 0.0 | 0.25542784163473803 | 0.383141762452107 | 0.25542784163473803 |D Ostreococcus lucimarinus (Ostreococcus)
E Cyanidioschyzon merolae (Red alga)
### Chart
| Category | UAAA | GUAA | AAAC | AAAG | AAAA | AUAA | AAAU | CGUA | AACA | AAGC | AAGU | AACG | AACU | AAUG | ACGU | CGAA | CUAA | AUCG | AAGA | UCGU | CACG | AGUA | CGUU | GGAA | UAUA | GAAC | GAAA | CAUA | ACUG | ACAU | CAUC | GCGG | UGUA | ACAG | AUGC | UGAU | ACGC | AAUA | UGCC | UCGA | ACAC | GUUU | AGCG | CCGU | GACG | CUGU | GAUA | UCAU | AGGA | UUUU |
|---|---|---|---|---|---|---|---|---|---|---|---|---|---|---|---|---|---|---|---|---|---|---|---|---|---|---|---|---|---|---|---|---|---|---|---|---|---|---|---|---|---|---|---|---|---|---|---|---|---|---|
| -30 | 1.9354838709677502 | 0.6451612903225862 | 0.6451612903225862 | 1.290322580645161 | 0.0 | 1.9354838709677502 | 0.6451612903225862 | 1.290322580645161 | 1.290322580645161 | 1.290322580645161 | 0.6451612903225862 | 0.0 | 1.290322580645161 | 0.6451612903225862 | 0.0 | 1.290322580645161 | 0.6451612903225862 | 1.9354838709677502 | 0.0 | 1.290322580645161 | 1.290322580645161 | 0.0 | 0.6451612903225862 | 1.9354838709677502 | 0.0 | 0.0 | 0.6451612903225862 | 0.6451612903225862 | 0.0 | 0.0 | 0.6451612903225862 | 0.0 | 0.6451612903225862 | 0.6451612903225862 | 0.0 | 1.9354838709677502 | 1.290322580645161 | 0.0 | 0.0 | 1.9354838709677502 | 0.6451612903225862 | 0.6451612903225862 | 0.6451612903225862 | 0.6451612903225862 | 1.9354838709677502 | 0.6451612903225862 | 1.9354838709677502 | 0.0 | 0.0 | 0.6451612903225862 |
| -29 | 3.2258064516129052 | 1.290322580645161 | 0.0 | 1.290322580645161 | 0.6451612903225862 | 1.290322580645161 | 0.6451612903225862 | 0.6451612903225862 | 0.0 | 1.9354838709677502 | 0.0 | 0.6451612903225862 | 1.290322580645161 | 0.0 | 1.290322580645161 | 0.6451612903225862 | 0.6451612903225862 | 0.6451612903225862 | 0.0 | 0.6451612903225862 | 1.290322580645161 | 0.6451612903225862 | 0.6451612903225862 | 1.290322580645161 | 0.0 | 0.6451612903225862 | 1.290322580645161 | 0.6451612903225862 | 0.6451612903225862 | 0.6451612903225862 | 0.6451612903225862 | 0.6451612903225862 | 0.0 | 0.6451612903225862 | 2.580645161290322 | 0.0 | 1.9354838709677502 | 0.0 | 0.0 | 1.290322580645161 | 0.0 | 0.0 | 0.6451612903225862 | 0.6451612903225862 | 0.0 | 0.0 | 0.6451612903225862 | 1.290322580645161 | 0.0 | 1.290322580645161 |
| -28 | 3.2258064516129052 | 1.290322580645161 | 1.9354838709677502 | 1.290322580645161 | 0.6451612903225862 | 0.6451612903225862 | 1.290322580645161 | 1.9354838709677502 | 0.0 | 0.0 | 0.0 | 0.6451612903225862 | 0.0 | 1.290322580645161 | 1.290322580645161 | 1.9354838709677502 | 0.6451612903225862 | 1.290322580645161 | 1.290322580645161 | 0.0 | 0.6451612903225862 | 0.0 | 0.6451612903225862 | 0.0 | 0.6451612903225862 | 0.6451612903225862 | 1.290322580645161 | 0.6451612903225862 | 0.0 | 0.0 | 0.6451612903225862 | 0.0 | 0.6451612903225862 | 0.0 | 0.0 | 0.0 | 0.0 | 0.6451612903225862 | 1.9354838709677502 | 1.290322580645161 | 1.290322580645161 | 0.6451612903225862 | 1.290322580645161 | 0.0 | 1.290322580645161 | 0.6451612903225862 | 0.0 | 0.0 | 1.290322580645161 | 0.6451612903225862 |
| -27 | 2.580645161290322 | 2.580645161290322 | 1.290322580645161 | 2.580645161290322 | 1.9354838709677502 | 0.6451612903225862 | 0.0 | 1.9354838709677502 | 1.9354838709677502 | 1.9354838709677502 | 0.6451612903225862 | 0.0 | 0.6451612903225862 | 0.6451612903225862 | 0.6451612903225862 | 1.9354838709677502 | 0.0 | 0.6451612903225862 | 0.0 | 1.290322580645161 | 1.290322580645161 | 0.0 | 0.6451612903225862 | 1.290322580645161 | 0.6451612903225862 | 0.6451612903225862 | 0.0 | 0.0 | 0.0 | 0.6451612903225862 | 1.290322580645161 | 2.580645161290322 | 1.290322580645161 | 0.0 | 0.6451612903225862 | 0.0 | 0.6451612903225862 | 0.6451612903225862 | 0.0 | 0.6451612903225862 | 0.6451612903225862 | 1.290322580645161 | 1.290322580645161 | 0.0 | 0.0 | 0.0 | 0.0 | 2.580645161290322 | 1.9354838709677502 | 0.6451612903225862 |
| -26 | 3.2258064516129052 | 3.2258064516129052 | 1.290322580645161 | 2.580645161290322 | 0.6451612903225862 | 0.6451612903225862 | 0.0 | 1.290322580645161 | 0.0 | 0.6451612903225862 | 1.290322580645161 | 0.0 | 1.290322580645161 | 0.6451612903225862 | 1.290322580645161 | 1.290322580645161 | 0.6451612903225862 | 1.290322580645161 | 0.0 | 0.6451612903225862 | 1.290322580645161 | 1.290322580645161 | 0.6451612903225862 | 0.6451612903225862 | 0.6451612903225862 | 0.6451612903225862 | 1.290322580645161 | 0.6451612903225862 | 0.6451612903225862 | 0.6451612903225862 | 1.9354838709677502 | 0.6451612903225862 | 0.6451612903225862 | 0.6451612903225862 | 0.0 | 0.6451612903225862 | 0.0 | 1.290322580645161 | 0.6451612903225862 | 0.6451612903225862 | 1.290322580645161 | 0.0 | 1.290322580645161 | 1.290322580645161 | 1.9354838709677502 | 0.0 | 0.0 | 0.6451612903225862 | 0.0 | 0.0 |
| -25 | 4.51612903225807 | 2.580645161290322 | 1.290322580645161 | 1.290322580645161 | 0.6451612903225862 | 1.290322580645161 | 1.9354838709677502 | 3.2258064516129052 | 0.0 | 0.0 | 1.290322580645161 | 1.290322580645161 | 0.0 | 0.6451612903225862 | 2.580645161290322 | 0.6451612903225862 | 0.6451612903225862 | 0.0 | 1.290322580645161 | 1.290322580645161 | 1.290322580645161 | 0.6451612903225862 | 0.6451612903225862 | 0.6451612903225862 | 1.290322580645161 | 0.6451612903225862 | 0.0 | 1.290322580645161 | 0.6451612903225862 | 0.0 | 0.0 | 0.6451612903225862 | 0.6451612903225862 | 0.6451612903225862 | 1.290322580645161 | 0.0 | 0.0 | 0.0 | 0.0 | 0.6451612903225862 | 0.0 | 0.6451612903225862 | 0.6451612903225862 | 0.6451612903225862 | 0.6451612903225862 | 0.0 | 0.0 | 0.6451612903225862 | 0.0 | 0.0 |
| -24 | 5.80645161290323 | 5.80645161290323 | 1.290322580645161 | 1.290322580645161 | 1.290322580645161 | 2.580645161290322 | 1.290322580645161 | 3.2258064516129052 | 1.290322580645161 | 0.6451612903225862 | 0.6451612903225862 | 0.0 | 0.6451612903225862 | 0.6451612903225862 | 1.9354838709677502 | 1.290322580645161 | 0.6451612903225862 | 0.0 | 0.6451612903225862 | 1.9354838709677502 | 0.6451612903225862 | 1.290322580645161 | 0.6451612903225862 | 0.6451612903225862 | 1.290322580645161 | 0.0 | 0.6451612903225862 | 0.6451612903225862 | 0.0 | 0.6451612903225862 | 0.0 | 0.6451612903225862 | 1.290322580645161 | 0.0 | 0.0 | 0.6451612903225862 | 0.0 | 1.9354838709677502 | 1.290322580645161 | 0.0 | 0.0 | 0.0 | 0.0 | 1.9354838709677502 | 0.0 | 0.0 | 0.6451612903225862 | 0.6451612903225862 | 1.290322580645161 | 1.290322580645161 |
| -23 | 9.032258064516107 | 5.16129032258065 | 3.2258064516129052 | 1.290322580645161 | 1.9354838709677502 | 4.51612903225807 | 1.290322580645161 | 3.8709677419355013 | 0.0 | 0.6451612903225862 | 0.6451612903225862 | 0.6451612903225862 | 0.0 | 0.6451612903225862 | 0.6451612903225862 | 0.6451612903225862 | 2.580645161290322 | 0.0 | 0.0 | 0.0 | 0.6451612903225862 | 0.6451612903225862 | 0.6451612903225862 | 0.0 | 0.0 | 0.6451612903225862 | 1.9354838709677502 | 0.6451612903225862 | 0.6451612903225862 | 0.6451612903225862 | 0.0 | 0.0 | 0.0 | 0.0 | 0.0 | 0.0 | 0.0 | 1.290322580645161 | 0.0 | 0.0 | 0.0 | 0.6451612903225862 | 0.0 | 1.290322580645161 | 0.0 | 0.6451612903225862 | 0.6451612903225862 | 0.6451612903225862 | 0.0 | 1.9354838709677502 |
| -22 | 12.90322580645161 | 4.51612903225807 | 2.580645161290322 | 3.8709677419355013 | 3.8709677419355013 | 1.9354838709677502 | 1.290322580645161 | 1.9354838709677502 | 1.9354838709677502 | 0.0 | 1.290322580645161 | 1.290322580645161 | 0.6451612903225862 | 0.6451612903225862 | 0.6451612903225862 | 0.0 | 2.580645161290322 | 0.0 | 0.0 | 0.6451612903225862 | 0.6451612903225862 | 0.6451612903225862 | 0.0 | 0.0 | 1.290322580645161 | 0.0 | 0.0 | 1.290322580645161 | 0.6451612903225862 | 1.290322580645161 | 0.0 | 1.290322580645161 | 0.6451612903225862 | 0.0 | 0.0 | 0.0 | 0.0 | 0.6451612903225862 | 0.0 | 0.0 | 0.0 | 0.0 | 0.0 | 1.9354838709677502 | 0.6451612903225862 | 0.6451612903225862 | 0.6451612903225862 | 0.0 | 0.0 | 0.0 |
| -21 | 10.322580645161382 | 6.451612903225811 | 3.2258064516129052 | 5.80645161290323 | 4.51612903225807 | 3.2258064516129052 | 3.2258064516129052 | 1.290322580645161 | 0.6451612903225862 | 1.9354838709677502 | 1.9354838709677502 | 1.290322580645161 | 0.6451612903225862 | 0.0 | 0.0 | 0.6451612903225862 | 1.290322580645161 | 0.6451612903225862 | 0.6451612903225862 | 0.0 | 0.0 | 0.6451612903225862 | 0.6451612903225862 | 0.0 | 1.290322580645161 | 0.0 | 0.0 | 1.290322580645161 | 0.0 | 1.9354838709677502 | 0.0 | 0.0 | 1.9354838709677502 | 1.290322580645161 | 0.6451612903225862 | 0.6451612903225862 | 0.6451612903225862 | 0.6451612903225862 | 0.0 | 0.6451612903225862 | 0.6451612903225862 | 0.0 | 0.0 | 0.0 | 0.0 | 0.6451612903225862 | 0.6451612903225862 | 0.0 | 0.0 | 0.6451612903225862 |
| -20 | 12.258064516129082 | 3.8709677419355013 | 5.16129032258065 | 5.16129032258065 | 1.9354838709677502 | 3.2258064516129052 | 1.9354838709677502 | 0.6451612903225862 | 1.290322580645161 | 3.2258064516129052 | 1.9354838709677502 | 0.0 | 1.9354838709677502 | 1.290322580645161 | 0.6451612903225862 | 0.0 | 0.0 | 0.0 | 0.6451612903225862 | 0.6451612903225862 | 0.0 | 1.9354838709677502 | 0.0 | 0.6451612903225862 | 1.290322580645161 | 0.0 | 0.6451612903225862 | 0.0 | 0.0 | 0.0 | 1.290322580645161 | 0.6451612903225862 | 1.290322580645161 | 1.290322580645161 | 0.0 | 0.0 | 0.0 | 0.6451612903225862 | 0.0 | 0.6451612903225862 | 0.0 | 0.0 | 0.6451612903225862 | 0.0 | 0.0 | 0.0 | 0.6451612903225862 | 0.0 | 0.0 | 0.0 |
| -19 | 7.096774193548392 | 3.8709677419355013 | 5.80645161290323 | 5.16129032258065 | 3.8709677419355013 | 1.9354838709677502 | 2.580645161290322 | 1.290322580645161 | 0.6451612903225862 | 3.2258064516129052 | 0.6451612903225862 | 3.2258064516129052 | 0.0 | 1.290322580645161 | 0.0 | 0.0 | 1.290322580645161 | 0.6451612903225862 | 1.290322580645161 | 0.0 | 0.0 | 1.290322580645161 | 0.6451612903225862 | 0.6451612903225862 | 0.0 | 0.6451612903225862 | 0.0 | 0.0 | 1.290322580645161 | 0.6451612903225862 | 0.0 | 0.6451612903225862 | 0.0 | 0.0 | 0.6451612903225862 | 0.0 | 0.0 | 0.0 | 0.6451612903225862 | 0.0 | 0.0 | 0.0 | 1.290322580645161 | 0.0 | 0.0 | 0.0 | 0.6451612903225862 | 0.6451612903225862 | 0.6451612903225862 | 0.0 |
| -18 | 8.38709677419355 | 1.9354838709677502 | 6.451612903225811 | 1.290322580645161 | 1.290322580645161 | 0.0 | 2.580645161290322 | 0.0 | 3.2258064516129052 | 1.9354838709677502 | 1.9354838709677502 | 1.290322580645161 | 1.9354838709677502 | 2.580645161290322 | 1.290322580645161 | 0.0 | 0.6451612903225862 | 0.0 | 1.9354838709677502 | 0.6451612903225862 | 0.0 | 0.0 | 0.6451612903225862 | 0.0 | 0.6451612903225862 | 0.6451612903225862 | 0.0 | 0.0 | 0.0 | 0.0 | 1.290322580645161 | 0.6451612903225862 | 0.0 | 0.6451612903225862 | 1.290322580645161 | 0.6451612903225862 | 0.6451612903225862 | 0.0 | 0.0 | 0.0 | 0.6451612903225862 | 0.6451612903225862 | 1.290322580645161 | 0.0 | 0.0 | 0.6451612903225862 | 0.0 | 0.6451612903225862 | 0.6451612903225862 | 0.0 |
| -17 | 2.580645161290322 | 0.0 | 3.2258064516129052 | 3.2258064516129052 | 1.9354838709677502 | 0.6451612903225862 | 1.290322580645161 | 0.0 | 1.290322580645161 | 0.0 | 0.6451612903225862 | 2.580645161290322 | 1.9354838709677502 | 0.6451612903225862 | 0.0 | 1.290322580645161 | 0.0 | 1.290322580645161 | 1.290322580645161 | 0.0 | 0.0 | 0.0 | 1.290322580645161 | 0.6451612903225862 | 0.0 | 0.0 | 0.0 | 0.0 | 0.6451612903225862 | 1.290322580645161 | 0.0 | 1.290322580645161 | 0.6451612903225862 | 1.290322580645161 | 1.290322580645161 | 1.290322580645161 | 0.6451612903225862 | 1.290322580645161 | 1.290322580645161 | 0.0 | 2.580645161290322 | 0.0 | 0.0 | 0.0 | 0.0 | 0.0 | 0.0 | 0.6451612903225862 | 0.6451612903225862 | 0.0 |
| -16 | 0.6451612903225862 | 0.0 | 1.290322580645161 | 0.6451612903225862 | 1.290322580645161 | 0.0 | 1.290322580645161 | 0.0 | 1.9354838709677502 | 0.0 | 1.9354838709677502 | 1.290322580645161 | 0.0 | 0.6451612903225862 | 0.6451612903225862 | 0.6451612903225862 | 0.0 | 0.0 | 0.0 | 0.6451612903225862 | 1.290322580645161 | 0.0 | 0.0 | 1.290322580645161 | 0.0 | 3.2258064516129052 | 1.290322580645161 | 0.6451612903225862 | 1.9354838709677502 | 0.6451612903225862 | 1.290322580645161 | 0.6451612903225862 | 0.0 | 0.0 | 0.6451612903225862 | 0.6451612903225862 | 0.6451612903225862 | 0.6451612903225862 | 1.290322580645161 | 0.0 | 0.6451612903225862 | 1.290322580645161 | 0.0 | 0.6451612903225862 | 0.6451612903225862 | 1.290322580645161 | 0.6451612903225862 | 0.0 | 0.6451612903225862 | 0.0 |
| -15 | 0.0 | 0.0 | 0.6451612903225862 | 1.9354838709677502 | 0.0 | 0.6451612903225862 | 0.6451612903225862 | 0.0 | 1.9354838709677502 | 0.0 | 0.0 | 0.6451612903225862 | 1.290322580645161 | 1.290322580645161 | 0.6451612903225862 | 1.290322580645161 | 0.6451612903225862 | 1.290322580645161 | 0.6451612903225862 | 0.0 | 0.0 | 0.6451612903225862 | 1.290322580645161 | 0.0 | 0.0 | 0.6451612903225862 | 1.290322580645161 | 0.6451612903225862 | 0.0 | 0.6451612903225862 | 0.0 | 0.0 | 0.0 | 1.290322580645161 | 0.6451612903225862 | 1.290322580645161 | 1.290322580645161 | 0.0 | 1.9354838709677502 | 0.6451612903225862 | 0.0 | 0.6451612903225862 | 0.0 | 0.0 | 1.290322580645161 | 0.0 | 1.290322580645161 | 0.6451612903225862 | 0.0 | 0.6451612903225862 |
| -14 | 0.6451612903225862 | 0.6451612903225862 | 0.6451612903225862 | 0.0 | 0.6451612903225862 | 0.6451612903225862 | 0.6451612903225862 | 0.0 | 0.6451612903225862 | 0.6451612903225862 | 0.6451612903225862 | 1.290322580645161 | 1.290322580645161 | 0.6451612903225862 | 0.6451612903225862 | 0.0 | 0.0 | 0.6451612903225862 | 1.290322580645161 | 1.290322580645161 | 0.0 | 0.6451612903225862 | 0.6451612903225862 | 0.6451612903225862 | 0.0 | 0.6451612903225862 | 0.0 | 0.6451612903225862 | 1.9354838709677502 | 0.6451612903225862 | 0.6451612903225862 | 0.0 | 0.0 | 0.6451612903225862 | 0.0 | 0.6451612903225862 | 1.290322580645161 | 0.0 | 0.6451612903225862 | 0.6451612903225862 | 1.290322580645161 | 1.9354838709677502 | 0.6451612903225862 | 0.0 | 0.0 | 0.0 | 0.0 | 0.0 | 1.290322580645161 | 0.6451612903225862 |
| -13 | 0.0 | 0.0 | 1.290322580645161 | 1.290322580645161 | 0.0 | 0.0 | 0.0 | 0.0 | 0.6451612903225862 | 0.0 | 0.0 | 0.0 | 0.6451612903225862 | 0.6451612903225862 | 0.0 | 0.0 | 0.0 | 1.290322580645161 | 0.6451612903225862 | 0.6451612903225862 | 0.6451612903225862 | 0.6451612903225862 | 0.6451612903225862 | 0.6451612903225862 | 1.290322580645161 | 0.6451612903225862 | 0.0 | 0.6451612903225862 | 1.290322580645161 | 0.0 | 0.6451612903225862 | 0.0 | 0.0 | 0.6451612903225862 | 0.0 | 1.290322580645161 | 0.6451612903225862 | 0.0 | 0.0 | 0.6451612903225862 | 0.0 | 1.290322580645161 | 0.0 | 0.6451612903225862 | 0.6451612903225862 | 3.8709677419355013 | 0.6451612903225862 | 0.0 | 0.6451612903225862 | 0.6451612903225862 |
### Chart
| Category | AAUAAA | AAAUAA | AAAGAA | AUAAAA | AAGAAA | AAAAAA | UAAAAA | UAAUAA | AAAAGA | AAAAAU | AGAAAA | AAUAUA | GAAAAA | AAAAAG | UAUAAA | UAAAUA | AAAAUA | AUAAUA | AUAAAU | AAUAAU | AAACAA | AUCAAA | AUAUAU | GAAUAA | UAUAUA | AAUCAA | UUUAAA | AUAUAA | AACAAA | AAUGAA | AUAAAG | UUUUAA | UUAAAA | AAAAAC | GAAAGA | UAAAAU | AAAUUU | AAAUAU | AAGAAU | AAUAAG | AAAAUU | CAAUAA | UUUAAU | UUAAAU | AUCAAU | AUGAAA | GAAAUA | UAAAAG | GAAGAA | UAAAGA |
|---|---|---|---|---|---|---|---|---|---|---|---|---|---|---|---|---|---|---|---|---|---|---|---|---|---|---|---|---|---|---|---|---|---|---|---|---|---|---|---|---|---|---|---|---|---|---|---|---|---|---|
| -32 | 0.17621145374449607 | 0.19823788546255502 | 0.275330396475771 | 0.187224669603524 | 0.231277533039648 | 0.13215859030837 | 0.13215859030837 | 0.187224669603524 | 0.17621145374449607 | 0.13215859030837 | 0.09911894273127872 | 0.13215859030837 | 0.187224669603524 | 0.14317180616740102 | 0.14317180616740102 | 0.12114537444934001 | 0.066079295154185 | 0.16519823788546606 | 0.07709251101321722 | 0.07709251101321722 | 0.19823788546255502 | 0.13215859030837 | 0.08810572687224673 | 0.055066079295154204 | 0.231277533039648 | 0.15418502202643203 | 0.187224669603524 | 0.15418502202643203 | 0.15418502202643203 | 0.13215859030837 | 0.07709251101321722 | 0.19823788546255502 | 0.12114537444934001 | 0.08810572687224673 | 0.15418502202643203 | 0.055066079295154204 | 0.220264317180617 | 0.07709251101321722 | 0.14317180616740102 | 0.09911894273127872 | 0.11013215859030799 | 0.066079295154185 | 0.13215859030837 | 0.07709251101321722 | 0.08810572687224673 | 0.17621145374449607 | 0.11013215859030799 | 0.11013215859030799 | 0.16519823788546606 | 0.07709251101321722 |
| -31 | 0.2973568281938361 | 0.15418502202643203 | 0.26431718061674003 | 0.14317180616740102 | 0.26431718061674003 | 0.220264317180617 | 0.15418502202643203 | 0.14317180616740102 | 0.19823788546255502 | 0.231277533039648 | 0.209251101321586 | 0.13215859030837 | 0.209251101321586 | 0.13215859030837 | 0.220264317180617 | 0.14317180616740102 | 0.13215859030837 | 0.07709251101321722 | 0.14317180616740102 | 0.13215859030837 | 0.12114537444934001 | 0.15418502202643203 | 0.13215859030837 | 0.11013215859030799 | 0.14317180616740102 | 0.13215859030837 | 0.17621145374449607 | 0.220264317180617 | 0.17621145374449607 | 0.09911894273127872 | 0.055066079295154204 | 0.209251101321586 | 0.15418502202643203 | 0.066079295154185 | 0.13215859030837 | 0.13215859030837 | 0.08810572687224673 | 0.15418502202643203 | 0.16519823788546606 | 0.022026431718061706 | 0.11013215859030799 | 0.04405286343612352 | 0.220264317180617 | 0.11013215859030799 | 0.19823788546255502 | 0.12114537444934001 | 0.066079295154185 | 0.08810572687224673 | 0.055066079295154204 | 0.08810572687224673 |
| -30 | 0.209251101321586 | 0.231277533039648 | 0.3414096916299611 | 0.2863436123348071 | 0.220264317180617 | 0.12114537444934001 | 0.19823788546255502 | 0.16519823788546606 | 0.17621145374449607 | 0.24229074889867802 | 0.19823788546255502 | 0.15418502202643203 | 0.16519823788546606 | 0.08810572687224673 | 0.220264317180617 | 0.187224669603524 | 0.19823788546255502 | 0.15418502202643203 | 0.16519823788546606 | 0.14317180616740102 | 0.17621145374449607 | 0.13215859030837 | 0.14317180616740102 | 0.187224669603524 | 0.19823788546255502 | 0.11013215859030799 | 0.220264317180617 | 0.09911894273127872 | 0.055066079295154204 | 0.13215859030837 | 0.14317180616740102 | 0.25330396475771205 | 0.16519823788546606 | 0.16519823788546606 | 0.11013215859030799 | 0.07709251101321722 | 0.09911894273127872 | 0.14317180616740102 | 0.14317180616740102 | 0.04405286343612352 | 0.220264317180617 | 0.11013215859030799 | 0.187224669603524 | 0.19823788546255502 | 0.12114537444934001 | 0.15418502202643203 | 0.09911894273127872 | 0.11013215859030799 | 0.12114537444934001 | 0.09911894273127872 |
| -29 | 0.3744493392070531 | 0.3414096916299611 | 0.26431718061674003 | 0.15418502202643203 | 0.3744493392070531 | 0.209251101321586 | 0.24229074889867802 | 0.24229074889867802 | 0.24229074889867802 | 0.17621145374449607 | 0.15418502202643203 | 0.19823788546255502 | 0.11013215859030799 | 0.15418502202643203 | 0.16519823788546606 | 0.26431718061674003 | 0.24229074889867802 | 0.16519823788546606 | 0.24229074889867802 | 0.15418502202643203 | 0.15418502202643203 | 0.17621145374449607 | 0.16519823788546606 | 0.26431718061674003 | 0.17621145374449607 | 0.209251101321586 | 0.24229074889867802 | 0.16519823788546606 | 0.187224669603524 | 0.066079295154185 | 0.055066079295154204 | 0.220264317180617 | 0.15418502202643203 | 0.08810572687224673 | 0.187224669603524 | 0.17621145374449607 | 0.15418502202643203 | 0.17621145374449607 | 0.15418502202643203 | 0.15418502202643203 | 0.055066079295154204 | 0.13215859030837 | 0.17621145374449607 | 0.13215859030837 | 0.220264317180617 | 0.14317180616740102 | 0.187224669603524 | 0.17621145374449607 | 0.13215859030837 | 0.07709251101321722 |
| -28 | 0.506607929515419 | 0.451541850220264 | 0.38546255506608307 | 0.26431718061674003 | 0.3414096916299611 | 0.16519823788546606 | 0.187224669603524 | 0.209251101321586 | 0.26431718061674003 | 0.26431718061674003 | 0.2973568281938361 | 0.209251101321586 | 0.24229074889867802 | 0.07709251101321722 | 0.17621145374449607 | 0.2863436123348071 | 0.231277533039648 | 0.25330396475771205 | 0.25330396475771205 | 0.25330396475771205 | 0.24229074889867802 | 0.17621145374449607 | 0.16519823788546606 | 0.3083700440528691 | 0.15418502202643203 | 0.15418502202643203 | 0.17621145374449607 | 0.220264317180617 | 0.187224669603524 | 0.24229074889867802 | 0.15418502202643203 | 0.187224669603524 | 0.187224669603524 | 0.13215859030837 | 0.17621145374449607 | 0.12114537444934001 | 0.07709251101321722 | 0.209251101321586 | 0.187224669603524 | 0.16519823788546606 | 0.11013215859030799 | 0.17621145374449607 | 0.275330396475771 | 0.15418502202643203 | 0.19823788546255502 | 0.08810572687224673 | 0.12114537444934001 | 0.09911894273127872 | 0.11013215859030799 | 0.11013215859030799 |
| -27 | 0.583700440528634 | 0.36343612334802006 | 0.4845814977973621 | 0.3303964757709311 | 0.36343612334802006 | 0.275330396475771 | 0.24229074889867802 | 0.3524229074889901 | 0.12114537444934001 | 0.13215859030837 | 0.2863436123348071 | 0.26431718061674003 | 0.220264317180617 | 0.13215859030837 | 0.220264317180617 | 0.25330396475771205 | 0.187224669603524 | 0.3083700440528691 | 0.231277533039648 | 0.2863436123348071 | 0.15418502202643203 | 0.17621145374449607 | 0.19823788546255502 | 0.275330396475771 | 0.3083700440528691 | 0.26431718061674003 | 0.09911894273127872 | 0.16519823788546606 | 0.24229074889867802 | 0.25330396475771205 | 0.16519823788546606 | 0.3964757709251151 | 0.12114537444934001 | 0.14317180616740102 | 0.14317180616740102 | 0.12114537444934001 | 0.12114537444934001 | 0.209251101321586 | 0.13215859030837 | 0.16519823788546606 | 0.16519823788546606 | 0.14317180616740102 | 0.187224669603524 | 0.14317180616740102 | 0.19823788546255502 | 0.17621145374449607 | 0.209251101321586 | 0.15418502202643203 | 0.187224669603524 | 0.12114537444934001 |
| -26 | 0.5726872246696041 | 0.46255506607929503 | 0.3193832599118981 | 0.40748898678414713 | 0.451541850220264 | 0.6167400881057331 | 0.25330396475771205 | 0.418502202643172 | 0.231277533039648 | 0.25330396475771205 | 0.24229074889867802 | 0.19823788546255502 | 0.17621145374449607 | 0.209251101321586 | 0.14317180616740102 | 0.19823788546255502 | 0.209251101321586 | 0.26431718061674003 | 0.275330396475771 | 0.24229074889867802 | 0.19823788546255502 | 0.209251101321586 | 0.3193832599118981 | 0.3193832599118981 | 0.231277533039648 | 0.209251101321586 | 0.3083700440528691 | 0.24229074889867802 | 0.209251101321586 | 0.24229074889867802 | 0.17621145374449607 | 0.19823788546255502 | 0.17621145374449607 | 0.16519823788546606 | 0.13215859030837 | 0.16519823788546606 | 0.19823788546255502 | 0.11013215859030799 | 0.16519823788546606 | 0.15418502202643203 | 0.09911894273127872 | 0.209251101321586 | 0.231277533039648 | 0.16519823788546606 | 0.19823788546255502 | 0.187224669603524 | 0.15418502202643203 | 0.08810572687224673 | 0.187224669603524 | 0.12114537444934001 |
| -25 | 0.7709251101321651 | 0.4845814977973621 | 0.3303964757709311 | 0.47356828193832906 | 0.38546255506608307 | 0.19823788546255502 | 0.3414096916299611 | 0.25330396475771205 | 0.209251101321586 | 0.26431718061674003 | 0.36343612334802006 | 0.19823788546255502 | 0.26431718061674003 | 0.209251101321586 | 0.209251101321586 | 0.275330396475771 | 0.275330396475771 | 0.25330396475771205 | 0.15418502202643203 | 0.3083700440528691 | 0.17621145374449607 | 0.3083700440528691 | 0.187224669603524 | 0.24229074889867802 | 0.15418502202643203 | 0.25330396475771205 | 0.15418502202643203 | 0.13215859030837 | 0.16519823788546606 | 0.13215859030837 | 0.15418502202643203 | 0.275330396475771 | 0.209251101321586 | 0.13215859030837 | 0.14317180616740102 | 0.3193832599118981 | 0.16519823788546606 | 0.15418502202643203 | 0.09911894273127872 | 0.14317180616740102 | 0.16519823788546606 | 0.220264317180617 | 0.16519823788546606 | 0.2863436123348071 | 0.187224669603524 | 0.16519823788546606 | 0.16519823788546606 | 0.15418502202643203 | 0.12114537444934001 | 0.13215859030837 |
| -24 | 0.682819383259912 | 0.583700440528634 | 0.3303964757709311 | 0.583700440528634 | 0.40748898678414713 | 0.209251101321586 | 0.3414096916299611 | 0.36343612334802006 | 0.3744493392070531 | 0.25330396475771205 | 0.2973568281938361 | 0.3744493392070531 | 0.2973568281938361 | 0.16519823788546606 | 0.220264317180617 | 0.2863436123348071 | 0.3524229074889901 | 0.2973568281938361 | 0.3193832599118981 | 0.26431718061674003 | 0.231277533039648 | 0.3193832599118981 | 0.19823788546255502 | 0.26431718061674003 | 0.19823788546255502 | 0.3193832599118981 | 0.3083700440528691 | 0.187224669603524 | 0.13215859030837 | 0.231277533039648 | 0.15418502202643203 | 0.25330396475771205 | 0.13215859030837 | 0.187224669603524 | 0.16519823788546606 | 0.220264317180617 | 0.17621145374449607 | 0.15418502202643203 | 0.16519823788546606 | 0.15418502202643203 | 0.17621145374449607 | 0.24229074889867802 | 0.231277533039648 | 0.14317180616740102 | 0.16519823788546606 | 0.13215859030837 | 0.187224669603524 | 0.16519823788546606 | 0.19823788546255502 | 0.209251101321586 |
| -23 | 0.8810572687224588 | 0.5396475770925121 | 0.42951541850220304 | 0.506607929515419 | 0.3744493392070531 | 0.3303964757709311 | 0.4845814977973621 | 0.46255506607929503 | 0.275330396475771 | 0.231277533039648 | 0.3193832599118981 | 0.3414096916299611 | 0.3083700440528691 | 0.275330396475771 | 0.3303964757709311 | 0.26431718061674003 | 0.3083700440528691 | 0.220264317180617 | 0.16519823788546606 | 0.26431718061674003 | 0.231277533039648 | 0.275330396475771 | 0.3303964757709311 | 0.25330396475771205 | 0.16519823788546606 | 0.26431718061674003 | 0.418502202643172 | 0.2973568281938361 | 0.220264317180617 | 0.187224669603524 | 0.231277533039648 | 0.14317180616740102 | 0.209251101321586 | 0.209251101321586 | 0.2973568281938361 | 0.24229074889867802 | 0.187224669603524 | 0.15418502202643203 | 0.14317180616740102 | 0.220264317180617 | 0.17621145374449607 | 0.2863436123348071 | 0.11013215859030799 | 0.25330396475771205 | 0.220264317180617 | 0.13215859030837 | 0.26431718061674003 | 0.13215859030837 | 0.11013215859030799 | 0.187224669603524 |
| -22 | 0.7599118942731392 | 0.5616740088105802 | 0.4845814977973621 | 0.583700440528634 | 0.3303964757709311 | 0.36343612334802006 | 0.4845814977973621 | 0.3303964757709311 | 0.3303964757709311 | 0.3414096916299611 | 0.25330396475771205 | 0.26431718061674003 | 0.2863436123348071 | 0.3193832599118981 | 0.3193832599118981 | 0.15418502202643203 | 0.220264317180617 | 0.2973568281938361 | 0.3303964757709311 | 0.2973568281938361 | 0.3083700440528691 | 0.220264317180617 | 0.25330396475771205 | 0.231277533039648 | 0.220264317180617 | 0.220264317180617 | 0.15418502202643203 | 0.231277533039648 | 0.2863436123348071 | 0.209251101321586 | 0.3083700440528691 | 0.12114537444934001 | 0.2973568281938361 | 0.231277533039648 | 0.187224669603524 | 0.19823788546255502 | 0.15418502202643203 | 0.24229074889867802 | 0.16519823788546606 | 0.2973568281938361 | 0.187224669603524 | 0.3083700440528691 | 0.09911894273127872 | 0.17621145374449607 | 0.11013215859030799 | 0.12114537444934001 | 0.17621145374449607 | 0.16519823788546606 | 0.15418502202643203 | 0.19823788546255502 |
| -21 | 0.825991189427313 | 0.40748898678414713 | 0.47356828193832906 | 0.47356828193832906 | 0.451541850220264 | 0.38546255506608307 | 0.5286343612334811 | 0.36343612334802006 | 0.42951541850220304 | 0.26431718061674003 | 0.19823788546255502 | 0.2863436123348071 | 0.2863436123348071 | 0.3744493392070531 | 0.3193832599118981 | 0.2863436123348071 | 0.220264317180617 | 0.3303964757709311 | 0.24229074889867802 | 0.220264317180617 | 0.209251101321586 | 0.187224669603524 | 0.187224669603524 | 0.17621145374449607 | 0.19823788546255502 | 0.209251101321586 | 0.13215859030837 | 0.209251101321586 | 0.2863436123348071 | 0.209251101321586 | 0.3964757709251151 | 0.04405286343612352 | 0.187224669603524 | 0.275330396475771 | 0.25330396475771205 | 0.16519823788546606 | 0.187224669603524 | 0.12114537444934001 | 0.16519823788546606 | 0.275330396475771 | 0.275330396475771 | 0.14317180616740102 | 0.055066079295154204 | 0.07709251101321722 | 0.11013215859030799 | 0.14317180616740102 | 0.220264317180617 | 0.231277533039648 | 0.187224669603524 | 0.187224669603524 |
| -20 | 0.550660792951542 | 0.3744493392070531 | 0.5616740088105802 | 0.5616740088105802 | 0.3964757709251151 | 0.40748898678414713 | 0.36343612334802006 | 0.2863436123348071 | 0.3964757709251151 | 0.3193832599118981 | 0.231277533039648 | 0.2973568281938361 | 0.231277533039648 | 0.3083700440528691 | 0.220264317180617 | 0.16519823788546606 | 0.19823788546255502 | 0.13215859030837 | 0.24229074889867802 | 0.17621145374449607 | 0.24229074889867802 | 0.19823788546255502 | 0.209251101321586 | 0.19823788546255502 | 0.25330396475771205 | 0.24229074889867802 | 0.12114537444934001 | 0.26431718061674003 | 0.17621145374449607 | 0.220264317180617 | 0.26431718061674003 | 0.055066079295154204 | 0.13215859030837 | 0.231277533039648 | 0.15418502202643203 | 0.209251101321586 | 0.275330396475771 | 0.24229074889867802 | 0.25330396475771205 | 0.209251101321586 | 0.15418502202643203 | 0.13215859030837 | 0.07709251101321722 | 0.12114537444934001 | 0.09911894273127872 | 0.19823788546255502 | 0.07709251101321722 | 0.13215859030837 | 0.08810572687224673 | 0.2973568281938361 |
| -19 | 0.506607929515419 | 0.275330396475771 | 0.4845814977973621 | 0.3744493392070531 | 0.275330396475771 | 0.781938325991189 | 0.3964757709251151 | 0.14317180616740102 | 0.3083700440528691 | 0.3083700440528691 | 0.25330396475771205 | 0.275330396475771 | 0.24229074889867802 | 0.42951541850220304 | 0.26431718061674003 | 0.2863436123348071 | 0.19823788546255502 | 0.15418502202643203 | 0.209251101321586 | 0.19823788546255502 | 0.231277533039648 | 0.220264317180617 | 0.26431718061674003 | 0.11013215859030799 | 0.24229074889867802 | 0.17621145374449607 | 0.15418502202643203 | 0.19823788546255502 | 0.187224669603524 | 0.275330396475771 | 0.12114537444934001 | 0.04405286343612352 | 0.17621145374449607 | 0.187224669603524 | 0.17621145374449607 | 0.14317180616740102 | 0.187224669603524 | 0.13215859030837 | 0.13215859030837 | 0.15418502202643203 | 0.15418502202643203 | 0.066079295154185 | 0.04405286343612352 | 0.09911894273127872 | 0.04405286343612352 | 0.13215859030837 | 0.04405286343612352 | 0.209251101321586 | 0.17621145374449607 | 0.12114537444934001 |
| -18 | 0.3193832599118981 | 0.3414096916299611 | 0.3193832599118981 | 0.36343612334802006 | 0.3414096916299611 | 0.550660792951542 | 0.231277533039648 | 0.11013215859030799 | 0.2973568281938361 | 0.3524229074889901 | 0.231277533039648 | 0.209251101321586 | 0.17621145374449607 | 0.3303964757709311 | 0.15418502202643203 | 0.15418502202643203 | 0.17621145374449607 | 0.14317180616740102 | 0.16519823788546606 | 0.12114537444934001 | 0.19823788546255502 | 0.17621145374449607 | 0.220264317180617 | 0.04405286343612352 | 0.15418502202643203 | 0.11013215859030799 | 0.11013215859030799 | 0.14317180616740102 | 0.17621145374449607 | 0.066079295154185 | 0.209251101321586 | 0.03303964757709291 | 0.187224669603524 | 0.2863436123348071 | 0.14317180616740102 | 0.187224669603524 | 0.231277533039648 | 0.14317180616740102 | 0.19823788546255502 | 0.11013215859030799 | 0.19823788546255502 | 0.066079295154185 | 0.11013215859030799 | 0.11013215859030799 | 0.066079295154185 | 0.13215859030837 | 0.11013215859030799 | 0.17621145374449607 | 0.17621145374449607 | 0.14317180616740102 |F Selaginella moellendorffii (spikemoss)
G Physcomitrella patens (moss)
### Chart
| Category | AAUAAA | AAAUAA | AUAAAA | AUAAAU | AAUGAA | UGAAAU | UUUUAA | UUUAAU | AAUUUU | AUUUUU | UAAUAA | UUUGAA | AGUAAA | AAAUUU | UAAAUA | UGUAAU | AUAUAU | UUAUUU | GAAUAA | AAUACA | UUCAAU | UUGAAU | AUAAAG | AAUUUA | AUGAAA | AAUUGA | AUUUUA | UUUAUU | UAAUUU | UAAAUU | UGAAUA | UUGUAA | UAAAAU | AUUUAU | AUUGAA | AAUAUA | UUUUUU | AAAAUU | AUUUAA | UUUUUA | UUUAAA | UAAAAA | AUAAUA | AUCAAU | UUUUAU | UUAAUU | UAUAAA | UUAAUA | AAAAAU | UAAUAU |
|---|---|---|---|---|---|---|---|---|---|---|---|---|---|---|---|---|---|---|---|---|---|---|---|---|---|---|---|---|---|---|---|---|---|---|---|---|---|---|---|---|---|---|---|---|---|---|---|---|---|---|
| -35 | 0.21390374331550802 | 0.106951871657754 | 0.047534165181224004 | 0.11883541295306001 | 0.106951871657754 | 0.23767082590611796 | 0.26143790849672893 | 0.106951871657754 | 0.15448603683898005 | 0.26143790849672893 | 0.106951871657754 | 0.21390374331550802 | 0.08318478906714202 | 0.13071895424836602 | 0.07130124777183602 | 0.20202020202020202 | 0.0950683303624492 | 0.166369578134284 | 0.13071895424836602 | 0.08318478906714202 | 0.07130124777183602 | 0.17825311942959002 | 0.08318478906714202 | 0.11883541295306001 | 0.08318478906714202 | 0.13071895424836602 | 0.166369578134284 | 0.166369578134284 | 0.13071895424836602 | 0.0950683303624492 | 0.07130124777183602 | 0.14260249554367202 | 0.15448603683898005 | 0.17825311942959002 | 0.08318478906714202 | 0.07130124777183602 | 0.190136660724896 | 0.166369578134284 | 0.13071895424836602 | 0.14260249554367202 | 0.15448603683898005 | 0.106951871657754 | 0.11883541295306001 | 0.13071895424836602 | 0.0950683303624492 | 0.20202020202020202 | 0.07130124777183602 | 0.07130124777183602 | 0.059417706476530496 | 0.03565062388591801 |
| -34 | 0.29708853238265714 | 0.20202020202020202 | 0.13071895424836602 | 0.15448603683898005 | 0.190136660724896 | 0.20202020202020202 | 0.20202020202020202 | 0.21390374331550802 | 0.15448603683898005 | 0.20202020202020202 | 0.08318478906714202 | 0.190136660724896 | 0.08318478906714202 | 0.190136660724896 | 0.08318478906714202 | 0.166369578134284 | 0.13071895424836602 | 0.17825311942959002 | 0.047534165181224004 | 0.11883541295306001 | 0.21390374331550802 | 0.166369578134284 | 0.047534165181224004 | 0.08318478906714202 | 0.106951871657754 | 0.106951871657754 | 0.22578728461081402 | 0.08318478906714202 | 0.21390374331550802 | 0.11883541295306001 | 0.190136660724896 | 0.17825311942959002 | 0.08318478906714202 | 0.17825311942959002 | 0.11883541295306001 | 0.07130124777183602 | 0.106951871657754 | 0.106951871657754 | 0.11883541295306001 | 0.20202020202020202 | 0.15448603683898005 | 0.059417706476530496 | 0.07130124777183602 | 0.08318478906714202 | 0.15448603683898005 | 0.106951871657754 | 0.023767082590612 | 0.11883541295306001 | 0.20202020202020202 | 0.13071895424836602 |
| -33 | 0.22578728461081402 | 0.106951871657754 | 0.15448603683898005 | 0.11883541295306001 | 0.20202020202020202 | 0.190136660724896 | 0.11883541295306001 | 0.166369578134284 | 0.166369578134284 | 0.190136660724896 | 0.106951871657754 | 0.22578728461081402 | 0.0950683303624492 | 0.20202020202020202 | 0.20202020202020202 | 0.15448603683898005 | 0.11883541295306001 | 0.11883541295306001 | 0.15448603683898005 | 0.15448603683898005 | 0.166369578134284 | 0.21390374331550802 | 0.059417706476530496 | 0.23767082590611796 | 0.07130124777183602 | 0.15448603683898005 | 0.11883541295306001 | 0.11883541295306001 | 0.106951871657754 | 0.0950683303624492 | 0.11883541295306001 | 0.11883541295306001 | 0.0950683303624492 | 0.106951871657754 | 0.0950683303624492 | 0.07130124777183602 | 0.166369578134284 | 0.13071895424836602 | 0.106951871657754 | 0.14260249554367202 | 0.08318478906714202 | 0.08318478906714202 | 0.0950683303624492 | 0.106951871657754 | 0.24955436720142804 | 0.11883541295306001 | 0.11883541295306001 | 0.0950683303624492 | 0.11883541295306001 | 0.08318478906714202 |
| -32 | 0.26143790849672893 | 0.20202020202020202 | 0.15448603683898005 | 0.106951871657754 | 0.0950683303624492 | 0.166369578134284 | 0.190136660724896 | 0.190136660724896 | 0.17825311942959002 | 0.17825311942959002 | 0.13071895424836602 | 0.23767082590611796 | 0.166369578134284 | 0.13071895424836602 | 0.14260249554367202 | 0.190136660724896 | 0.07130124777183602 | 0.106951871657754 | 0.106951871657754 | 0.106951871657754 | 0.190136660724896 | 0.22578728461081402 | 0.106951871657754 | 0.190136660724896 | 0.20202020202020202 | 0.190136660724896 | 0.190136660724896 | 0.24955436720142804 | 0.17825311942959002 | 0.0950683303624492 | 0.15448603683898005 | 0.15448603683898005 | 0.14260249554367202 | 0.166369578134284 | 0.11883541295306001 | 0.047534165181224004 | 0.17825311942959002 | 0.047534165181224004 | 0.166369578134284 | 0.106951871657754 | 0.14260249554367202 | 0.106951871657754 | 0.07130124777183602 | 0.08318478906714202 | 0.106951871657754 | 0.13071895424836602 | 0.14260249554367202 | 0.11883541295306001 | 0.106951871657754 | 0.08318478906714202 |
| -31 | 0.2852049910873491 | 0.20202020202020202 | 0.11883541295306001 | 0.14260249554367202 | 0.15448603683898005 | 0.3208556149732661 | 0.11883541295306001 | 0.27332144979203804 | 0.2852049910873491 | 0.14260249554367202 | 0.14260249554367202 | 0.166369578134284 | 0.14260249554367202 | 0.047534165181224004 | 0.11883541295306001 | 0.22578728461081402 | 0.23767082590611796 | 0.20202020202020202 | 0.15448603683898005 | 0.11883541295306001 | 0.14260249554367202 | 0.14260249554367202 | 0.13071895424836602 | 0.106951871657754 | 0.0950683303624492 | 0.14260249554367202 | 0.166369578134284 | 0.106951871657754 | 0.166369578134284 | 0.08318478906714202 | 0.20202020202020202 | 0.190136660724896 | 0.15448603683898005 | 0.17825311942959002 | 0.14260249554367202 | 0.0950683303624492 | 0.13071895424836602 | 0.17825311942959002 | 0.166369578134284 | 0.23767082590611796 | 0.11883541295306001 | 0.11883541295306001 | 0.166369578134284 | 0.166369578134284 | 0.11883541295306001 | 0.08318478906714202 | 0.106951871657754 | 0.166369578134284 | 0.047534165181224004 | 0.11883541295306001 |
| -30 | 0.3446226975638781 | 0.23767082590611796 | 0.13071895424836602 | 0.20202020202020202 | 0.11883541295306001 | 0.17825311942959002 | 0.27332144979203804 | 0.20202020202020202 | 0.11883541295306001 | 0.20202020202020202 | 0.23767082590611796 | 0.11883541295306001 | 0.17825311942959002 | 0.08318478906714202 | 0.190136660724896 | 0.190136660724896 | 0.24955436720142804 | 0.20202020202020202 | 0.21390374331550802 | 0.166369578134284 | 0.15448603683898005 | 0.17825311942959002 | 0.059417706476530496 | 0.166369578134284 | 0.08318478906714202 | 0.13071895424836602 | 0.190136660724896 | 0.24955436720142804 | 0.15448603683898005 | 0.20202020202020202 | 0.11883541295306001 | 0.190136660724896 | 0.0950683303624492 | 0.0950683303624492 | 0.190136660724896 | 0.0950683303624492 | 0.22578728461081402 | 0.15448603683898005 | 0.13071895424836602 | 0.17825311942959002 | 0.13071895424836602 | 0.07130124777183602 | 0.13071895424836602 | 0.166369578134284 | 0.21390374331550802 | 0.15448603683898005 | 0.0950683303624492 | 0.17825311942959002 | 0.08318478906714202 | 0.13071895424836602 |
| -29 | 0.558526440879382 | 0.3446226975638781 | 0.13071895424836602 | 0.21390374331550802 | 0.190136660724896 | 0.14260249554367202 | 0.22578728461081402 | 0.24955436720142804 | 0.166369578134284 | 0.166369578134284 | 0.166369578134284 | 0.14260249554367202 | 0.23767082590611796 | 0.190136660724896 | 0.21390374331550802 | 0.106951871657754 | 0.0950683303624492 | 0.20202020202020202 | 0.17825311942959002 | 0.17825311942959002 | 0.24955436720142804 | 0.11883541295306001 | 0.15448603683898005 | 0.023767082590612 | 0.15448603683898005 | 0.15448603683898005 | 0.14260249554367202 | 0.14260249554367202 | 0.11883541295306001 | 0.14260249554367202 | 0.166369578134284 | 0.14260249554367202 | 0.0950683303624492 | 0.15448603683898005 | 0.14260249554367202 | 0.17825311942959002 | 0.166369578134284 | 0.13071895424836602 | 0.190136660724896 | 0.106951871657754 | 0.20202020202020202 | 0.13071895424836602 | 0.059417706476530496 | 0.14260249554367202 | 0.166369578134284 | 0.13071895424836602 | 0.047534165181224004 | 0.190136660724896 | 0.0950683303624492 | 0.190136660724896 |
| -28 | 0.4872251931075501 | 0.27332144979203804 | 0.20202020202020202 | 0.190136660724896 | 0.20202020202020202 | 0.17825311942959002 | 0.190136660724896 | 0.23767082590611796 | 0.17825311942959002 | 0.20202020202020202 | 0.166369578134284 | 0.106951871657754 | 0.166369578134284 | 0.166369578134284 | 0.20202020202020202 | 0.15448603683898005 | 0.07130124777183602 | 0.14260249554367202 | 0.24955436720142804 | 0.14260249554367202 | 0.14260249554367202 | 0.14260249554367202 | 0.13071895424836602 | 0.15448603683898005 | 0.24955436720142804 | 0.08318478906714202 | 0.14260249554367202 | 0.0950683303624492 | 0.13071895424836602 | 0.11883541295306001 | 0.11883541295306001 | 0.17825311942959002 | 0.11883541295306001 | 0.059417706476530496 | 0.13071895424836602 | 0.26143790849672893 | 0.14260249554367202 | 0.0950683303624492 | 0.0950683303624492 | 0.21390374331550802 | 0.13071895424836602 | 0.11883541295306001 | 0.13071895424836602 | 0.11883541295306001 | 0.11883541295306001 | 0.11883541295306001 | 0.14260249554367202 | 0.21390374331550802 | 0.190136660724896 | 0.0950683303624492 |
| -27 | 0.439691027926322 | 0.27332144979203804 | 0.26143790849672893 | 0.190136660724896 | 0.21390374331550802 | 0.26143790849672893 | 0.24955436720142804 | 0.27332144979203804 | 0.106951871657754 | 0.07130124777183602 | 0.20202020202020202 | 0.17825311942959002 | 0.22578728461081402 | 0.106951871657754 | 0.24955436720142804 | 0.21390374331550802 | 0.14260249554367202 | 0.106951871657754 | 0.15448603683898005 | 0.11883541295306001 | 0.2852049910873491 | 0.15448603683898005 | 0.15448603683898005 | 0.23767082590611796 | 0.24955436720142804 | 0.15448603683898005 | 0.15448603683898005 | 0.08318478906714202 | 0.15448603683898005 | 0.106951871657754 | 0.20202020202020202 | 0.24955436720142804 | 0.11883541295306001 | 0.0950683303624492 | 0.11883541295306001 | 0.106951871657754 | 0.0950683303624492 | 0.14260249554367202 | 0.15448603683898005 | 0.13071895424836602 | 0.0950683303624492 | 0.166369578134284 | 0.21390374331550802 | 0.20202020202020202 | 0.20202020202020202 | 0.166369578134284 | 0.166369578134284 | 0.14260249554367202 | 0.14260249554367202 | 0.13071895424836602 |
| -26 | 0.40404040404040403 | 0.332739156268568 | 0.190136660724896 | 0.22578728461081402 | 0.23767082590611796 | 0.3446226975638781 | 0.27332144979203804 | 0.190136660724896 | 0.15448603683898005 | 0.13071895424836602 | 0.22578728461081402 | 0.14260249554367202 | 0.15448603683898005 | 0.15448603683898005 | 0.17825311942959002 | 0.22578728461081402 | 0.13071895424836602 | 0.13071895424836602 | 0.21390374331550802 | 0.106951871657754 | 0.15448603683898005 | 0.17825311942959002 | 0.23767082590611796 | 0.15448603683898005 | 0.15448603683898005 | 0.11883541295306001 | 0.13071895424836602 | 0.24955436720142804 | 0.13071895424836602 | 0.059417706476530496 | 0.14260249554367202 | 0.14260249554367202 | 0.190136660724896 | 0.166369578134284 | 0.15448603683898005 | 0.13071895424836602 | 0.106951871657754 | 0.13071895424836602 | 0.17825311942959002 | 0.14260249554367202 | 0.11883541295306001 | 0.15448603683898005 | 0.190136660724896 | 0.21390374331550802 | 0.0950683303624492 | 0.14260249554367202 | 0.26143790849672893 | 0.17825311942959002 | 0.15448603683898005 | 0.15448603683898005 |
| -25 | 0.6535947712418311 | 0.21390374331550802 | 0.24955436720142804 | 0.30897207367796314 | 0.21390374331550802 | 0.166369578134284 | 0.190136660724896 | 0.2852049910873491 | 0.166369578134284 | 0.106951871657754 | 0.2852049910873491 | 0.13071895424836602 | 0.2852049910873491 | 0.08318478906714202 | 0.15448603683898005 | 0.190136660724896 | 0.24955436720142804 | 0.11883541295306001 | 0.14260249554367202 | 0.166369578134284 | 0.190136660724896 | 0.20202020202020202 | 0.15448603683898005 | 0.15448603683898005 | 0.15448603683898005 | 0.0950683303624492 | 0.14260249554367202 | 0.166369578134284 | 0.0950683303624492 | 0.2852049910873491 | 0.13071895424836602 | 0.106951871657754 | 0.11883541295306001 | 0.17825311942959002 | 0.15448603683898005 | 0.14260249554367202 | 0.047534165181224004 | 0.14260249554367202 | 0.15448603683898005 | 0.14260249554367202 | 0.20202020202020202 | 0.106951871657754 | 0.27332144979203804 | 0.13071895424836602 | 0.14260249554367202 | 0.17825311942959002 | 0.11883541295306001 | 0.20202020202020202 | 0.106951871657754 | 0.15448603683898005 |
| -24 | 0.5347593582887792 | 0.3208556149732661 | 0.332739156268568 | 0.17825311942959002 | 0.29708853238265714 | 0.17825311942959002 | 0.20202020202020202 | 0.21390374331550802 | 0.059417706476530496 | 0.11883541295306001 | 0.29708853238265714 | 0.17825311942959002 | 0.20202020202020202 | 0.3208556149732661 | 0.15448603683898005 | 0.166369578134284 | 0.23767082590611796 | 0.106951871657754 | 0.14260249554367202 | 0.21390374331550802 | 0.106951871657754 | 0.14260249554367202 | 0.190136660724896 | 0.13071895424836602 | 0.13071895424836602 | 0.17825311942959002 | 0.14260249554367202 | 0.14260249554367202 | 0.166369578134284 | 0.17825311942959002 | 0.23767082590611796 | 0.15448603683898005 | 0.22578728461081402 | 0.08318478906714202 | 0.17825311942959002 | 0.22578728461081402 | 0.08318478906714202 | 0.13071895424836602 | 0.106951871657754 | 0.08318478906714202 | 0.190136660724896 | 0.20202020202020202 | 0.11883541295306001 | 0.20202020202020202 | 0.08318478906714202 | 0.17825311942959002 | 0.0950683303624492 | 0.166369578134284 | 0.0950683303624492 | 0.23767082590611796 |
| -23 | 0.6654783125371442 | 0.190136660724896 | 0.22578728461081402 | 0.17825311942959002 | 0.24955436720142804 | 0.17825311942959002 | 0.166369578134284 | 0.166369578134284 | 0.23767082590611796 | 0.14260249554367202 | 0.23767082590611796 | 0.14260249554367202 | 0.15448603683898005 | 0.190136660724896 | 0.17825311942959002 | 0.0950683303624492 | 0.20202020202020202 | 0.0950683303624492 | 0.17825311942959002 | 0.166369578134284 | 0.13071895424836602 | 0.0950683303624492 | 0.166369578134284 | 0.20202020202020202 | 0.13071895424836602 | 0.21390374331550802 | 0.07130124777183602 | 0.07130124777183602 | 0.106951871657754 | 0.14260249554367202 | 0.11883541295306001 | 0.14260249554367202 | 0.17825311942959002 | 0.17825311942959002 | 0.14260249554367202 | 0.21390374331550802 | 0.059417706476530496 | 0.11883541295306001 | 0.11883541295306001 | 0.14260249554367202 | 0.106951871657754 | 0.166369578134284 | 0.21390374331550802 | 0.14260249554367202 | 0.0950683303624492 | 0.166369578134284 | 0.190136660724896 | 0.106951871657754 | 0.20202020202020202 | 0.0950683303624492 |
| -22 | 0.5109922756981591 | 0.3208556149732661 | 0.26143790849672893 | 0.26143790849672893 | 0.23767082590611796 | 0.11883541295306001 | 0.106951871657754 | 0.0950683303624492 | 0.166369578134284 | 0.047534165181224004 | 0.21390374331550802 | 0.11883541295306001 | 0.166369578134284 | 0.106951871657754 | 0.14260249554367202 | 0.08318478906714202 | 0.13071895424836602 | 0.059417706476530496 | 0.15448603683898005 | 0.21390374331550802 | 0.0950683303624492 | 0.106951871657754 | 0.24955436720142804 | 0.15448603683898005 | 0.17825311942959002 | 0.20202020202020202 | 0.166369578134284 | 0.0950683303624492 | 0.190136660724896 | 0.166369578134284 | 0.14260249554367202 | 0.08318478906714202 | 0.13071895424836602 | 0.14260249554367202 | 0.13071895424836602 | 0.190136660724896 | 0.08318478906714202 | 0.20202020202020202 | 0.20202020202020202 | 0.047534165181224004 | 0.11883541295306001 | 0.11883541295306001 | 0.17825311942959002 | 0.106951871657754 | 0.13071895424836602 | 0.166369578134284 | 0.17825311942959002 | 0.106951871657754 | 0.14260249554367202 | 0.14260249554367202 |
| -21 | 0.558526440879382 | 0.20202020202020202 | 0.15448603683898005 | 0.17825311942959002 | 0.21390374331550802 | 0.11883541295306001 | 0.0950683303624492 | 0.14260249554367202 | 0.21390374331550802 | 0.190136660724896 | 0.14260249554367202 | 0.0950683303624492 | 0.15448603683898005 | 0.22578728461081402 | 0.14260249554367202 | 0.11883541295306001 | 0.023767082590612 | 0.190136660724896 | 0.14260249554367202 | 0.20202020202020202 | 0.106951871657754 | 0.059417706476530496 | 0.22578728461081402 | 0.15448603683898005 | 0.190136660724896 | 0.0950683303624492 | 0.106951871657754 | 0.08318478906714202 | 0.07130124777183602 | 0.13071895424836602 | 0.106951871657754 | 0.08318478906714202 | 0.17825311942959002 | 0.11883541295306001 | 0.14260249554367202 | 0.106951871657754 | 0.11883541295306001 | 0.166369578134284 | 0.08318478906714202 | 0.059417706476530496 | 0.106951871657754 | 0.17825311942959002 | 0.059417706476530496 | 0.08318478906714202 | 0.11883541295306001 | 0.03565062388591801 | 0.17825311942959002 | 0.08318478906714202 | 0.059417706476530496 | 0.190136660724896 |
| -20 | 0.5228758169934753 | 0.13071895424836602 | 0.24955436720142804 | 0.190136660724896 | 0.13071895424836602 | 0.15448603683898005 | 0.07130124777183602 | 0.03565062388591801 | 0.20202020202020202 | 0.14260249554367202 | 0.059417706476530496 | 0.11883541295306001 | 0.0950683303624492 | 0.106951871657754 | 0.08318478906714202 | 0.07130124777183602 | 0.106951871657754 | 0.11883541295306001 | 0.13071895424836602 | 0.15448603683898005 | 0.106951871657754 | 0.13071895424836602 | 0.17825311942959002 | 0.106951871657754 | 0.13071895424836602 | 0.22578728461081402 | 0.13071895424836602 | 0.14260249554367202 | 0.13071895424836602 | 0.15448603683898005 | 0.08318478906714202 | 0.08318478906714202 | 0.059417706476530496 | 0.08318478906714202 | 0.13071895424836602 | 0.20202020202020202 | 0.13071895424836602 | 0.0950683303624492 | 0.0950683303624492 | 0.059417706476530496 | 0.0950683303624492 | 0.14260249554367202 | 0.106951871657754 | 0.08318478906714202 | 0.08318478906714202 | 0.13071895424836602 | 0.15448603683898005 | 0.08318478906714202 | 0.14260249554367202 | 0.0950683303624492 |
| -19 | 0.24955436720142804 | 0.08318478906714202 | 0.17825311942959002 | 0.23767082590611796 | 0.106951871657754 | 0.08318478906714202 | 0.106951871657754 | 0.047534165181224004 | 0.190136660724896 | 0.24955436720142804 | 0.0950683303624492 | 0.08318478906714202 | 0.047534165181224004 | 0.14260249554367202 | 0.106951871657754 | 0.106951871657754 | 0.21390374331550802 | 0.15448603683898005 | 0.08318478906714202 | 0.15448603683898005 | 0.07130124777183602 | 0.0950683303624492 | 0.190136660724896 | 0.106951871657754 | 0.07130124777183602 | 0.11883541295306001 | 0.11883541295306001 | 0.14260249554367202 | 0.166369578134284 | 0.13071895424836602 | 0.0950683303624492 | 0.07130124777183602 | 0.11883541295306001 | 0.166369578134284 | 0.13071895424836602 | 0.106951871657754 | 0.0950683303624492 | 0.106951871657754 | 0.0950683303624492 | 0.08318478906714202 | 0.08318478906714202 | 0.20202020202020202 | 0.059417706476530496 | 0.047534165181224004 | 0.047534165181224004 | 0.03565062388591801 | 0.13071895424836602 | 0.011883541295306303 | 0.17825311942959002 | 0.11883541295306001 |
| -18 | 0.22578728461081402 | 0.106951871657754 | 0.15448603683898005 | 0.0950683303624492 | 0.13071895424836602 | 0.047534165181224004 | 0.14260249554367202 | 0.059417706476530496 | 0.21390374331550802 | 0.26143790849672893 | 0.03565062388591801 | 0.14260249554367202 | 0.0950683303624492 | 0.14260249554367202 | 0.0950683303624492 | 0.03565062388591801 | 0.15448603683898005 | 0.22578728461081402 | 0.03565062388591801 | 0.047534165181224004 | 0.03565062388591801 | 0.08318478906714202 | 0.0950683303624492 | 0.08318478906714202 | 0.13071895424836602 | 0.059417706476530496 | 0.047534165181224004 | 0.14260249554367202 | 0.08318478906714202 | 0.190136660724896 | 0.07130124777183602 | 0.059417706476530496 | 0.17825311942959002 | 0.106951871657754 | 0.11883541295306001 | 0.08318478906714202 | 0.26143790849672893 | 0.14260249554367202 | 0.059417706476530496 | 0.13071895424836602 | 0.11883541295306001 | 0.0950683303624492 | 0.059417706476530496 | 0.0950683303624492 | 0.07130124777183602 | 0.03565062388591801 | 0.059417706476530496 | 0.023767082590612 | 0.13071895424836602 | 0.047534165181224004 |Position

## Slide 3
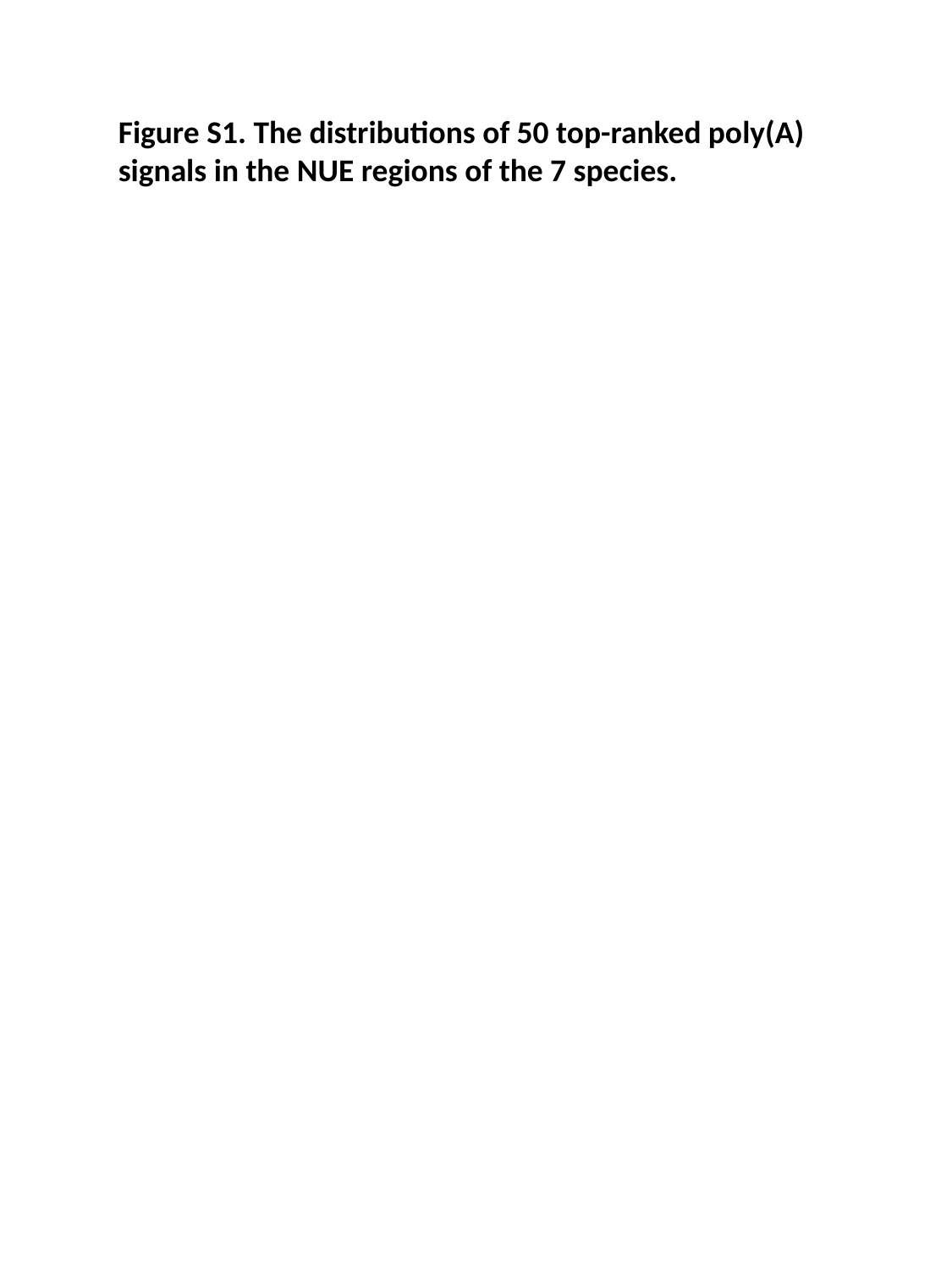

Figure S1. The distributions of 50 top-ranked poly(A) signals in the NUE regions of the 7 species.
