# Supplementary material for: Genome-Wide Comparative Analyses of Polyadenylation Signals in Eukaryotes Suggest a Possible Origin of the AAUAAA Signal
Source: Int J Mol Sci. 2019 Feb 22;20(4):958. doi: 10.3390/ijms20040958 (PMC6413133; doi:10.3390/ijms20040958)
Supplement: Supplementary file 1 [file ijms-20-00958-s001.zip › ijms-444287 suppl final/Appendix Figures and Tables-revised/Figure S3.pptx]

## Slide 1
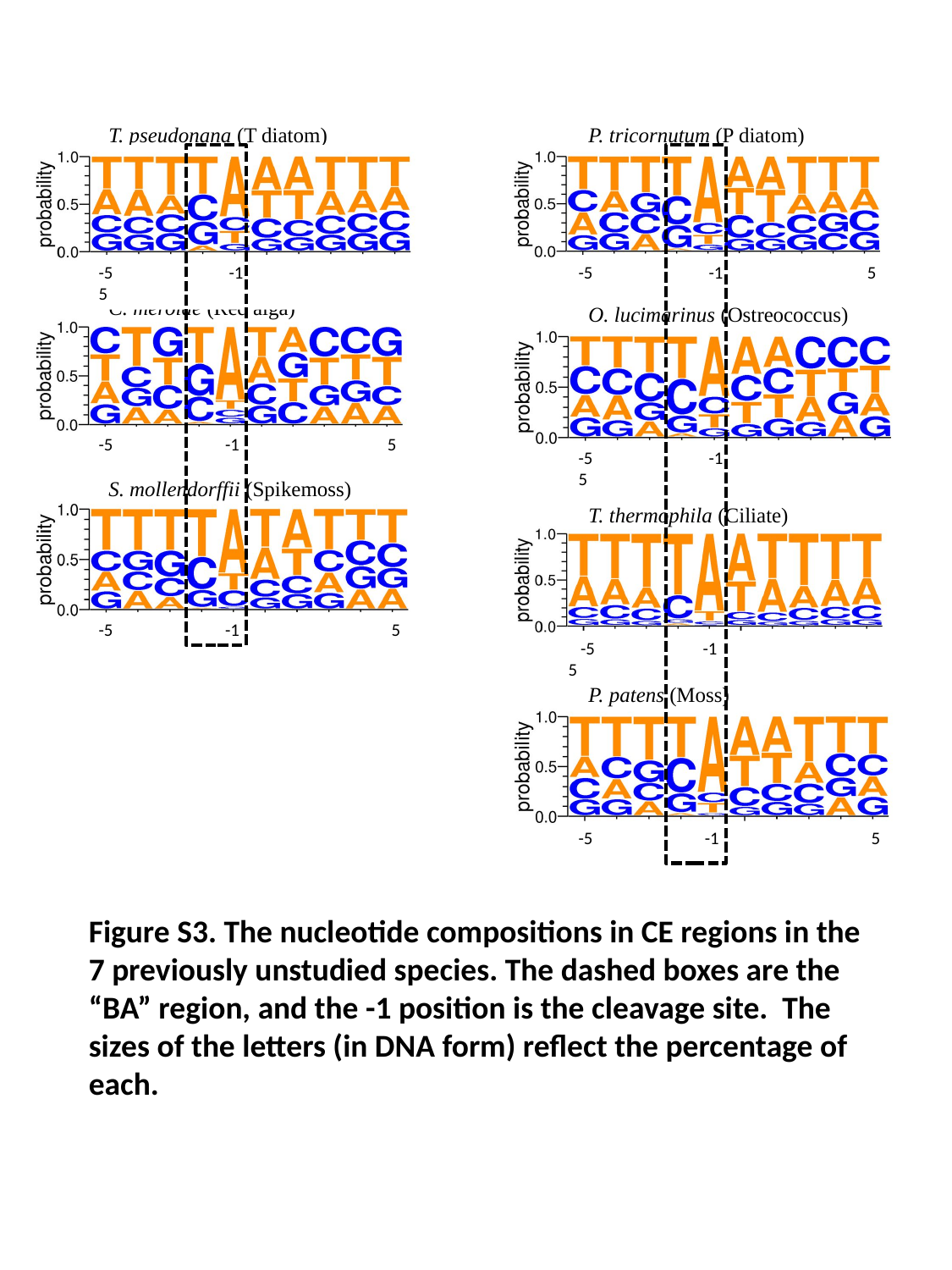

T. pseudonana (T diatom)
P. tricornutum (P diatom)
-5 -1 5
-5 -1 5
C. merolae (Red alga)
O. lucimarinus (Ostreococcus)
-5 -1 5
-5 -1 5
S. mollendorffii (Spikemoss)
T. thermophila (Ciliate)
-5 -1 5
 -5 -1 5
P. patens (Moss)
-5 -1 5
Figure S3. The nucleotide compositions in CE regions in the 7 previously unstudied species. The dashed boxes are the “BA” region, and the -1 position is the cleavage site. The sizes of the letters (in DNA form) reflect the percentage of each.
