# Supplementary material for: Genome-Wide Comparative Analyses of Polyadenylation Signals in Eukaryotes Suggest a Possible Origin of the AAUAAA Signal
Source: Int J Mol Sci. 2019 Feb 22;20(4):958. doi: 10.3390/ijms20040958 (PMC6413133; doi:10.3390/ijms20040958)
Supplement: Supplementary file 1 [file ijms-20-00958-s001.zip › ijms-444287 suppl final/Appendix Figures and Tables-revised/Figure S4.pptx]

## Slide 1
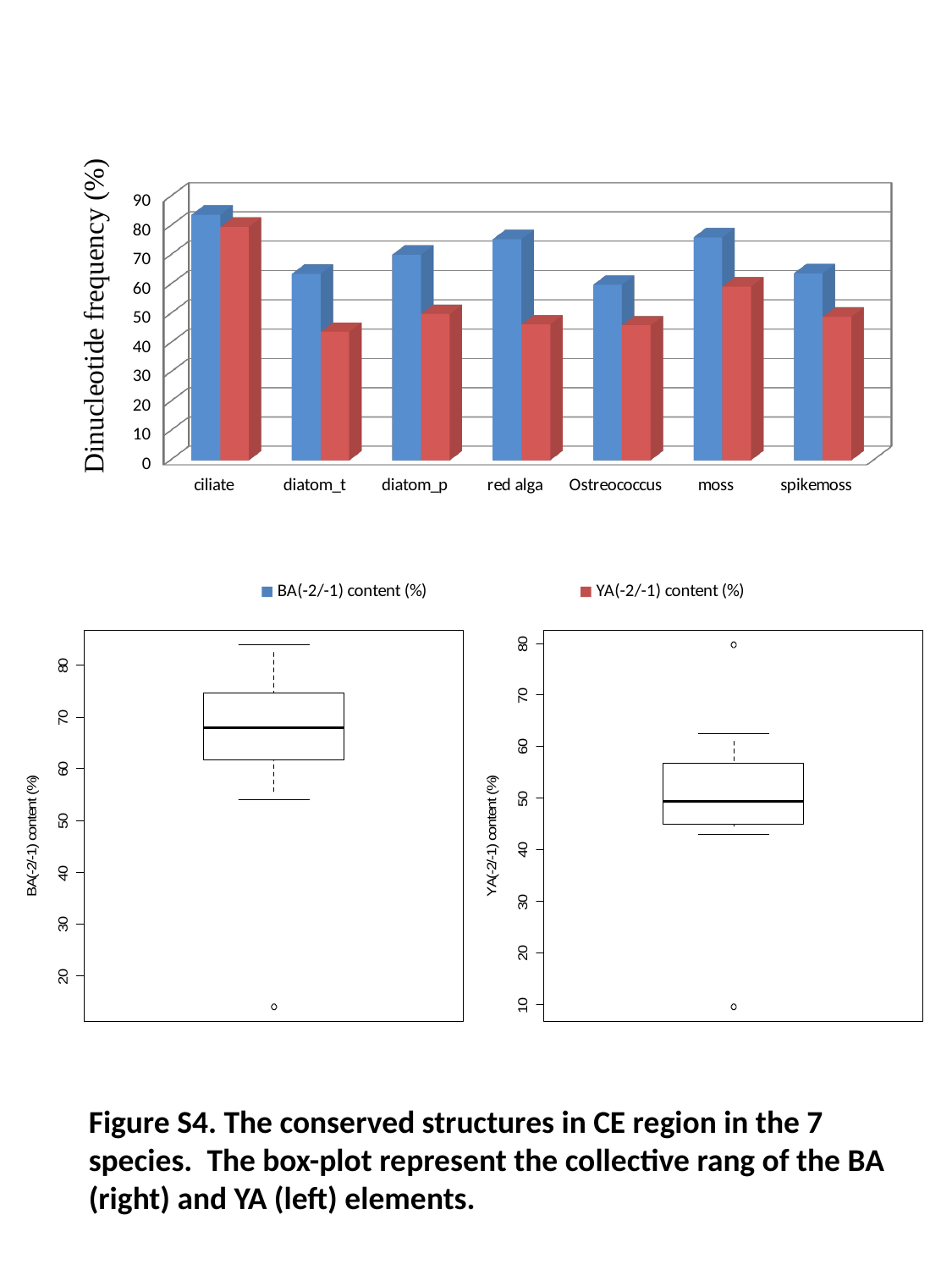

Dinucleotide frequency (%)
[unsupported chart]
Figure S4. The conserved structures in CE region in the 7 species. The box-plot represent the collective rang of the BA (right) and YA (left) elements.
