# Supplementary material for: Genome-Wide Comparative Analyses of Polyadenylation Signals in Eukaryotes Suggest a Possible Origin of the AAUAAA Signal
Source: Int J Mol Sci. 2019 Feb 22;20(4):958. doi: 10.3390/ijms20040958 (PMC6413133; doi:10.3390/ijms20040958)
Supplement: Supplementary file 1 [file ijms-20-00958-s001.zip › ijms-444287 suppl final/Appendix Figures and Tables-revised/Table S1.docx]

**Table S1. The percentages of four nucleotides in 7 previously unstudied species**

| **Species name (common name)** | **Whole region (%)** | **FUE (%)** | **NUE (%)** | **CE (%)** |
| --- | --- | --- | --- | --- |
| ***T. pseudonana***  **(T diatom)** | -300/+100  A **28.13**  U 27.74  G 24.98  C 19.15 | -200/-35  A 28.01  U 26.94  G **28.07**  C 16.98 | -35/-15  A **38.76**  U 30.14  G 17.56  C 13.54 | -15/+10  A 29.18  U **33.25**  G 18.17  C 19.30 |
| ***P. tricornutum***  **(P diatom)** | -300/+100  A **27.10**  U 26.84  G 23.70  C 22.36 | -200/-40  A **27.04**  U 25.31  G 24.97  C 22.68 | -40/-15  A **36.19**  U 29.95  G 17.07  C 16.79 | -15/+10  A 26.86  U **35.08**  G 17.98  C 20.08 |
| ***T. thermophila* (Ciliate)** | -300/+100  A **39.33**  U 39.20  G 10.16  C 11.31 | -80/-20  A 35.33  U **47.34**  G 8.44  C 8.89 | -20/-5  A 39.43  U **42.65**  G 6.74  C 11.18 | -5/+10  A 31.86  U **48.71**  G 5.73  C 13.70 |
| ***O. lucimarinus* (Ostreococcus)** | -300/+100  A 22.23  U 21.78  G 26.76  **C 29.22** | -200/-35  A 22.51  U 21.06  G 27.88  **C 28.55** | -35/-15  A 31.70  U **31.87**  G 17.28  C 19.14 | -15/+10  A 26.38  U 27.42  G 18.23  C **27.97** |
| ***C. merolae***  **(Red alga)** | -300/+100  A 22.67  U 23.01  G **27.74**  C 26.58 | -200/-30  A 22.47  U 22.87  G **28.85**  C 25.81 | -30/-10  A **32.87**  U 23.23  G 22.15  C 21.75 | -10/+10  A 21.65  U **31.06**  G 23.03  C 24.26 |
| ***S. moellendorffii* (Spikemoss)** | -300/+100  A 25.84  U **27.29**  G 24.44  C 22.43 | -200/-32  A 26.17  U **26.62**  G 24.93  C 22.48 | -32/-13  A **37.20**  U 28.93  G 17.21  C 16.64 | -13/+10  A 23.14  U **38.10**  G 18.41  C 20.34 |
| ***P. patens***  **(Moss)** | -300/+100  A 25.65  U **32.48**  G 22.68  C 19.19 | -200/-35  A 25.90  U **31.63**  G 23.56  C 18.91 | -35/-13  A 31.64  U **33.22**  G 18.02  C 17.12 | -13/+10  A 23.44  U **39.40**  G 17.39  C 19.77 |
