# Supplementary material for: Genome-Wide Comparative Analyses of Polyadenylation Signals in Eukaryotes Suggest a Possible Origin of the AAUAAA Signal
Source: Int J Mol Sci. 2019 Feb 22;20(4):958. doi: 10.3390/ijms20040958 (PMC6413133; doi:10.3390/ijms20040958)
Supplement: Supplementary file 1 [file ijms-20-00958-s001.zip › ijms-444287 suppl final/Appendix Figures and Tables-revised/Table S9.docx]

**Table S9. The data sources of the 11 species for poly(A) signal analysis**

| **Species name (common name)** | **EST source** | Genome source | **No. ESTs** | **Poly(A) sites** |
| --- | --- | --- | --- | --- |
| ***Thalassiosira pseudonana* (T diatom)** | Genbank, JGI | V3.0, JGI | 76,319 | 2,943 |
| ***Phaeodactylum tricornutum* (P diatom)** | Genbank, JGI | V2.0, JGI | 207,560 | 3,520 |
| ***Tetrahymena thermophila* (Ciliate)** | Genbank, TGD | Tetrahymena  Genome Database | 103,511 | 2,729 |
| ***Chlamydomonas reinhardtii* (Chlamydomonas)** | JGI, Liang *et al.* (26) | V4.0, JGI | 338,234 | 21,037 |
| ***Ostreococcus lucimarinus* (Ostreococcus)** | Genbank, JGI | V2.0, JGI | 26,863 | 783 |
| ***Cyanidioschyzon merolae* (Red alga)** | *C. merolae* Genome Project | *C. merolae* Genome Project | 63,712 | 155 |
| ***Selaginella moellendorffii* (Spikemoss)** | Genbank, JGI | V1.0, JGI | 94,214 | 9,080 |
| ***Physcomitrella patens* (Moss)** | Genbank | V1.1, JGI | 382,584 | 8,415 |
| ***Arabidopsis thaliana* (Arabidopsis)** | Genbank | TAIR9.0 | 1,527,298 | 23,762 |
| ***Saccharomyces cerevisiae* (Yeast)** | Graber *et al.*(29) | SGD | 3,425 | 555 |
| ***Homo sapiens* (Human)** | Genbank | UCSC hg19 | 8,296,280 | 12,449 |
